# Competing dynamic gene regulatory networks involved in fibroblast reprogramming to hematopoietic progenitor cells

**DOI:** 10.1016/j.stemcr.2025.102473

**Published:** 2025-04-03

**Authors:** Samiyah Shafiq, Kiyofumi Hamashima, Laura A. Guest, Ali H. Al-anbaki, Fabio M.R. Amaral, Daniel H. Wiseman, Valerie Kouskoff, Georges Lacaud, Yuin-Han Loh, Kiran Batta

**Affiliations:** 1 Epigenetics of Haematopoiesis Laboratory, Division of Cancer Sciences, The University of Manchester, Manchester, UK; 2 Cell Fate Engineering and Therapeutics Lab, Cell Biology and Therapies Division, Institute of Molecular and Cell Biology (IMCB), Agency for Science, Technology and Research (A(∗)STAR), Singapore, Republic of Singapore; 3 Stem Cell Biology Group, Cancer Research UK Manchester Institute, The University of Manchester, Manchester, UK; 4 Leukaemia Biology Laboratory, Cancer Research UK Manchester Institute, The University of Manchester, Manchester, UK; 5 Developmental Haematopoiesis Group, Division of Developmental Biology and Medicine, The University of Manchester, Manchester, UK

**Keywords:** direct reprogramming, SCL, LMO2, hematopoietic progenitor cells, cell therapies, hemogenic endothelium, reprogramming stochasticity, neuronal fate

## Abstract

Direct reprogramming of somatic cells offers a potentially safer therapeutic approach to generate patient-specific hematopoietic cells. However, this strategy is limited by stochasticity of reprogramming. Investigating the gene regulatory networks involved during reprogramming would help generate functional cells in adequate numbers. To address this, we developed an inducible system to reprogram fibroblasts to hematopoietic progenitor cells by ectopically expressing the two transcription factors SCL and LMO2. Transcriptome and epigenome analysis at different stages of reprogramming revealed uniform silencing of fibroblast genes and upregulation of the hemogenic endothelial program. Integrated analysis suggested that the transcription factors FLI1, GATA1/2, and KLF14 are direct targets of SCL/LMO2, which subsequently induce the hematopoietic program. Single-cell RNA sequencing revealed conflicting and competing fate decisions at intermediate stages of reprogramming. Inhibiting signaling pathways associated with competing neuronal fate enhanced reprogramming efficiency. In conclusion, this study identifies early/intermediate reprogramming events and associated pathways that could be targeted to improve reprogramming efficiency.

## Highlights

• Developed an inducible model for reprogramming to hematopoietic progenitor cells
• Induction of SCL and LMO2 leads to conflicting and competing fate decisions
• Inhibiting pathway associated with neuronal fate enhances reprogramming efficiency
• SCL/LMO2-induced reprogramming occurs via a hemogenic endothelial stage

## Graphical abstract

**Figure:**
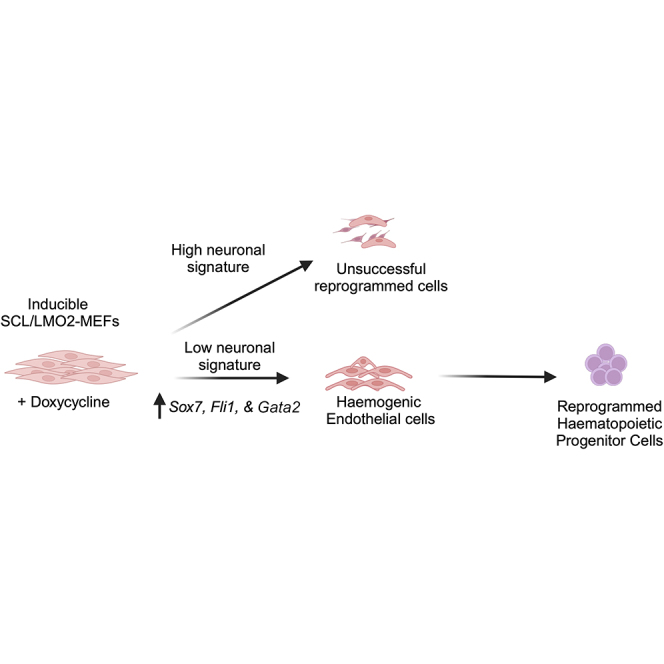

## Teaser

Batta and colleagues, using an inducible model of reprogramming fibroblasts to hematopoietic progenitor cells, showed that induction of SCL and LMO2 in fibroblasts induces conflicting neuronal and hematopoietic cell fates. Inhibiting neuronal fate improved reprogramming efficiency to hematopoietic progenitor cells. Furthermore, successful reprogramming to hematopoietic progenitor cells occurs via hemogenic endothelial cells.

## Introduction

Lineage-specifying transcription factors (TFs), when overexpressed in distant somatic cell types, can reconfigure chromatin organization and gene expression programs to induce cell fate change (Wang et al., 2021). The forced expression of TFs has enabled the generation of a variety of cell types with translational potential; however, further in-depth characterization of reprogramming events and reprogrammed cells is needed to support their use in the clinic. One major limitation of direct reprogramming methodologies is that only a minority of the starting cell populations successfully reprogram, suggesting mechanisms impeding cell fate transition. Several reports, aimed at direct reprogramming to different cell types, suggest that epigenetic and transcriptional heterogeneity, and cell cycle status, could contribute to the stochasticity of reprogramming (Zhou et al., 2016, 2019).

Transfusions of blood cells are used in the clinic to treat hematological cancers and other genetic disorders (Gratwohl et al., 2015). Lack of available donors and robust methods to expand hematopoietic stem cells (HSCs) prompted researchers to look for alternate methods such as directed differentiation of induced pluripotent stem cells (iPSCs). However, protocols that attempted directed differentiation to functional HSCs have been met with limited success (Zheng et al., 2023). An alternative safer approach to directed differentiation is direct reprogramming of somatic cells, which bypasses the need for an iPSC intermediate. Several groups have reprogrammed distinct somatic cell types to hematopoietic stem and progenitor cells (HSPCs) (Batta et al., 2014; Pereira et al., 2013; Riddell et al., 2014; Sandler et al., 2014). Of note, Lis et al. showed that endothelial cells could be reprogrammed to HSC-like cells (Lis et al., 2017). However, unlike fibroblasts, it is challenging to purify and expand endothelial cells in sufficient numbers to induce direct reprogramming.

Our group has shown that overexpression of five TFs (GATA2, ERG, LMO2, SCL, and RUNX1c) can reprogram mouse embryonic fibroblasts (MEFs) to induced HSPCs (iHSPCs) (Batta et al., 2014). Importantly, we have identified that, out of five TFs, SCL and LMO2 alone were sufficient to induce HSPC phenotype (Goode et al., 2016). Inclusion of the additional two TFs, RUNX1 and BMI1, remarkably enhanced the generation of iHSPCs with long-term multilineage repopulating capacity (Cheng et al., 2016). SCL drives the specification of hemogenic endothelium (HE), and LMO2 acts as a scaffold for SCL (El Omari et al., 2013; Porcher et al., 2017). Interestingly, SCL orchestrates the divergence of hematopoietic phenotype in the mesoderm by actively binding to cardiac enhancers and repressing cardiogenesis (Van Handel et al., 2012).

The mechanism behind SCL and LMO2-mediated induction of hematopoietic cell fate in fibroblasts and whether these factors have any role in suppressing fibroblast identity is unknown. Comprehending the exact mechanisms underlying specific cell fate transitions would enable the refinement of existing protocols and potentially uncover novel approaches for more efficient reprogramming systems. However, the low efficiencies of reprogramming often conceal the underlying molecular mechanisms involved in efficient cell fate change. The advent of single-cell technologies measuring multiple phenotypes have enabled researchers to identify intricate pathways involved in reprogramming (Biddy et al., 2018).

Here, we have developed an inducible mouse model to express SCL and LMO2 in cell types of choice. MEFs from these mice can be reprogrammed to hematopoietic progenitor cells (HPCs) with multilineage potential. Through single-cell gene expression studies, we found gene signatures reflecting hematopoietic and neuronal fate at intermediate stages of reprogramming, suggesting conflicting cell fates induced by the exogenous TFs. Inhibiting neuronal lineage pathway significantly improved reprogramming efficiency.

## Results

### Direct reprogramming of MEFs to iHPCs by the ectopic expression of SCL and LMO2

To investigate how SCL and LMO2 together induce a hematopoietic phenotype in fibroblasts, we developed a stable inducible system where we can achieve consistent and homogeneous overexpression of genes of interest. To this end, we have generated an embryonic stem (ES) cell line carrying a *Scl-T2A-Lmo2-*IRES-GFP construct under an inducible promoter (Figure S1A) (Kyba et al., 2002). Addition of doxycycline to this cell line (ES-iSL) uniformly induced the expression of GFP and *Scl* & *Lmo2* (Figures S1B and S1C). Next, we tested the effect of SCL and LMO2 induction on hematopoietic specification from ES cells. FLK1+ve hemangioblasts, differentiated from ES-iSL line, were cultured either in the presence or absence of doxycycline, and cells were harvested at days 1–3 (Figure S1D). Induction of SCL and LMO2 promoted HE specification as seen by the increase in number of TIE2 and CD41 double-positive cells (Figure S1E). Indeed, clonogenic assays with day 3 doxycycline-treated hemangioblast cultures showed an increased in the number of all types of hematopoietic colonies indicating the positive effect of SCL and LMO2 induction on hematopoietic specification (Figure S1F). Together, these data confirm the functionality of the ES-iSL line, and therefore, these cells were injected into blastocysts to generate a stable mouse line with inducible expression of SCL and LMO2.

**Figure 1:**
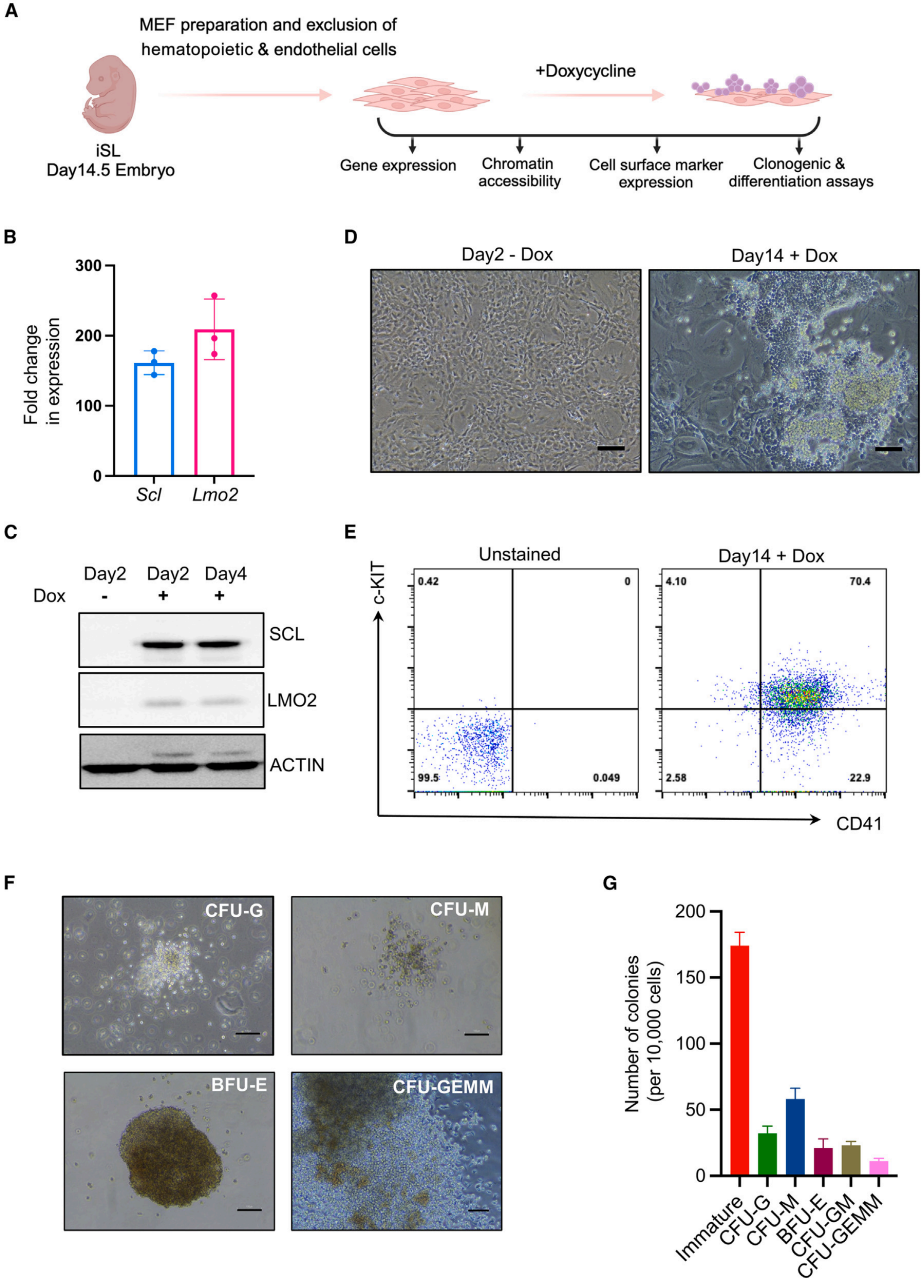
Ectopic expression of SCL and LMO2 induces reprogramming of fibroblasts to hematopoietic progenitor cells (A) Schematic representation of experimental methodology. Day 14.5 MEFs from iSL (inducible SCL and LMO2) mouse line were cultured in presence of doxycycline to induce *Scl* and *Lmo2* expression. Reprogramming cells were taken at different time points for molecular and functional characterization. (B) Fold change in the expression of *Scl* and *Lmo2* after 48 h of doxycycline treatment with respect to vehicle-treated MEFs (*N* = 3, MEFs isolated from three different embryos). (C) Representative western blot images showing SCL and LMO2 protein expression after 2 and 4 days of doxycycline treatment. Vehicle-treated MEFs at day 2 were used as a control (*N* = 3, MEFs isolated from three different embryos). (D) Representative bright-field images of vehicle-treated MEFs at day 2 and doxycycline (Dox)-treated MEFs at day 14. (E) Flow cytometry analysis of day 14 reprogrammed cells. (F) Representative bright-field images of the different types of colonies observed upon plating day 14 reprogrammed cells. CFU-G, granulocyte; CFU-M, macrophage; CFU-GM, granulocyte, monocyte; BFU-E, erythroid; CFU-GEMM, granulocyte, erythroid, monocyte, megakaryocyte. (G) Number of different types of colonies observed from 10,000 plated day 14 reprogrammed MEFs for 2 independent embryos, each performed in triplicates. Error bars represent SEM. Scale bars represent 100 μm. See also Figure S1.

To investigate the dynamics of reprogramming to HPCs, we decided to use MEFs as a starting population as they are easy to collect and expand. E14.5 MEFs were harvested from the inducible mouse line (iSL) and were depleted of contaminant hematopoietic and endothelial cells (Figure 1A). Expression of SCL and LMO2 was induced by treatment with doxycycline, and the fibroblasts were cultured in hematopoietic media to facilitate direct reprogramming. 48 h post induction, the cells exhibited much higher levels of *Scl* and *Lmo2* verified at RNA and protein levels (Figures 1B and 1C). Changes in cellular morphology could be observed in doxycycline-treated conditions as early as day 4–6; however, by day 8–14, clear cobblestone and suspension colonies of blood-like cells could be seen (Figure 1D). Reprogrammed cells expressed HSPC markers CD41 and c-KIT (Figure 1E). Colony-forming unit (CFU) assays revealed that induced HPCs (iHPCs) could generate macrophage, granulocyte, erythroid, and mixed lineage colonies, demonstrating their multilineage potential (Figures 1F and 1G). Together, these results show that MEFs harvested from the iSL mouse line can be successfully reprogrammed to iHPCs with multilineage clonogenic capacity.

### Characterization of SCL- and LMO2-induced HPCs

**Figure 2:**
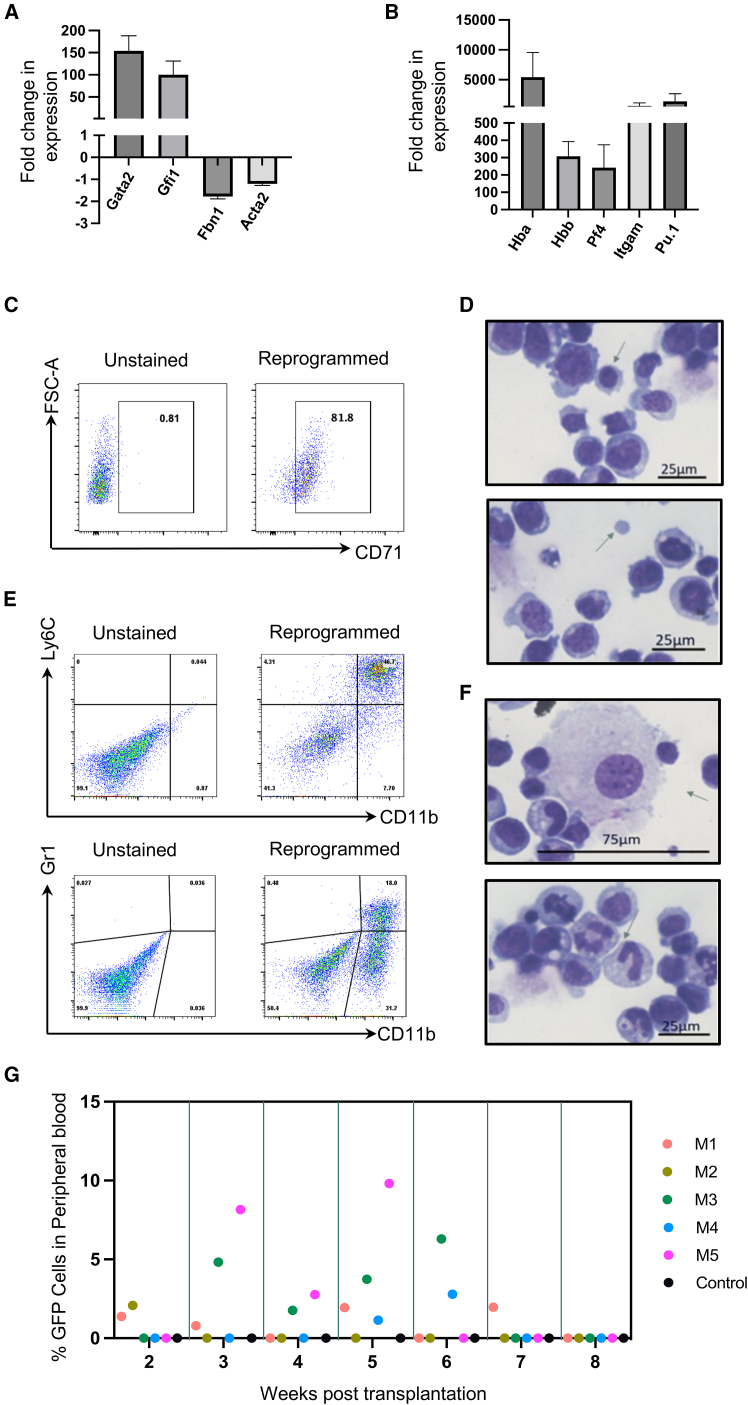
Reprogrammed hematopoietic progenitor cells have multilineage differentiation capacity (A and B) Fold change in expression levels of hematopoietic or fibroblast-specific (A) or mature lineage-specific (B) genes in day 14 reprogrammed cells as compared with day 2 control cells (*N*≥ 3, MEFs from three or more different embryos). (C and E) Flow cytometry analysis of reprogrammed cells measuring the expression of erythroid markers (C) or monocytic or granulocytic markers (E). (D and F) May Grunwald Giemsa staining of cells differentiated to erythroid (D) or myeloid lineages (F). (G) Percentage of GFP-positive cells within peripheral blood of recipient mice (5 mice labeled M1–M5) at indicated weeks post transplantation of reprogrammed cells. A mouse that did not receive any reprogrammed cells was used as a control. Scale bars represent 100 μm. Error bars represent SEM. See also Figure S2.

Next, we characterized day 14 reprogrammed cells by gene expression analysis and differentiation assays. As expected, we observed downregulation of the fibroblast-specific genes *Fbn1* and *Acta2* and upregulation of the HSPC-specific genes *Gata2* and *Gfi1* (Figure 2A). Erythroid, myeloid, and megakaryocytic lineage-specific genes such as *Hba* & *Hbb*, *ITGAM & Pu.1*, and *Pf4*, respectively, also showed increased expression (Figure 2B). To further validate the differentiation potential of iHPCs, we performed myeloid, erythroid, and lymphoid differentiation assays by culturing day 14 cells in permissive culture conditions. When cultured under conditions to promote erythroid specification, we observed cells positive for common erythroid cell surface marker, CD71, within 15 days (Figure 2C). Cytospin analysis of these cells further confirmed the presence of erythroblasts and enucleated erythrocytes (Figure 2D). In reprogrammed cultures, we observed CD11b/Ly6C-positive monocytes and CD11b/Gr1-positive granulocytes (Figure 2E). In addition, we also observed the morphology of various differentiated cell types such as monocytes, megakaryocytes, and neutrophils (Figure 2F). To promote lymphoid differentiation, the iHPCs were co-cultured with feeder cell lines OP9 and OP9-DLL1 to enable B and T cell differentiation, respectively. Co-culture of reprogrammed cells on OP9 stromal cells induced differentiation to B220-positive cells but CD19 was not detected above background levels (Figure S2A). When cultured on OP9-DLL1 stromal cells, reprogrammed cells differentiated to CD25 but never showed expression of CD3 (Figure S2B). Together, these data suggest differentiation capacity of iHPCs to multiple hematopoietic cell types.

To test the *in vivo* functionality of the reprogrammed cells, we first fluorescently labeled MEFs by transducing them with pSin-GFP. Reprogrammed c-KIT/GFP double-positive cells were sorted and injected into sub-lethally irradiated NSG mice (Figure S2C). Short-term engraftment was detected in the mice up to 7 weeks post injection (Figures 2G and S2D), as measured by the presence of GFP-positive cells within the peripheral blood. However, we did not see any engraftment in the bone marrow or blood when the mice were sacrificed at week 16. Together, our data showed that iHPCs can differentiate to multiple lineages *in vitro* and can engraft recipient mice *in vivo* short-term, suggesting that SCL and LMO2 together can induce an HPC phenotype in fibroblasts.

### SCL- and LMO2-induced HPC phenotype occurs via HE state

**Figure 3:**
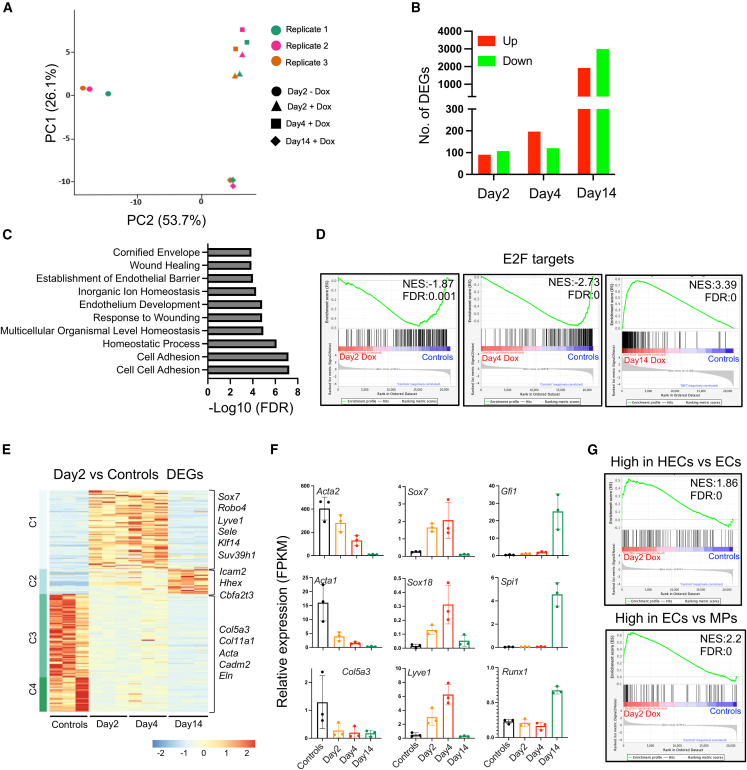
Reprogramming to hematopoietic progenitor cells occurs via hemogenic endothelial stage (A) Principal component analysis of transcriptome data performed on controls and day 2, day 4, and day 14 (c-KIT positive) reprogramming cells (*N* = 3, MEFs from three different embryos). (B) Bar chart showing the number of differentially expressed genes (DEGs) at day 2, 4, and 14 reprogramming cells as compared with control cells. (C) GO analysis of upregulated genes in day 4 reprogramming cells as compared with controls. (D) GSEA plots showing differential enrichment of E2F target genes in day 2, 4, and 14 reprogramming cells as compared with controls. (E) Heatmap showing the expression levels of DEGs in day 2 reprogramming cells as compared with control cells. (F) Relative expression levels of indicated DEGs in reprogramming and control cells. (G) GSEA plots showing enrichment of genes upregulated in hemogenic endothelial cells (HECs) compared to endothelial cells (ECs) (Solaimani Kartalaei et al., 2015) (top) and genes upregulated in ECs with respect to mesodermal progenitors (MPs) (Scialdone et al., 2016) in day 2 reprogramming cells as compared with controls. Error bars represent SEM. See also Figure S3.

To investigate global early and late molecular events involved in reprogramming, we performed transcriptome analysis on day 2 and day 4 doxycycline-treated cells, and c-KIT^+^-sorted day 14 reprogrammed cells, along with control day 2 vehicle-treated fibroblasts. Principal component analysis (PCA) showed that control and day 14 reprogrammed cells are transcriptionally very distinct from each other whereas intermediate day 2 and day 4 reprogrammed cells clustered closely (Figure 3A). We observed an increase in the number of differentially expressed genes (DEGs) as the reprogramming progressed, indicating a gradual change in identity as opposed to direct rapid transdifferentiation (Figure 3B; Tables S1, S2, and S3). The top 20 most significantly upregulated genes in the day 2 reprogrammed cells include the hematopoietic and endothelial TFs *Hhex* and *Sox7*, and neuronal marker gene *Tubb3* (Latremoliere et al., 2018). The increase in number of DEGs observed in day 4 compared with day 2 reprogrammed cells suggests a role for indirect targets of SCL and LMO2 in reprogramming. Possible indirect targets of SCL and LMO2 include mostly endothelial genes, e.g., *Acer2* and *CDH5*, and a few hematopoietic-specific genes, e.g., *Gata1* and *Gfi1* (Figure S3A). Gene Ontology (GO) analysis of upregulated genes in day 4 reprogrammed cells revealed pathways related to cell adhesion and endothelium development (Figure 3C). Downregulated genes in day 4 reprogrammed cells are linked to extracellular matrix and cell-cell junction signatures indicative of loss of fibroblast identity (Figure S3B). Gene set enrichment analysis (GSEA) comparing reprogrammed cells against controls showed negative enrichment of E2F targets in day 2 and day 4 reprogrammed cells but not in day 14 sorted HPCs, suggesting that initial reprogramming is negatively linked to active cell cycle status (Figure 3D).

We next performed clustering on DEGs to detect clusters of genes with different expression patterns during reprogramming. Clustering of day 2 vs. control DEGs revealed four main clusters (Figure 3E). Cluster 1 genes are transiently expressed in intermediate reprogrammed cells, and these include endothelial-specific genes such as *Sox7*, *Sox18*, and *Lyve1* (Figure 3F). Transient expression of these genes suggests that reprogramming of fibroblasts to HPCs occurs via an intermediate endothelial state (Figures 3E and 3F). A dramatic downregulation of fibroblast gene expression (clusters 3 and 4) in the day 4 reprogrammed cells suggests that reprogramming is initiated in most of the starting fibroblast populations (Figure 3F). Cluster 2 genes are gradually upregulated during reprogramming, suggesting their involvement in the hematopoietic specification. Clustering of day 4 vs. control DEGs further reiterated the enrichment of an endothelial gene signature in reprogramming intermediates (Figure S3C). Together, these data suggest that reprograming to HPCs is mimicking embryonic development. To further confirm this, we performed GSEA analysis investigating genes uniquely expressed in endothelial and HE cells (Scialdone et al., 2016; Solaimani Kartalaei et al., 2015). Results show that day 2 and day 4 reprogramming cells are positively enriched for genes overexpressed in HE cells compared to endothelial cells, and endothelial cells compared to mesodermal progenitor cells (Figures 3G and S3D). Clustering of day 14 DEGs compared to the control revealed 4 main clusters, two of which were composed of mainly hematopoietic genes (Figure S3E). In conclusion, transcriptome analysis at distinct stages of reprogramming suggests a downregulation of fibroblast identity followed by induction of an HE phenotype.

### Chromatin accessibility dynamics during early stages identifies downstream TF networks involved in reprogramming

**Figure 4:**
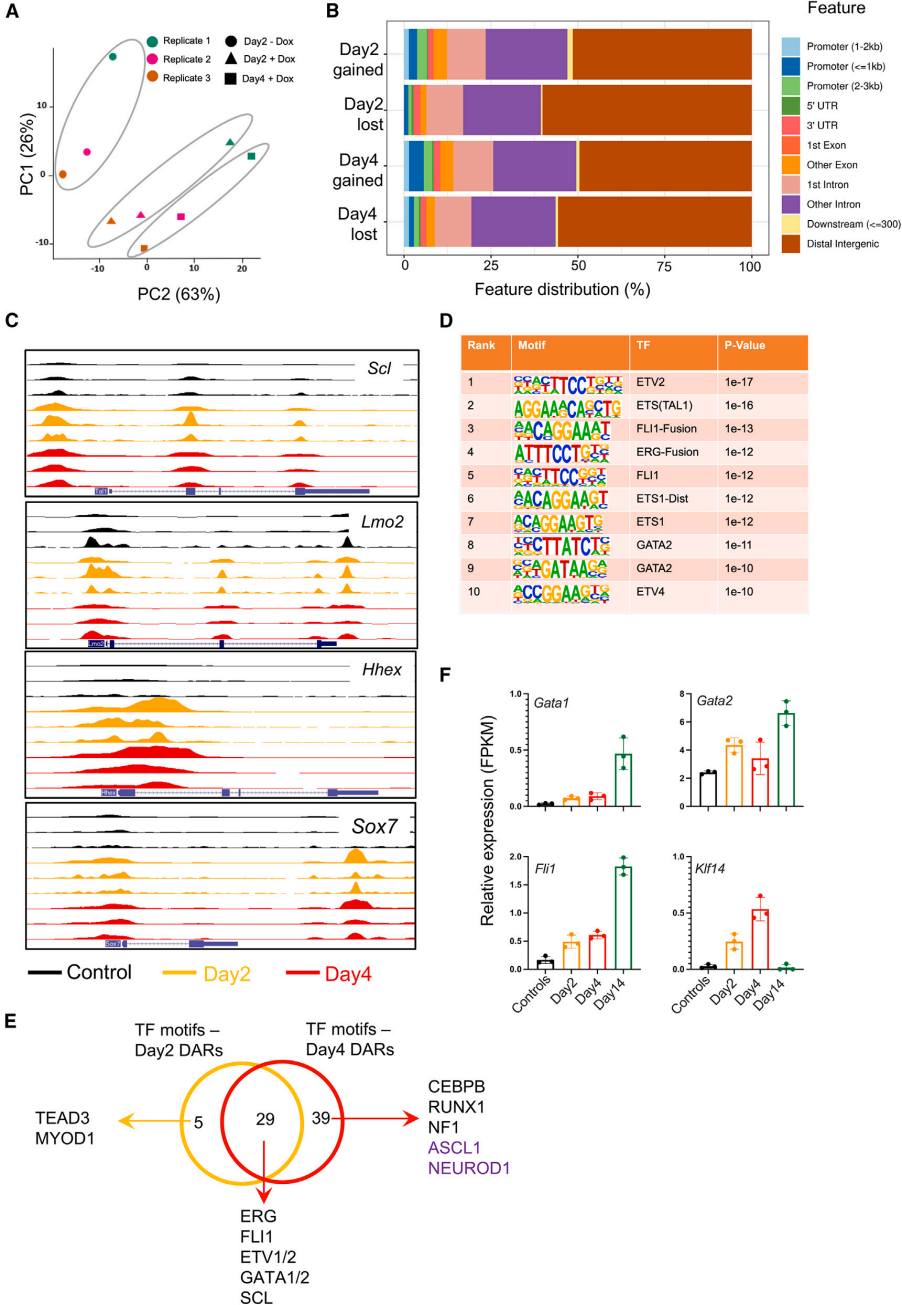
Gene regulatory networks involved in reprogramming to hematopoietic progenitor cells (A) Principal component analysis of ATAC-seq data performed on controls and day 2 and day 4 reprogramming cells (*N* = 3, MEFs from three different embryos). (B) Genomic annotation of differentially accessible regions (gained/lost) in day 2 and day 4 reprogramming cells as compared with controls. (C) UCSC browser plots depicting the chromatin accessibility at indicated gene loci in controls and day 2 and day 4 reprogramming cells. (D) Motif enrichment analysis in chromatin regions that gained accessibility in day 4 reprogramming cells as compared with controls. (E) Venn diagram showing an overlap of TF motifs that are enriched in promoter regions that are more accessible in day 2 and day 4 reprogramming cells. TFs related to neuronal fate are highlighted in purple. (F) Relative expression levels of indicated differentially expressed TF genes in reprogramming and control samples. Error bars represent SEM. See also Figure S4.

To investigate early changes in the chromatin accessibility landscape during reprogramming, we performed assay for transposase-accessible chromatin using sequencing (ATAC-seq) analysis on reprogramming cells collected at day 2 and day 4. Consistent with transcriptional changes, PCA analysis of early reprogramming cells showed substantial changes in chromatin accessibility (Figure 4A). We observed an increase in the number of differentially accessible regions (DARs) from day 2 to day 4 reflecting again dynamic transcriptional changes (Figures S4A; Tables S4 and S5). Majority of DARs mapped to distal elements (Figure 4B). Interestingly, both *Scl* and *Lmo2* endogenous gene loci gained accessibility in day 2/4 reprogrammed cells suggesting a positive feedback loop (Figure 4C). Other TF genes that gained accessibility in the early stages of reprogramming include *Hhex*, *Sox7*, *Gfi1*, *Gata2*, and *Cebpb*, suggesting their role in downstream reprogramming events (Figures 4C and S4B). We did not observe major changes in the accessibility of regions mapped to fibroblast genes *Acta2*, *Fbn1*, and *Col5a3* (Figure S4C). This suggests that while SCL and LMO2 activate hematopoietic phenotype by opening hematopoietic regulatory elements, they do not directly repress fibroblast gene expression.

Regions that gained accessibility in day 4 reprogramming cells showed enrichment for TF ETS (SCL), FLI1, GATA2, and ETV2 motifs (Figure 4D). Regions that lost accessibility are enriched for TF motifs belonging to the AP-1 family including AP-1, BATF, and FOSL2 but not ETS family, suggesting that loss of chromatin accessibility during reprogramming is not mediated by SCL and LMO2 (Figure S4D). In contrast to SCL’s known role in suppression of cardiac lineage (Van Handel et al., 2012), we did not observe any change in the accessibility or expression of cardiac TFs. However, SCL direct targets such as *Cbfa2t3*, *Hhex*, and *Scl* (endogenous) are upregulated in day 2 reprogramming cells with genomic regions linked to these genes opening upon induction with doxycycline (Figure S4E) (Wilson et al., 2009).

Although widespread chromatin changes were observed in day 2 and day 4 reprogramming cells, only a minority of the genes linked to DARs were differentially expressed (Figures S4E and S4F). However, we did observe a better overlap between genes linked to day 2 DARs with genes differentially expressed in day 4 reprogramming cells, suggesting that chromatin changes precede transcriptional alterations (Figure S4G). TFs whose motifs were commonly enriched in more accessible regions in day 2 and day 4 reprogrammed cells included ERG, GATA1/2, FLI1, and ETV1/2 (Figure 4E). Out of these TFs, GATA1, GATA2, FLI1, and KLF14 are overexpressed in the day 2 and day 4 reprogramming cells, suggesting their potential role in driving reprogramming (Figure 4F). Interestingly, we also observed enrichment of motifs linked to neuronal TFs such as ASCL1 and NEUROD1 in the day 4 reprogramming cells (Figure 4E). These data, together with dramatic upregulation of neuronal marker (*Tubb3*) gene expression, suggest a potential role for SCL and LMO2 in induction of neuronal program in fibroblasts. In conclusion, our data suggest that early reprogramming of fibroblasts to HPCs involves dynamic changes in chromatin configuration, predominantly mediated by SCL and LMO2 together, but later driven by the TFs GATA1/2 and FLI1.

### Investigating stochasticity of reprogramming to HPCs using scRNA sequencing

**Figure 5:**
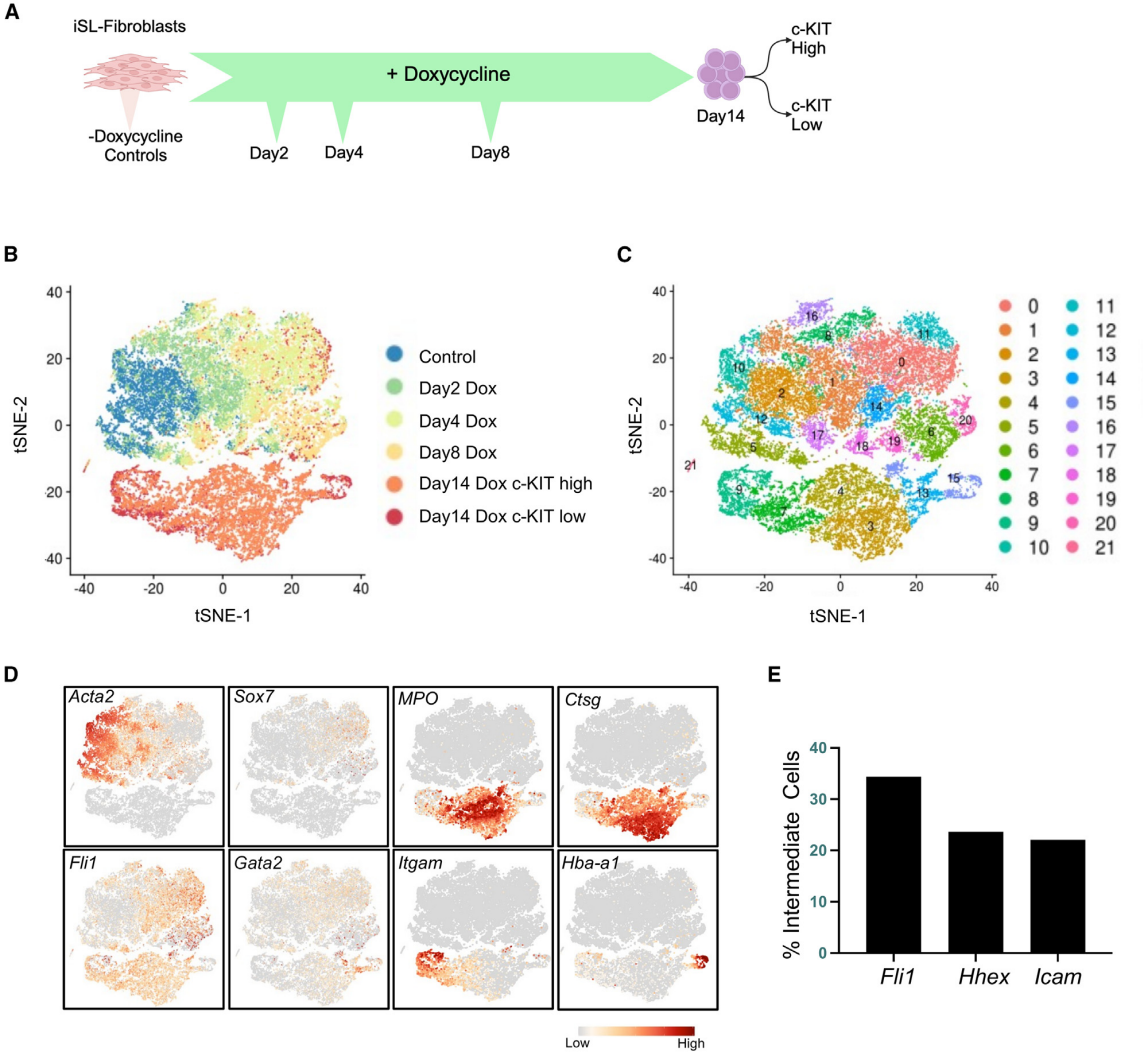
Single-cell transcriptome analysis of reprogramming intermediates (A) Schematic overview of single-cell RNA sequencing workflow. (B) t-distributed stochastic neighbor embedding (t-SNE) plot based on highly variable genes for all cells passing filtering thresholds, colored by sample type. (C) t-SNE plot displaying 22 transcriptionally distinct subpopulations as determined by unsupervised clustering. (D) Visualization of expression of indicated genes on t-SNE distribution. (E) Bar chart showing percentage of intermediate cells (days 2, 4, and 8) expressing indicated HE-specific genes. See also Figure S5.

Despite induction of SCL and LMO2 in most cells in our model system, only a minority of these MEFs undergo reprogramming (0.5%–5%), suggesting potential barriers (Figure S5A). While bulk cell RNA and ATAC-seq aided in identification of transcription networks and cellular landscapes at different stages of reprogramming, they did not explain why only a minority of cells undergo successful reprogramming. To decipher the heterogeneity of transcriptional responses that may contribute to the stochasticity of reprogramming, we performed single-cell RNA sequencing (scRNA-seq) at days 2, 4, 8, and 14 of reprogramming (Figures 5A and S5B). Day 14 reprogrammed cells showed different levels of c-KIT expression; therefore, we sorted for c-KIT high and c-KIT low populations (Figure S5B).

t-distributed stochastic neighbor embedding (t-SNE) analysis showed a clear separation of cells based on the time point they were harvested (Figure 5B). Seurat clustering highlighted 22 clusters, 6 of which were unique to day 14 reprogrammed cells, with the remaining composed of control and early/intermediate cells (Figures 5C and S5C). Control fibroblasts were composed of mainly 4 clusters, 3 of which are mostly unique to control cells, and cluster 5 was shared between control cells and day 2 reprogramming cells. Day 2 reprogramming cells were mostly distributed across clusters 1, 5, 8, 10, and 19. However, these clusters also included cells from later time points suggesting diverse kinetics of reprogramming (Figure S5C). The expression of the fibroblast genes *Acta2* and *Prrx1* showed a gradual downregulation from day 2 to day 8 doxycycline-treated cells when compared to control cells, which appeared consistent across most of the treated cells (Figures 5D and S5D). In addition, a significant downregulation of fibroblast gene expression at bulk and single-cell level without any evidence of cell death in fibroblasts suggests that the stochasticity of reprogramming is not mediated by failure to silence fibroblast gene expression (Figures S5E and S5F). Endothelial to hematopoietic transition (EHT) genes, such as *Cdh5*, *Sox7*, *Icam1*, and *Ctla2a*, showed upregulation from little to no expression in day 2 control cells, to increased expression by day 8 of doxycycline treatment, after which expression dropped in reprogrammed HPCs (Figures 5D and S5D). Quantification of cells that expressed EHT genes *Fli1*, *Hhex*, and *Icam* revealed that a large proportion (∼25%) of day 2, 4, and 8 reprogramming cells express these genes (Figure 5E). This suggests that inability to initiate a hemogenic program is not the main reason for poor reprogramming efficiency.

c-KIT high and c-KIT low day 14 cells did not cluster separately, although c-KIT low cells appeared to express mature blood cell markers *Itgam* and *Hba-a1* suggesting that c-KIT low cells are late progenitors (Figures 5B and 5D). We projected publicly available murine HSPC gene signatures onto our tSNE plots to characterize reprogramming/reprogrammed cells (Nestorowa et al., 2016). Most of the day 14 reprogrammed cells show similarity with megakaryocyte erythroid progenitors (MEPs), common myeloid progenitors (CMPs), and granulocyte-monocyte progenitors (GMPs) but not long-term (LT) or short-term (ST)-HSCs, consistent with their limited *in vivo* potential (Figure S5G). Interestingly, LT-HSC and ST-HSC gene signatures were enriched in early reprogramming cells, perhaps due to transcriptional similarity between HSCs and HE cells. Together, these analyses suggest that the stochasticity of reprogramming is not mainly caused by heterogeneity within the fibroblast population or inability to initiate reprogramming and that instead other factors during reprogramming affect successful transitions.

### Trajectory analysis on early and intermediate reprogrammed cells inferred multiple routes to successful reprogramming

**Figure 6:**
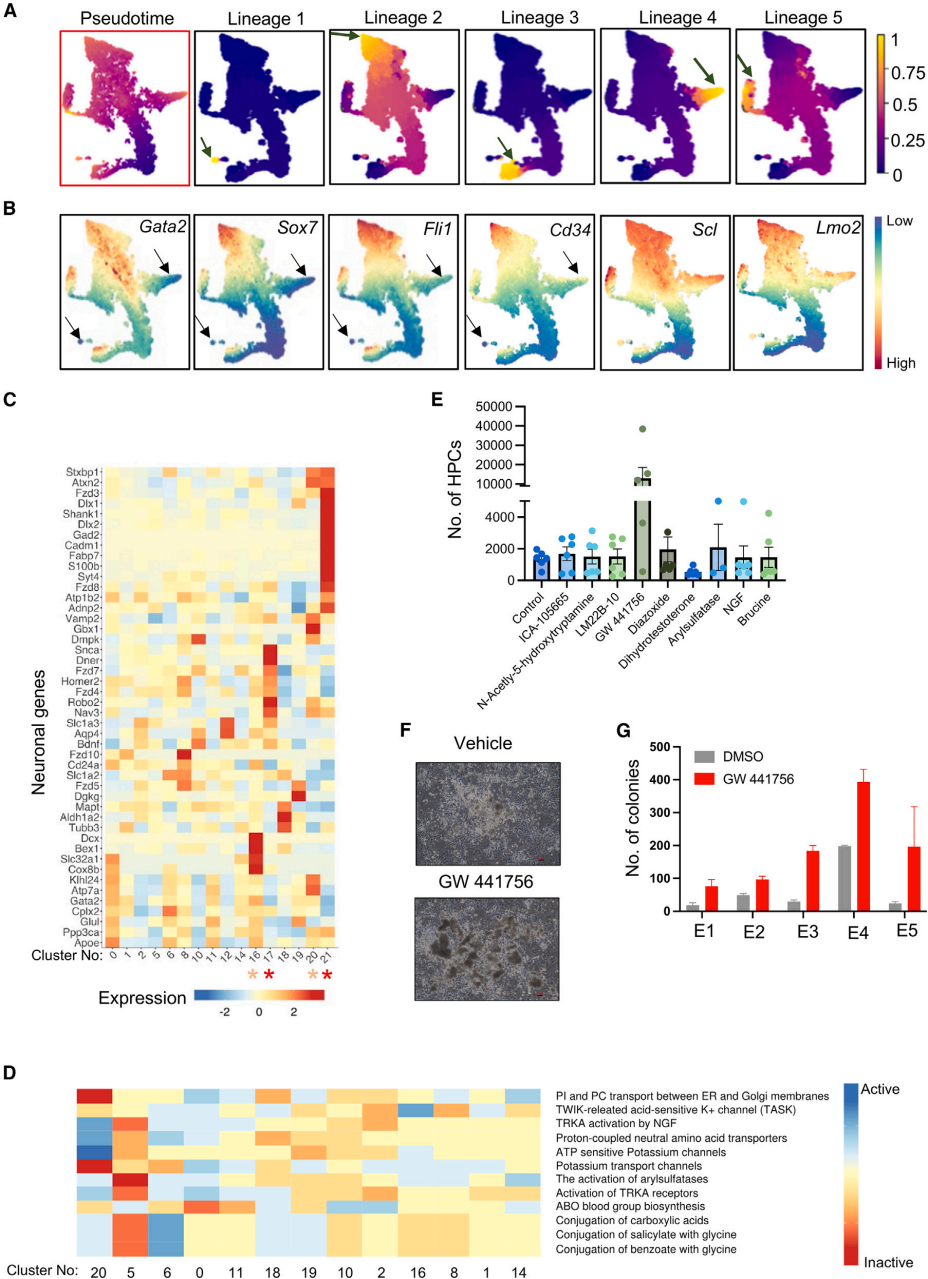
Reprogramming to hematopoietic progenitor cells occurs via multiple routes (A) Palantir trajectory analysis of day 2 control and day 2, 4, and 8 doxycycline-treated cells. Cells colored by pseudotime, with different lineage trajectories (lineages 1–5) depicted as different plots and endpoints highlighted by green arrows. In the color bar, 0 represents starting and 1 represents the ending of each lineage trajectory. (B) Visualization of expression of indicated genes on trajectory plots. Absence of some of their expression in lineages 1 and 4 are highlighted by black arrows. (C) Heatmap showing the expression of known neuronal genes among early and intermediate clusters of reprogramming cells. Clusters that expressed neuronal genes at high levels are marked with red stars (unsuccessful reprogrammed clusters) or orange stars (transition clusters). (D) Heatmap showing the activity of pathways in different intermediate and early reprogramming cell clusters. (E) Bar chart showing the number of HPCs within the wells of reprogramming cells treated with indicated small-molecule modulators. Each experiment was performed with 3 replicates using MEFs from one embryo. The data shown combined 2 separate experiments from two different embryos. (F) Representative bright-field images of hematopoietic colonies that were observed within wells treated with either vehicle or Trka inhibitor GW441756 after 14 days of reprogramming. (G) Number of hematopoietic colonies generated following reprogramming of MEFs treated with either vehicle control DMSO or Trka inhibitor GW 441756 (*N* = 5, MEFs from 5 different embryos E1–E5). Scale bars represent 100 μm. Error bars represent SEM. See also Figure S6.

Trajectory analysis of single-cell transcriptome data enables inference of cellular hierarchies (Setty et al., 2019). Therefore, to decipher transcriptional landscapes involved in successful reprogramming or lack thereof, palantir trajectory analysis was performed on the reprogramming cell population (Figure 6A). Our analysis treated control fibroblasts as the starting populations and excluded day 14 reprogrammed cells as they appeared very distinct and lacked continuity from other time points. Analysis showed that fibroblast populations take 5 trajectories (lineages 1–5) during reprogramming (Figures 6A and S6A). Lineage 1 was taken by very few cells with no observable expression of EHT genes and therefore was treated as an unsuccessful route to reprogramming (Figure 6B). Lineage 2 was the main lineage taken by most cells, which express high levels of EHT markers, and was considered as successful route to reprogramming (Figures 6A and 6B). Lineage 3, like lineage 1, showed early divergence and mainly included actively cycling cluster 5 cells (Figures 6A, S6B, and S6C). Initially, most control fibroblasts (∼75%) were in G1 phase of cell cycle (Figure S6D). Following induction of SCL and LMO2, a significant downregulation of active cell cycle genes was observed in day 2/4 reprogrammed cells (Figure 3D). As reprogramming progressed from day 2 to day 8, the proportion of cells in G2/M and S phase decreased (Figure S6D). These observations suggest that G1 phase, as opposed to G2/M and S phase, primes the active reprogramming process. However, lineage 3 cells also express EHT markers, indicating that actively cycling cells can also reprogram into HPCs (Figure 6B). This suggests the possibility that these cells may transition back to G1 phase prior to reprogramming. However, this hypothesis is speculative and lacks experimental evidence. The lack of induction of EHT genes but not *Scl* and *Lmo2* within lineage 4 suggested that this trajectory was not the one taken by cells undergoing successful reprogramming. EHT marker expression is also observed at the end of lineage 5 suggesting another successful route to reprogramming (Figure 6B). In summary, we observed that lineages 2, 3, and 5 represent successful routes to reprogramming whereas lineages 1 and 4 are unsuccessful routes.

Bulk cell transcriptome and ATAC-seq data suggested activation of neuronal networks in intermediate reprogramed cells. In our single-cell data, we have observed induction of neuronal genes such as *Robo2 Tubb3*, *Snca*, *Dner*, *Fzd7*, and *Fzd8* in intermediate reprogramming cells (Figure 6C). These genes are predominantly expressed in unsuccessful reprogramming clusters (lineage 1 – cluster 21 and lineage 4 – cluster 17) and clusters that are in transition (clusters 16 and 20) but not the endpoint successful reprogramming clusters (lineage 2 – cluster 11 and lineage 5 – cluster 18) (Figures 6C, S6E, and S6F). Genes uniquely expressed in unsuccessful reprogrammed cluster 21 are indeed related to several neuronal processes (Figure S6G). Given SCL’s role in neurogenesis (Bradley et al., 2006), we hypothesize that induction of neuronal gene expression in intermediate reprogramming cells may result in a conflicting state, which could impede reprogramming to HPCs.

To provide insights into signaling pathways active in cells undergoing reprogramming, pathway analysis on genes linked to early and intermediate reprogramming clusters was performed (Figure 6D). Interestingly, the cluster that showed the greatest difference in its pathway activity when compared to the rest included was cluster 20, with most cells in transition. We hypothesized that these pathways uniquely up- or downregulated in cluster 20 could play an important role in deciding cellular fate following SCL and LMO2 induction. The pathways that were differentially modulated in cluster 20 include tyrosine kinase receptor (Trka) activation by nerve growth factor (NGF), ATP-sensitive potassium channels, activation of aryl sulfatases, and glycine metabolism (Figure 6D) and therefore were selected for pathway modulation using small-molecule modulators (Figure S6H). To test the effects of targeting these pathways on reprogramming efficiency, MEFs were treated with chemicals from the initiation of reprogramming until day 14, after which the number of HPCs within each well was quantified.

Interestingly, of all the small molecules trialed for their effects on reprogramming, GW 441756, a potent and specific NGF/Trka inhibitor, showed a significant increase in the number of HPCs within the day 14 reprogrammed cultures (Figures 6E and 6F). NGF/Trka signaling plays an important role in neuronal development. Improved reprogramming efficiency by blocking the binding of NGF to Trka reinforces the likeliness of lineage divergence among the reprogramming cell population. To further confirm the positive effect of GW 441756 on reprogramming of fibroblasts rather than on proliferation of reprogrammed cells, we quantified the number of hematopoietic colonies at an early stage of reprogramming. We observed an increase in number of colonies at early stages of reprogramming suggesting improvement in reprogramming efficiency (Figure 6G). In conclusion, our analysis revealed that the conflicting cell fates induced by SCL and LMO2, in part, limit reprogramming to HPCs, and by inhibiting undesired cell fate induction, we could improve reprogramming efficiencies.

## Discussion

In this study, we developed a robust methodology to investigate the impact of ectopic expression of SCL and LMO2 in reprogramming somatic fibroblasts to iHPCs. We interrogated the transcriptional and *cis*-regulatory region landscape underlying the reprogramming and identified downstream drivers. Trajectory analysis revealed multiple routes to successful reprogramming. We found conflicting cellular fates induced by SCL and LMO2, which in part contributes to unsuccessful reprogramming.

We observed that fibroblast identity was uniformly and gradually erased across all cells, and many early reprogramming cells expressed EHT genes such as *Fli1*, *Hhex*, and *Icam*, suggesting that poor efficiency of reprogramming is not mediated by heterogeneity of the starting populations. This is consistent with observations made in reprogramming to cardiomyocytes where the identity of the starting fibroblast population did not impact the cell fate change induced by TFs (Liu et al., 2017). Several studies found that the proliferative state of the starting cell population negatively impacts reprogramming efficiency (Treutlein et al., 2016; Zhou et al., 2019). Our comprehensive analysis of both bulk and single-cell transcriptomes at intermediate stages of reprogramming demonstrated that majority of these cells are in G1 phase of the cell cycle. This observation suggests that either induction of SCL and LMO2 arrests cells in G1 phase or that the initiation of the reprogramming process requires cells to be in G1 phase. The gradual reduction in the frequency of cells in G2/M and S phase from day 2 to day 8 suggests support for the latter. This implies that the active phase of cell cycle is not permissive to transcriptional changes induced by transgene activation. However, once cells transition to an inactive phase of cell cycle, cell fate change can then be initiated. Our clustering analysis revealed multiple clusters of successfully reprogrammed cells with expression of EHT genes. These are clusters placed along multiple but not all palantir trajectories, suggesting that reprogramming to iHPCs can occur via multiple routes.

Several studies have revealed that reprogramming to hematopoietic fate occurs via an intermediate state called HE (Batta et al., 2014; Pereira et al., 2013). In this study, we also see transient upregulation of endothelial genes. The TFs specifying (e.g., SOX7, GFI1) HE fate are directly induced by SCL and LMO2 in the early stages of reprogramming. *In vivo* repopulation studies showed that reprogrammed cells only have short-term engraftment capacity, suggesting that HE cells generated through the reprogramming process are only capable of giving rise to multipotent progenitor (MPP) cells. Indeed, heterogeneity within HE cells exists with some cells capable of producing HSCs while others commit to MPPs (Dignum et al., 2021). Alternately, HE cells with HSC potential may have been generated; however, the culture conditions used in the study are not permissive to maintain HSCs. Besides HE fate, we have also observed expression of some neuronal genes in some clusters. This was also evident in bulk cell analysis of the day 2 reprogramming cells. The neuronal signatures observed in certain clusters could represent partially reprogrammed neuronal cells. Indeed, clusters with neuronal gene signature were represented in unsuccessful reprogramming trajectories. Upon blocking of the Trka activation by NGF, which is an important signaling pathway playing roles in the survival and differentiation of neurons (Levi-Montalcini, 1987; Thoenen and Edgar, 1985), the reprogramming efficiency to HPCs is increased, suggesting that conflicting cell fate decisions are induced by SCL and LMO2 together. Induction of neuronal fate in fibroblasts is not surprising given that *Scl* is expressed in the central nervous system and plays a role in neuronal differentiation (Achim et al., 2013; Elefanty et al., 1999).

TFs that initiate reprogramming while inducing a desired cell phenotype also actively downregulate gene expression of starting populations (Penalosa-Ruiz et al., 2020). SCL is also shown to maintain hematopoietic phenotype during development by actively repressing other mesodermal lineages (Chagraoui et al., 2018; Van Handel et al., 2012). We did not observe any changes in cardiac genes known to be repressed by SCL either at chromatin levels or at gene expression levels. We also did not observe any changes in the chromatin accessibility of fibroblast-specific genes during the early stages of reprogramming. This suggests that SCL is not directly repressing fibroblast identity; rather, it is directly inducing hematopoietic phenotype.

The TFs that induce direct reprogramming are thought to act as pioneer factors (Zaret and Carroll, 2011). Basic helix-loop-helix (bHLH) proteins can gain stable access to nucleosomes upon interaction with other adaptor proteins (Soufi et al., 2015). bHLH TF SCL interacts with non-DNA-binding factor LMO2, and this interaction is essential for SCL’s DNA binding activity (Stanulovic et al., 2017). In our study, chromatin accessibility in early reprogramming cells showed that several regions that are completely inaccessible in control cells became accessible. These regions are predicted to be recognized by SCL, with the bHLH motif being the top scorer, confirming SCL’s direct role in switching chromatin configuration. Indeed, SCL’s known direct target genes, which were inaccessible in control cells, became open in early reprogramming cells. Together, these observations suggest a pioneering activity for SCL in reprogramming to iHPCs.

In conclusion, our findings indicate that SCL/LMO2-induced reprogramming of fibroblasts into HPCs occurs via an intermediate HE state. We have identified TF networks that play a crucial role in the reprogramming process. The relatively low efficiency of reprogramming is not a consequence of cell-intrinsic features of the starting fibroblast population or a failure of exogenous TFs to initiate the reprogramming process. Instead, a barrier to fibroblast reprogramming into HPCs is the conflicting cellular identities imposed by SCL and LMO2. Blocking the undesired lineage commitment improved reprogramming efficiency to HPCs.

## Methods

### Direct reprogramming of iSL MEFs to HPCs

Hematopoietic and endothelial cell-depleted E14.5 MEFs were seeded onto 0.1% gelatin-coated plates. The following day, the media was changed to hematopoietic media (1× Iscove’s modified Dulbecco’s medium, 10% FBS [Gibco], 10% protein-free hybridoma medium [Gibco], 1% L-glutamine, 1% penicillin-streptomycin, 25 μg/mL ascorbic acid, 4.5 × 10^−4^ M MTG, 180 μg/mL transferrin [R&D Systems], 50 ng/mL SCF [PeproTech], 25 ng/mL IL-3 [PeproTech], 25 ng IL-6 [PeproTech], 1% GM-CSF and 1% TPO conditioned media, 2,000 U/mL EPO, 10 ng/mL M-CSF, and 100 ng/mL Flt3 ligand [PeproTech]), and cells were treated with 1 μg/mL of doxycycline. Media was half changed every 4 days.

### *In vivo* engraftment assay

MEFs were transduced with pSin-GFP lentivirus in the presence of 8 μg/mL polybrene (Sigma). 72 h post transduction, MEFs were directly reprogrammed to HPCs and sorted for c-Kit+GFP+ cells at day 14. The sorted cells were cultured in hematopoietic media for 3 days to facilitate expansion prior to injection (intrafemoral) into sublethally (2 Gy) irradiated NSG mice. Engraftment was monitored by GFP expression detected in peripheral blood. This *in vivo* experiment was performed according to protocols of the Institutional Animal Care and Use Committee at A^∗^STAR.

### RNA sequencing

MEFs were seeded onto 0.1% gelatin-coated T25 cm^2^ flasks, 24 h prior to initiation of reprogramming with doxycycline. Cells were harvested on days 2, 4, and 14 of reprogramming, with day 14 cells sorted for c-KIT-positive cells. Library preparation was performed based on poly(A) selection using the SureSelect polyA kit (Agilent). The samples were sequenced on the NextSeq 500 (Illumina) generating 60 bp paired-end reads.

### ATAC-seq assay

MEFs were seeded onto 0.1% gelatin-coated plates, and the following day, were treated with 1 μg/mL doxycycline for 48 and 96 h. 50,000 cells of each treated and control cells were centrifuged at 350 x g for 5 min at 4°C and washed in cold PBS. ATAC-seq experiment was performed as described previously (Buenrostro et al., 2015).

### scRNA-seq

Reprogramming cells were harvested at days 2, 4, 8, and 14. On each day of sample harvesting, the cells were trypsinized, counted, and frozen in 10% DMSO containing FBS and stored in liquid nitrogen until library preparation steps. Libraries were prepared using the Chromium Next GEM Single cell 3′ Reagents Kit v.3.1 (10× Genomics) according to manufacturer’s instructions. Libraries were sequenced using the NovaSeq 6000 platform on an S2 flow cell. sc-small interfering RNA-seq data were processed as preciously described (Gautam et al., 2021).

## Resource availability

### Lead contact

Further information and requests for resources and reagents should be directed to and will be fulfilled by the lead contact, Kiran Batta (kiran.batta@manchester.ac.uk).

### Material availability

ES line and mouse line generated in this study will be made available on request.

### Data and code availability

Sequencing data were deposited into the Gene Expression Omnibus database under accession number GSE287568.

## Acknowledgments

The study was primarily funded by the University of Manchester and A^∗^STAR Institute Singapore joint PhD program awarded to S.S. Epigenetics of Haematopoiesis group is funded by The Oglesby Charitable Trust. The Stem Cell Biology group is funded by CRUK Manchester Institute Core grants (no. C5759 and A27412). Y.-H.L. is supported by the NRF Investigatorship award NRFI2018-02; IAF-PP grant H1801a0021, NRF2019-THE002-0001, and NRF000407-00; NMRC grant OFIRG21nov-0088; A∗STAR grants W22W3D0007 and C211318012; and A∗STAR BMRC Use-Inspired Basic Research award. The Developmental Haematopoiesis Group is supported by the 10.13039/501100000265Medical Research Council (MR/P000673/1; MR/T000384/1) and the 10.13039/501100000268Biotechnology and Biological Sciences Research Council (BB/R007209/1). F.M.R.A. is supported by 10.13039/501100000289CRUK grant numbers C5759/A20971 and C5759/A27412. K.H. is supported by the NMRC grant MOH-000937-00 and A∗STAR grant C210812003. L.A.G. is supported by 10.13039/501100000289CRUK grant numbers C5759/A27445 and C147/A25254. We thank CRUK-MI core facilities including molecular biology core facilities and flow cytometry facilities. All illustrations in the manuscript were created with BioRender.com.

## Author contributions

K.B., G.L., and Y.-H.L. conceived and designed the study. S.S., L.A.G., A.H.A., and K.B. performed all the experiments, analyzed the data, and wrote the manuscript. K.H. and F.M.R.A. performed computational analysis. V.K. and D.H.W. provided critical feedback on the manuscript.

## Declaration of interests

The authors declare no competing interests.

## Supplemental information

Document S1. Figures S1–S6

Table S1. Differentially expressed genes in day 2 reprogramming cells vs. controls (FDR < 0.05), related to Figure 3

Table S2. Differentially expressed genes in day 4 reprogramming cells vs. controls (FDR < 0.05), related to Figure 3

Table S3. Differentially expressed genes in day 14 c-KIT-positive reprogrammed cells vs. controls (FDR < 0.05), related to Figure 3

Table S4. Differentially accessible regions in day 2 reprogramming cells vs. controls (FDR < 0.05), related to Figure 4

Table S5. Differentially accessible regions in day 4 reprogramming cells vs. controls (FDR < 0.05), related to Figure 4

Document S2. Article plus supplemental information
