# Supplementary material for: Competing dynamic gene regulatory networks involved in fibroblast reprogramming to hematopoietic progenitor cells
Source: Stem Cell Reports. 2025 Apr 3;20(5):102473. doi: 10.1016/j.stemcr.2025.102473 (PMC12143154; doi:10.1016/j.stemcr.2025.102473)
Supplement: Document S1. Figures S1–S6 [file mmc1.pdf]

Stem Cell Reports, Volume *20*

Supplemental Information

Competing dynamic gene regulatory networks involved in fibroblast re-programming to hematopoietic progenitor cells

Samiyah Shafiq, Kiyofumi Hamashima, Laura A. Guest, Ali H. Al-anbaki, Fabio M.R. Amaral, Daniel H. Wiseman, Valerie Kouskoff, Georges Lacaud, Yuin-Han Loh, and Kiran Batta

Document S1

Competing dynamic gene regulatory networks involved in fibroblast reprogramming to haematopoietic progenitor cells

Samiyah Shafiq, Kiyofumi Hamashima, Laura A Guest, Ali H Al-anbaki, Fabio M R Amaral, Daniel H Wiseman, Valerie Kouskoff, Georges Lacaud, Yuin-Han Loh, and Kiran Batta

Supplementary Figures:

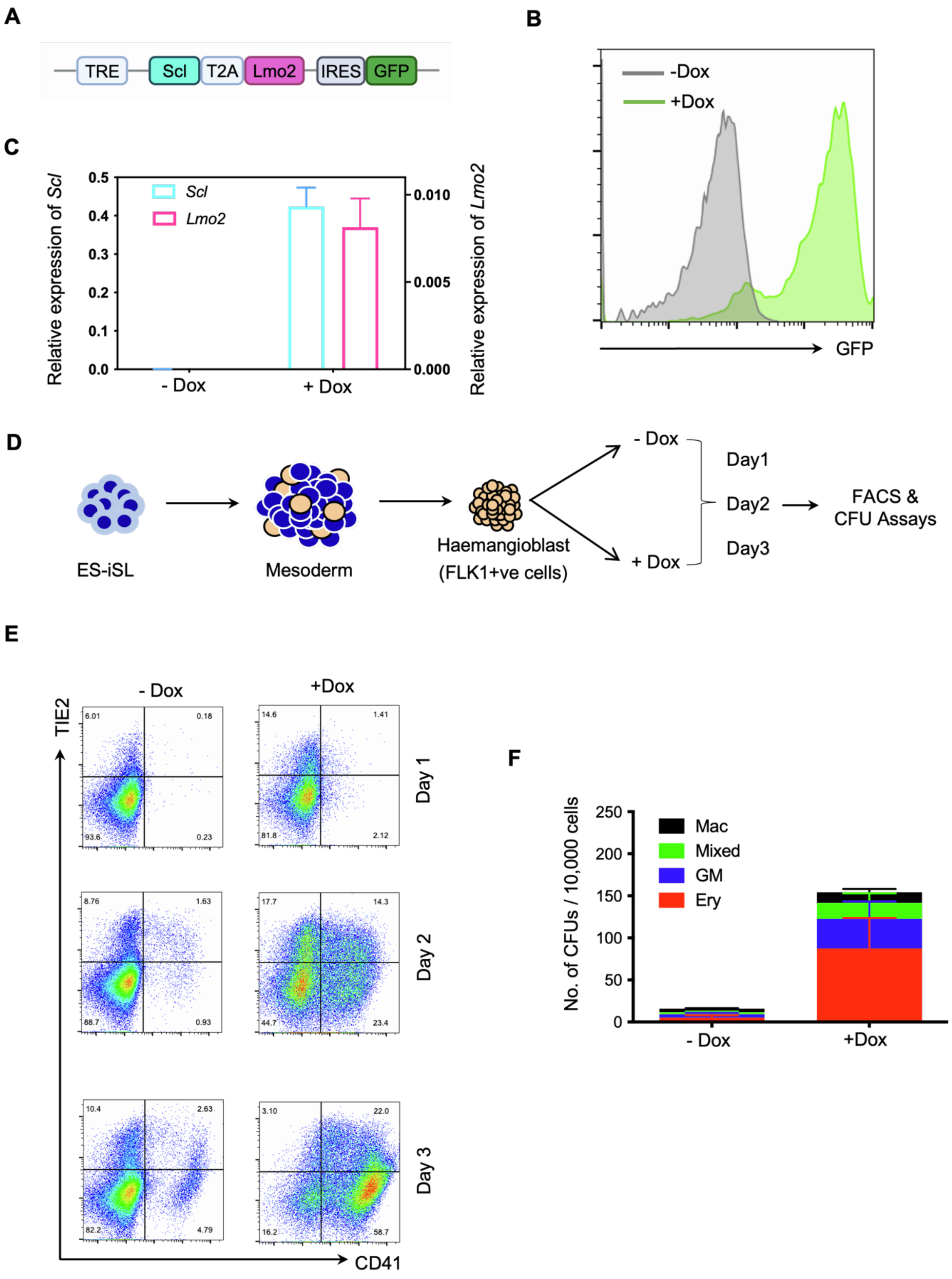

Figure S1

Figure S1: A. Schematic of the cassette incorporated into murine ES cells to generate inducible *Scl* and *Lmo2* ES line (iSL). TRE: Tetracycline response element **B.** Flow cytometry analysis measuring GFP expression of iSL ES line treated with or without doxycycline (Dox) for 24 hours. **C.** Relative expression of *Scl* and *Lmo2* in ES-iSL line treated with or without doxycycline for 24 hours with respect to housekeeping gene β-actin. Data is shown for a representative iSL-ES line performed in three technical replicates. **D**. Schematic representation of experimental methodology used to investigate the impact of *Scl* and *Lmo2* induction on differentiation of haemangioblasts to haematopoietic stem and progenitor cells. CFU: Colony forming unit **E**. Flow cytometry analysis of FLK1+ve haemangioblasts treated with or without doxycycline for 24, 48 and 72 hours**. F**. Number of different types of colonies observed from 10,000 plated cells taken at 24, 48 and 72 hours following with or without doxycycline induction in haemangioblast cultures from ES-iSL line. Data is shown from one experiment performed with three technical replicates. Mac: Macrophage, GM: Granulo-monocytic, Ery: erythroid.

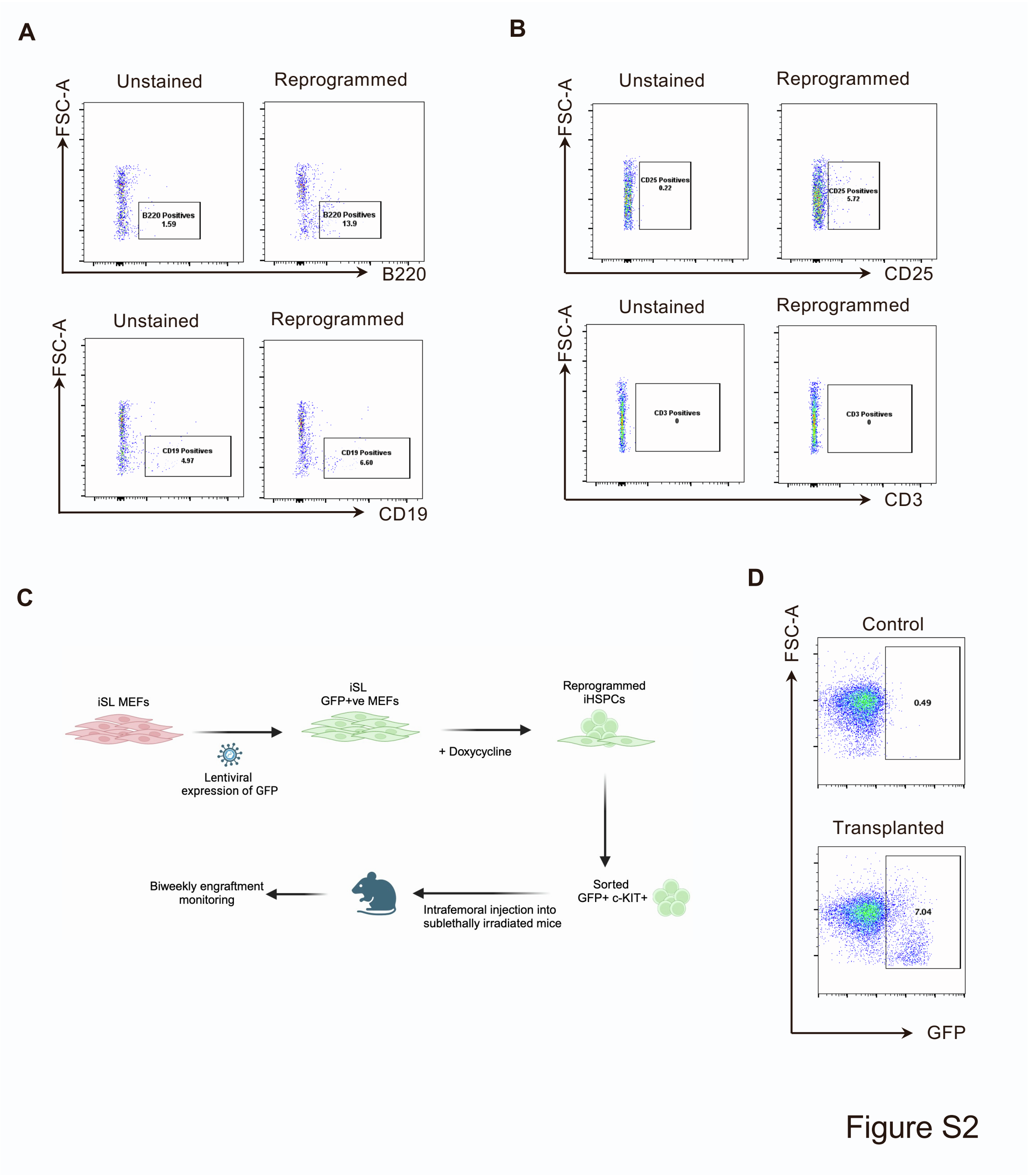

Figure S2: A. Flow cytometry analysis of day 14 reprogrammed cells when differentiated under culture conditions to promote B cell differentiation (day 15). **B.** Flow cytometry analysis of day 14 reprogrammed cells when differentiated under culture condition to promote T cell differentiation (day 15). **C.** Schematic for the in vivo engraftment study. Day 14.5 iSL-MEFs were transduced with GFP expressing lentivirus and the transduced MEFs were cultured in presence of doxycycline to initiate reprogramming. Reprogrammed cells were sorted for c-KIT and GFP expression and the double positive cells were expanded and injected intrafemorally into sub lethally irradiated mice (N=5). Blood samples were taken every two weeks to measure the levels of engraftment based on GFP positivity. **D.** Flow cytometry analysis of peripheral blood taken at week 5 from a representative mouse transplanted with reprogrammed cells and a control mouse injected with PBS.

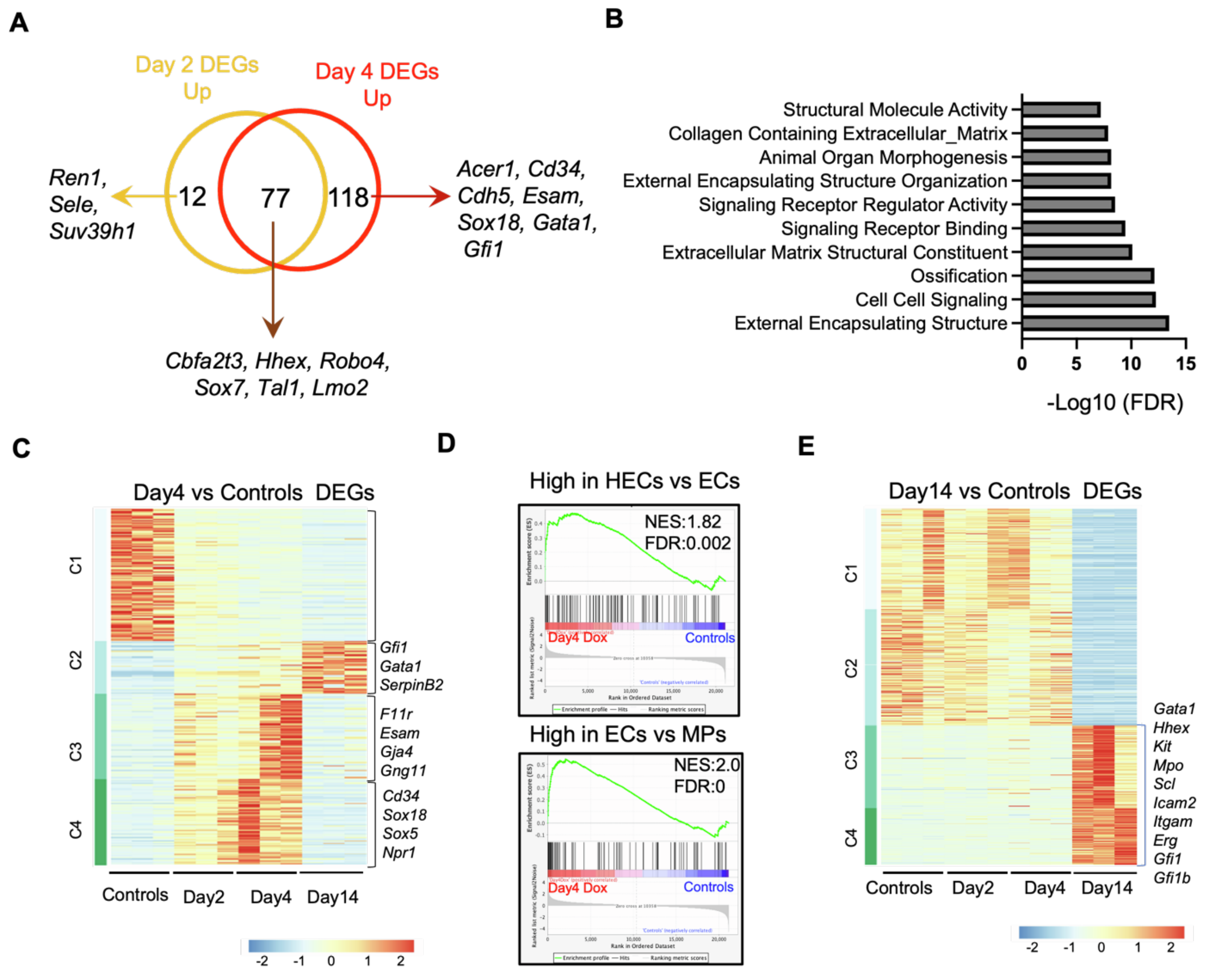

Figure S3

Figure S3: A. Venn diagram showing an overlap of genes that are upregulated in day 2 reprogramming cells with upregulated genes in day 4 reprogramming cells as compared with controls. **B.** GO analysis of downregulated genes in day 4 reprogramming cells as compared with controls. **C.** Heatmap showing the expression levels of DEGs in day 4 reprogramming cells as compared with control cells. Unsupervised K-means clustering identified 4 clusters (C1-C4). **D.** GSEA plots showing enrichment of genes upregulated in haemogenic endothelial cells (HECs) compared to endothelial cells (ECs) (Solaimani Kartalaei et al., 2015) (top) and genes upregulated in ECs with respect to mesodermal progenitor cells (MPs) (Scialdone et al., 2016) (bottom) in day4 reprogramming cells as compared with controls. **E.** Heatmap showing the expression levels of DEGs in day 14 c-KIT positive reprogramming cells as compared with control cells. Unsupervised K-means clustering identified 4 clusters (C1-C4).

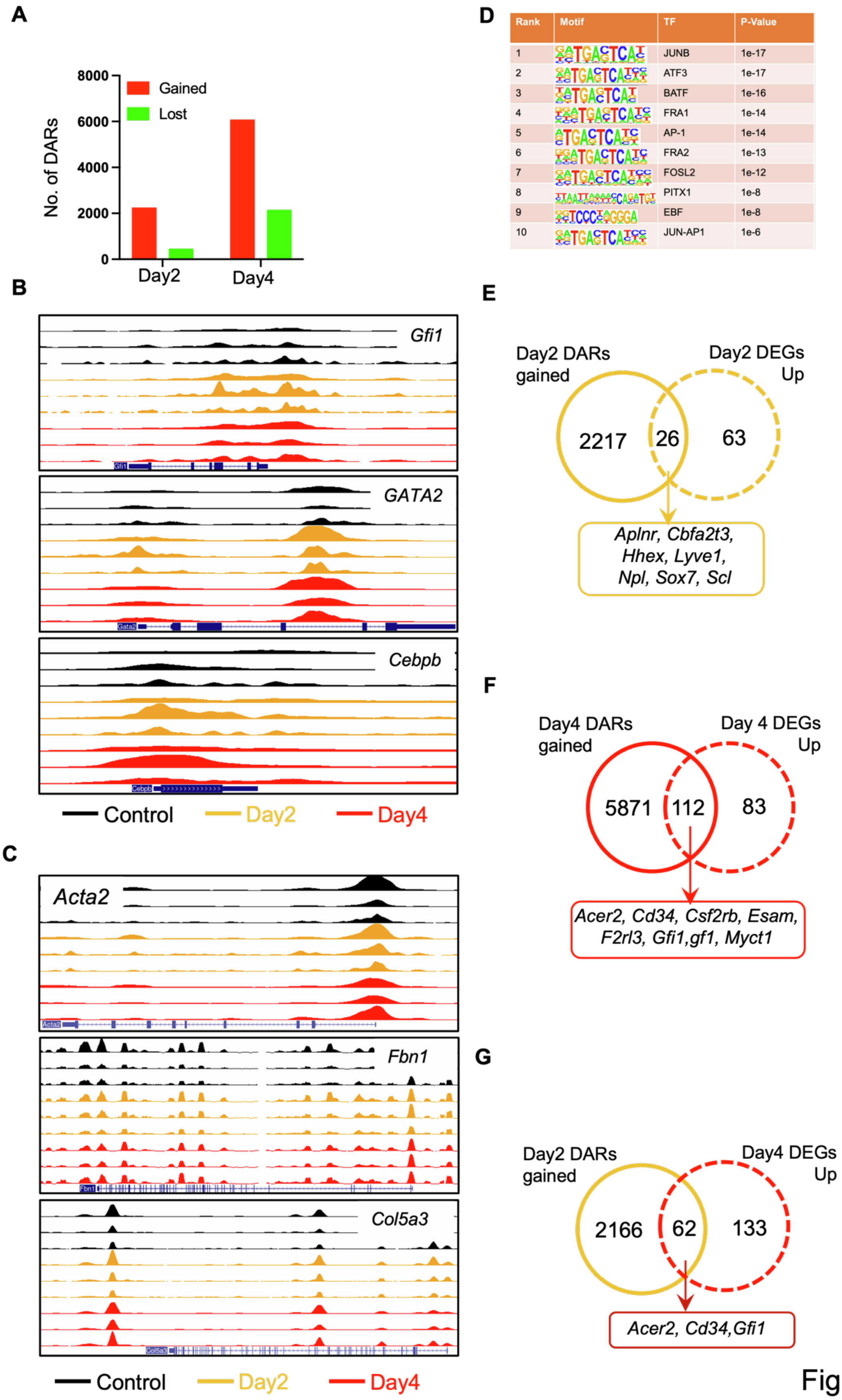
A
No. of DARs
8000
6000
4000
2000
0
Gained
Lost
Day2
Day4
B
Gfi1
GATA2
Cebpb
Control
Day2
Day4
C
Acta2
Fbn1
Col5a3
Control
Day2
Day4
D
Rank
Motif
TF
P-Value
1 JUNB 1e-17
2 ATF3 1e-17
3 BATF 1e-16
4 FRA1 1e-14
5 AP-1 1e-14
6 FRA2 1e-13
7 FOSL2 1e-12
8 PITX1 1e-8
9 EBF 1e-8
10 JUN-AP1 1e-6
E
Day2 DARs gained
Day2 DEGs Up
2217
26
63
Aplnr, Cbfa2t3, Hhex, Lyve1, Npl, Sox7, Scl
F
Day4 DARs gained
Day 4 DEGs Up
5871
112
83
Acer2, Cd34, Csf2rb, Esam, F2rl3, Gfi1,gf1, Myct1
G
Day2 DARs gained
Day4 DEGs Up
2166
62
133
Acer2, Cd34,Gfi1

Figure S4

Figure S4: A. Bar chart showing the number of differentially accessible regions (DARs) at day 2, and day 4 reprogramming cells as compared with control cells. **B & C.** UCSC browser plots depicting the chromatin accessibility at indicated haematopoietic (B) and fibroblast (C) genes in control, day 2 and day 4 reprogramming cells. **D.** Motif enrichment analysis in chromatin regions that lost accessibility in day 4 reprogramming cells as compared with controls. **E & F.** Venn diagram showing an overlap of genes that are mapped to regions gained accessibility in day 2 (E) and day 4 (F) reprogramming cells with upregulated genes in day 2 and day 4 reprogramming cells respectively**. G.** Venn diagram showing an overlap of genes that are mapped to regions that gained accessibility in day 2 reprogramming cells with upregulated genes in day 4 reprogramming cells.

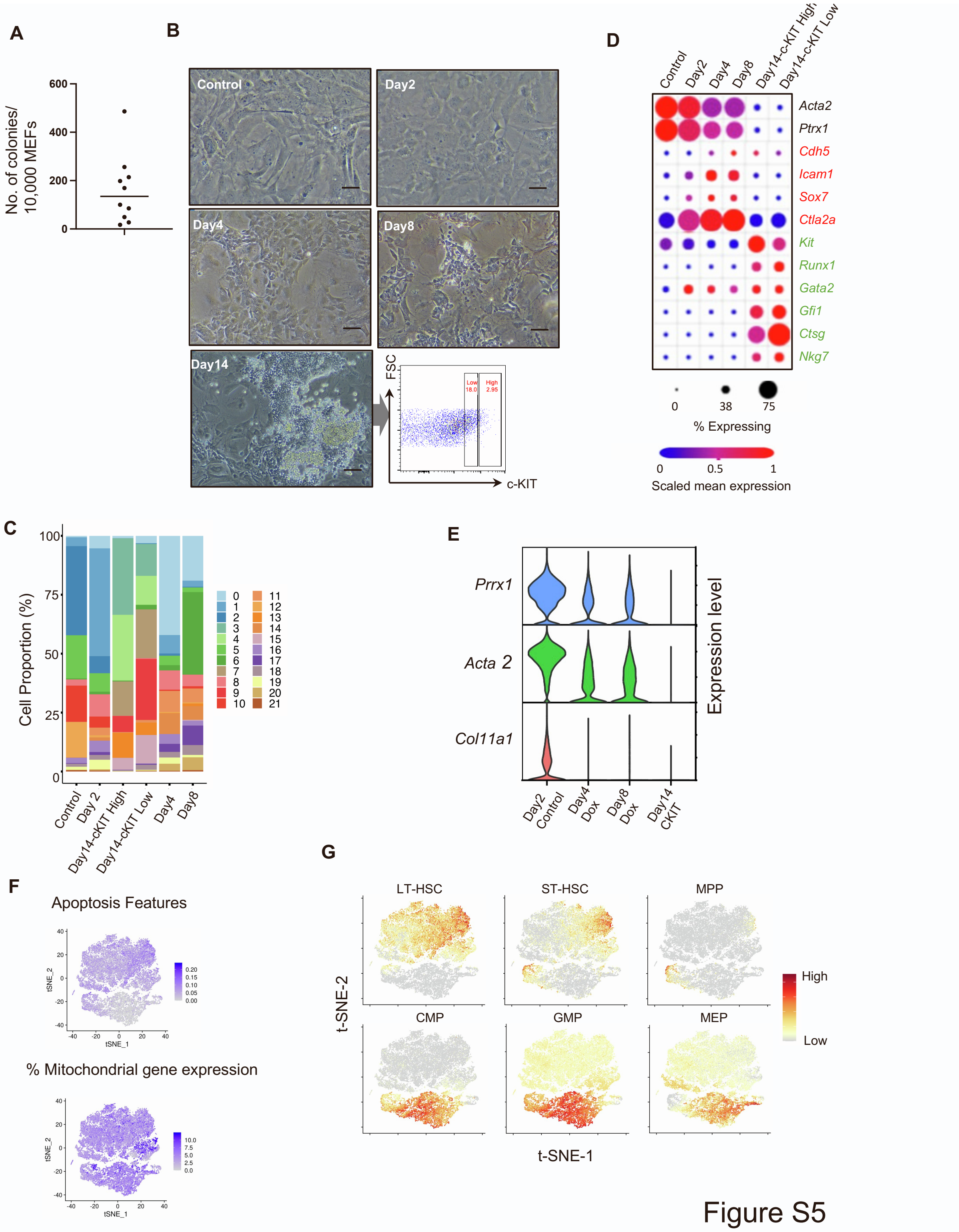

A
No. of colonies/ 10,000 MEFs
600
400
200
0
B
Control
Day2
Day4
Day8
Day14
FSC
Low
High
2.95
c-KIT
C
Cell Proportion (%)
100
75
50
25
0
Control
Day 2
Day14-cKIT High
Day14-cKIT Low
Day4
Day8
D
Control
Day2
Day4
Day8
Day14-c-KIT High
Day14-c-KIT Low
Acta2
Ptrx1
Cdh5
Icam1
Sox7
Ctla2a
Kit
Runx1
Gata2
Gfi1
Ctsg
Nkg7
0
38
75
% Expressing
0
0.5
1
Scaled mean expression
E
Prrx1
Acta 2
Col11a1
Expression level
Day2 Control
Day4 Dox
Day8 Dox
Day14 CKIT
F
Apoptosis Features
% Mitochondrial gene expression
tSNE_1
tSNE_2
G
LT-HSC
ST-HSC
MPP
CMP
GMP
MEP
High
Low
t-SNE-1
t-SNE-2

Figure S5

Figure S5. A. Number of haematopoietic colonies observed, following induction of SCL and LMO2, by 10,000 MEFs (N=10, MEFs from 10 different embryos). **B.** Brightfield images showing morphology of cells at day 2 control, day 2, 4, 8 and 14 doxycycline treated timepoints. Day 14 cells were sorted for the c-KIT positive (low and high) populations prior to harvesting for single cell library preparation. Scalebar 100 μm. **C.** Distribution of different clusters (identified by Seurat) in samples collected at different stages of reprogramming. **D.** Bubble plot showing the expression levels of indicated genes across time points during reprogramming. **E.** Violin plot showing the expression levels of indicated genes across time points during reprogramming **F.** Visualization of expression of apoptosis gene expression signature (top panel) or % mitochondrial gene expression (bottom panel) on t-SNE distribution. **G.** Visualization of expression of indicated HSPC specific gene expression signature (Nestorowa et al., 2016) on t-SNE distribution.

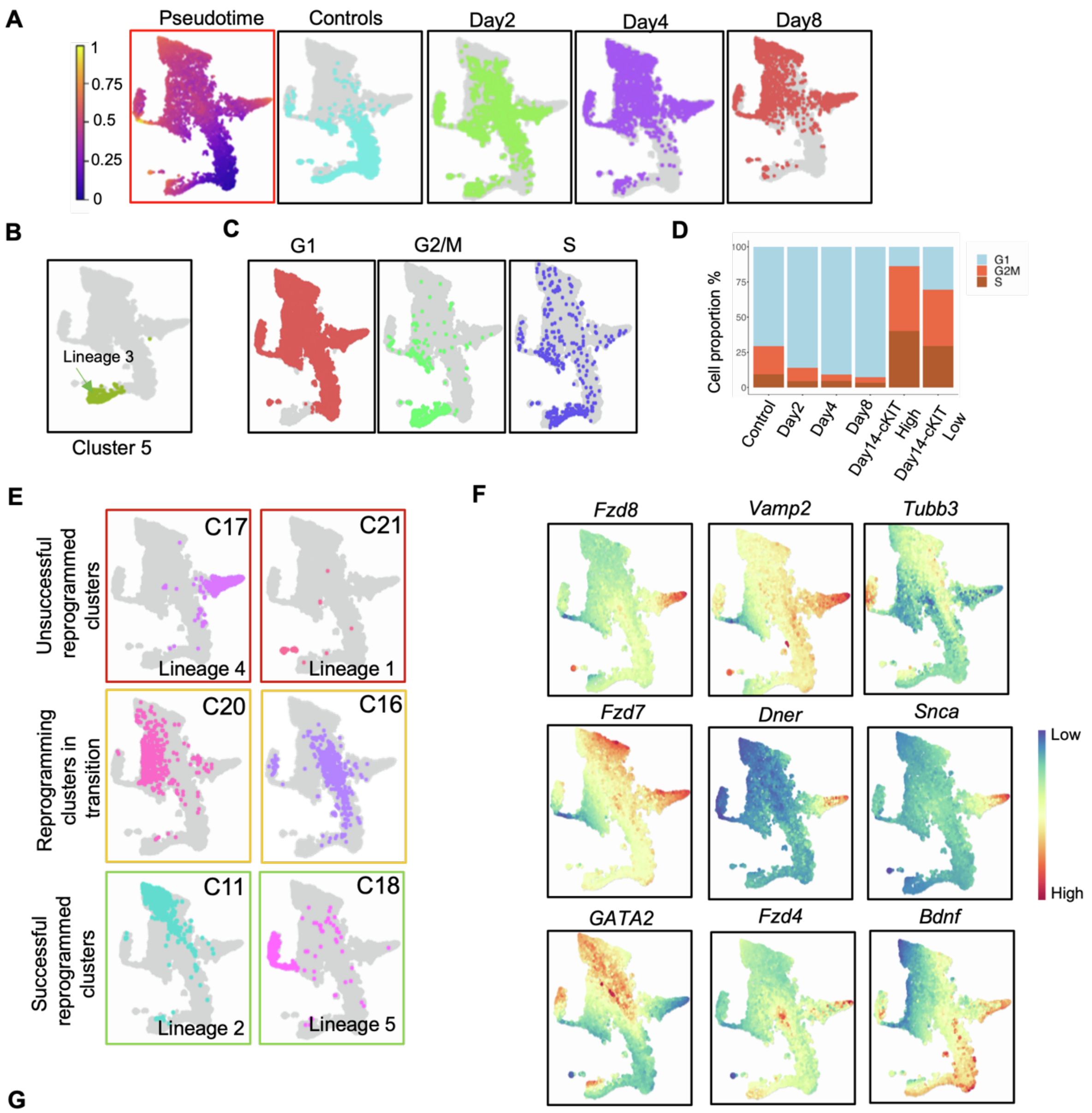

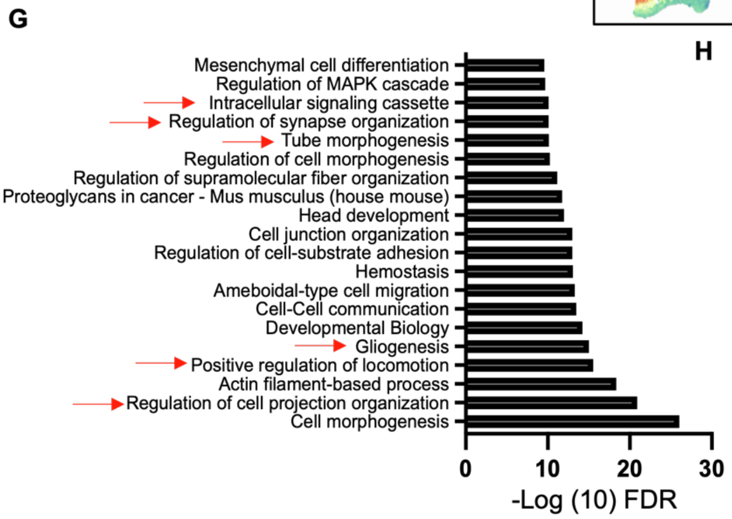

H

Chemical	Action
ICA-105665	K channel Opener
N-Acetly-5-hydroxytryptamine	Trkb Activator
LM22B-10	Trkb/c Activator
Diazoxide	ATP sensitive K channel activator
Arylsulfatase	Arylsulfatase activator
NGF	Trka activator
Brucine	Glycine blocker
GW 441756	Trka Inhibitor
Dihydrotestoterone	Arylsulfase Blocker

Figure S6

Figure S6: A. Cells within the Palantir trajectory plot coloured by sample identity. **B.** Cells in cluster 5 are highlighted in green within the trajectory. Lineage 3 is identified by a green arrow. **C.** Visualization of different cell cycle stage gene signatures overlaid within the trajectory. **D.** Breakdown of sample types by proportion of cells within each phase of cell cycle. **E.** Cells in unsuccessfully reprogrammed clusters (C17 and C21), transition clusters (C20 and C16) and successfully reprogrammed clusters (C11 and C18) are highlighted within the trajectory. **F.** Visualization of expression levels of different neuronal genes overlaid on the trajectory**. G.** Pathway analysis of genes uniquely expressed in Cluster 21 vs rest of the clusters. **H.** Small molecules modulators, together with their mode of action, screened to measure their impact on reprogramming efficiency are listed in the table.

Supplementary Tables:

Table S1: Differentially expressed genes in day 2 reprogramming cells vs controls (FDR <0.05). Related to Figure 3.

Table S2: Differentially expressed genes in day 4 reprogramming cells vs controls (FDR <0.05). Related to Figure 3.

Table S3: Differentially expressed genes in day 14 c-KIT positive reprogrammed cells vs controls (FDR <0.05). Related to Figure 3.

Table S4: Differentially accessible regions in day 2 reprogramming cells vs controls (FDR <0.05). Related to Figure 4.

Table S5: Differentially accessible regions in day 4 reprogramming cells vs controls (FDR <0.05). Related to Figure 4.

Supplementary Experimental Procedures:

iSL ES line generation: cDNA encoding SCL-T2A-LMO2 was cloned into modified KYBA vector p2LOX-IRES-GFP. This vector was used to generate iSL-ES line as described previously (Kyba et al., 2002).

ES cell culture and differentiation: Murine iSL-ES cell line was maintained and differentiated as described previously (Sroczynska et al., 2009). FLK1 positive haemangioblasts were sorted and cultured in presence or absence of doxycycline in medium containing 1X IMDM, 10% FBS, 0.5 mM Ascorbic Acid, 4.5×10^{-4}M MTG, 2 mM L-glutamine, 80 μg/ml transferrin and 50 μg/ml penicillin-streptomycin. Haemangioblast cultures were harvested and analysed for cell surface marker expression and clonogenic potential.

iSL mouse line generation: iSL-ES line was injected into blastocyst of pseudo pregnant mice and pups with highest percentage of chimerism were backcrossed with C57BL/6 to generate iSL mouse line. Genotyping was done to verify the presence of transgenic loci. Animal work was carried out as per Home Office Legislation under the Animal Scientific procedures Act (ASPA) 1986 and was approved by the Animal Welfare and Ethics Review Body (AWERB) of the Cancer Research UK Manchester Institute.

MEF isolation from iSL mouse line: MEFs were isolated by dissecting E14.5 embryos under a stereomicroscope. The head and internal organs were discarded, and the remaining tissues of each individual embryo were washed in sterile phosphate-buffered saline (PBS). Subsequently, the tissues were placed in a well of a 6-well plate and finely minced into a slurry using a scalpel. To facilitate homogenisation, 2 ml of trypsin-EDTA (Gibco) solution was added, and the mixture was incubated for 5 minutes at 37°C. Repeated pipetting was performed to aid in the homogenisation process. The resulting cell suspension was transferred to a T175 cm2 flask containing 30 ml of Iscove's Modified Dulbecco's Medium (IMDM) (Gibco) supplemented with 20% fetal bovine serum (FBS, Gibco), 1% penicillin/streptomycin (P/S, Invitrogen), 1% L-Glutamine (L-Glut, Invitrogen) and 20 µm of alpha-monothioglycerol (MTG, Sigma), hereafter referred to as MEF media. The cells were cultured at 37°C at 5% O2 overnight, and the next day, the media was changed to remove any unattached material. The remaining adherent cells were cultured until they reached confluency and then frozen in 10% dimethyl sulfoxide (DMSO, Sigma) in FBS.

Depletion of haematopoietic and endothelial cells from MEF cultures: Before conducting direct reprogramming experiments, the MEFs underwent a depletion process to remove haematopoietic and endothelial contaminant cells. This was achieved through magnetically activated cell sorting (MACS). The cells were subjected to a 20-minute staining process on ice, using a master mix of biotin-conjugated antibodies specific to CD45, c-KIT, CD41, and CD34 (Biolegend) in PBS supplemented with 5% FBS and 0.5 mM Ethylenediaminetetraacetic acid (EDTA, Sigma) (referred to as FACS buffer from here onwards). Two washes were performed by adding 2 ml of MACS buffer (0.5% BSA and 2mM EDTA in PBS) and centrifuging at 350 g for 5 mins at 4°C each time. Subsequently, the cells were resuspended in 80 µl of MACS buffer, 20 µl of anti-biotin microbeads (Miltenyi Biotec) were added, and cells were incubated for an additional 20 mins on ice. The cells were washed again twice as before and resuspended in 500 µl of MACS buffer after the second spin. In parallel, the MACS® MS separation column (Miltenyi Biotec) was pre-prepared by fitting it onto the OctoMACS™ Separator system and rinsing the empty column with 500 µl of MACS buffer. The cell suspension was then applied to the column after which the column was washed 3 times with 500 µl of MACS buffer to further remove any unlabelled cells. The flow through containing the fibroblasts was collected and cells bound to the column (haematopoietic and endothelial contaminants) were

flushed by adding 1 ml of MACS buffer to the column and inserting the plunger to collect the labelled cells. The cells were washed in PBS and a minimum of 30,000 of unsorted, flow through and eluate cells were collected for flow cytometry analysis to confirm the purity of the fibroblast population before being utilized in downstream experiments.

Flow cytometry and cell sorting: Surface marker expression was analysed using the BD LSR II (BD biosciences) or the NovoCyte Penteon Flow Cytometer (Agilent). To prepare the cells for analysis a minimum of 40,000 cells were washed in FACS buffer. Pelleted cells were resuspended in antibodies that were diluted in FACS buffer according to the manufacturer's instructions for each antibody. Cells were stained with different combinations of the following antibodies: CD45-PerCp Cy5.5 (1:100, Miltenyi), CD41-Pecy7 (1:20, Miltenyi), c-KIT-APC (1:100, Miltenyi), CD11b (1:100, Miltenyi), CD71 (1:100, Miltenyi), for 25 minutes on ice. After staining the cells were washed twice in FACS buffer and resuspended in 350 µl of FACS buffer. To aid the flow cytometry analysis and compensation, single stained controls were also prepared using cells for GFP and DAPI single stained controls and UltraComp beads™ for all the remaining markers. For GFP controls, day 2 dox treated cells were harvested. The beads were stained on ice for 20 minutes after which they were washed in 1 ml FACS buffer and resuspended in 350 µl of FACS buffer. Flow cytometry was then performed on the BD LSR II or NovoCyte Penteon Flow Cytometer and surface marker expression was analysed on the FlowJo software.

Cell sorting was performed primarily on the Aria Fusion. To sort the cells, they were stained as described and analysed. The cells were mostly sorted for the c-KIT$^+$ population after successful reprogramming; live, single, c-KIT positive cells were gated and sorted into new FACS tubes for downstream use.

Western blot: MEFs were cultured for 48 hours in the presence of 1 µg/ml doxycycline, washed twice with cold PBS and incubated for 30 minutes at 4°C with continuous mixing in RIPA Buffer (150 mM NaCl, 1% Nonidet P-40, 0.1% SDS, 25 mM Tris (pH 7.4) and 1% sodium deoxycholate) to lyse the cells. The cell lysate mixture was centrifuged for 20 minutes at 12000 rpm at 4°C, and the supernatant containing the lysate was collected. The protein extract was quantified using the Pierce™ BCA Protein Assay Kit (Thermo Scientific) and 10-100ug of protein used NuPAGE™ LDS sample buffer (Invitrogen) was added to the lysates of each sample and incubated at 70°C for 10 minutes to denature the proteins. The whole protein extracts were loaded onto a NuPAGE™ 4 to 12%, Bis-Tris, 1.0-1.5 mm, protein gel (Invitrogen) and separated using the NuPAGE™ SDS-PAGE Gel System (Invitrogen) in pre-cooled MOPS buffer (1M MOPS, 1M Tris-Base, 2% SDS, 20.5mM EDTA). The gel was mounted onto a nitrocellulose membrane and sandwiched into a transfer cassette which was run at 100 V for 60 minutes at 4°C. After the transfer was complete the

membrane was stained with Ponceau stain (Sigma) to confirm the successful transfer. Blocking of the membrane was performed in 5% milk for 3 hours at room temperature (RT) after which primary antibody was added at a 1:1000 dilution and the membranes were incubated overnight at 4°C. The following morning the membranes were washed in PBS with 0.1% tween (PBS-T) 3 times for 10 minutes each. Subsequently, secondary antibody incubation was performed for 1 hour at RT. The membranes were washed again 3 times in PBS-T for 10 minutes each and tapped dry with a paper towel. Pierce™ ECL Western Blotting Substrate (Thermo Scientific) at a 1:1 ratio was poured onto the membranes and allowed to incubate for 1-2 minutes after which the ChemiDoc imagine system (Bio-Rad) was used to visualise the bands.

Quantitative RT-PCR: RNA was extracted from cells using the Monarch Total RNA Miniprep Kit (NEB) according to kit instructions. Up to 500 ng of RNA was then reverse transcribed using the iScript™ cDNA Synthesis Kit (BioRad). For the qRT-PCR reaction, 2 ng of cDNA was added to a master mix containing 5 µl BlitzAmp Hotstart qPCR Master Mix (MiRXES), 0.2 µl of 5 µM qPCR primers and nuclease free water (Invitrogen). Each reaction was performed in a total volume of 10 µl with three technical replicates per sample. For normalisation purposes β -Actin or GAPDH were always included as house-keeping genes and internal no template control. qRT-PCR results were analysed using the Delta-delta Ct method ($2^{-\Delta\Delta Ct}$).

CFU assay: Colony forming assay was performed on directly reprogrammed HPCs by resuspending 10,000 cells in 100 µl IMDM media supplemented with 2% FBS. The cells were then added to 900 µl of MethoCult ™ (STEMCELL Technologies) and supplemented with 25 ng IL-3, 25 ng IL-6 and 50 ng SCF. A 16G needle and 1 ml syringe were then used to dispense 300 µl of the cell suspension into each well of a 24-well tissue culture treated plate in triplicate. PBS was added to the surrounding wells to prevent the methylcellulose from drying out. Colonies were counted and scored on day 14 based on standard morphological criteria.

Morphological Staining: Approximately 10,000 cells were spun onto a microscope slide using the Thermo Cytospin 4 Cyto centrifuge (Thermo). The slides were left to airdry which was followed by fixation in methanol for 5-10 mins at room temperature. Slides were submerged in May Grunwald stain (Sigma) freshly diluted with equal volumes of Sorenson's buffer (1x KH2PO4, 1x Na2HPO4 in ddH2O) for 15-20 minutes. Immediately after, Giemsa stain (Sigma) was diluted in 1:10 in Sorenson's buffer and used to stain the slides for 30 minutes. Following washes in running tap water and Sorenson's buffer, the slides were left to air dry and mounted using DPX neutral mounting medium (Sigma).

Intrafemoral injections: To perform intrafemoral injection of reprogrammed cells, the mice were kept anaesthetized using isoflurane whilst cells were injected into the femoral cavity. Before injection the area of injection was disinfected with 70% ethanol and betadine surgical scrub. To stabilise the joint for injection the knee was flexed to a 90-degree angle and a small incision was made using a scalpel to increase visibility of the bone. Next, a 26-gauge insulin needle loaded with a maximum of 200,000 cells was inserted into the joint surface of the femur through the patellar tendon and introduced into the bone marrow cavity where the cells were then injected. The skin was then sutured with 4-0 Sofsilk™ sutures (Medtronic) and the mice were observed closely for the first week post-surgery during which time painkillers were administered for 3 days and antibiotics for 5 days to aid recovery and prevent infection.

Assay for Transposase-accessible chromatin with sequencing (ATAC-Seq): MEFs were seeded onto 0.1% gelatin coated plates and the following day were treated with 1 ng/ml doxycycline for 48 and 96 hours. 50,000 cells of each treated and control cells were centrifuged at 350 x g for 5 minutes at 4°C and washed in cold PBS. The cell pellet was resuspended in 50 µl transposase mixture containing 25 µl 2x TD Buffer, 2.5 µl transposase enzyme, 0.01 % digitonin and 22 µl water. The resuspended cell pellet was incubated at 37°C for 60 minutes at 300 rpm. Subsequently, samples were purified using the MinElute PCR purification kit (Qiagen), according to manufacturer instructions. The elution buffer was pre-warmed to 37 °C and during the final elution step, the buffer was left to incubate on the columns for an additional 5 minutes before the final elution spin step. The purified DNA samples were stored at -20°C until library preparation was performed as described (Buenrostro et al., 2015). Final libraries were sequenced (2 x 60) on the Illumina NovaSeq 6000 platform.

Data analysis: The sequencing reads were mapped to the mouse mm9 genome using STAR with the following parameters: --alignIntronMax 1 --alignEndsType EndToEnd. Peak calling was performed by MACS2 (Zhang et al., 2008). ATAC-seq read counts for each sample were then calculated using featureCounts (Liao et al., 2014). DESeq2 (Love et al., 2014) was used to visualize the PCA plot and identify the differential peaks using cutoff p-value <0.05. ChIPseeker R package (Yu et al., 2015) was used to annotate genomic features of determined peaks with the parameter 'tssRegion=c (-5000, 5000)'. The peaks located in the promoter and enhancer (http://enhanceratlas.org/data/download/enhancer/mm/MEF_E13.5.bed) regions were selected for the motif analysis. Homer (Heinz et al., 2010) findMotifsGenome.pl was used to identify TF motifs enriched with the parameter '-size 2000'. Homer 'makeTagDirectory' followed by and 'makeUCSCfile' were used to create bedGraph files for the visualization of ATAC peaks on the UCSC genome browser.

Single Cell RNA Sequencing: Single-cell RNA-seq data was processed as preciously described (Gautam et al., 2021). Briefly, we used counts modes of cellragner (Zheng et al., 2017) to generate read count matrices from the fastq files. The reference genome index of mm10 was downloaded from 10x Genomics. Count and fragment data were imported into Seurat (Hao et al., 2021) objects, and the QC was performed to remove outlier based on the total UMI, detected gene number and % mitochondrial genes. Major cell classes were manually annotated based on the cluster-specific markers. For the identification of cluster biomarkers, we used the Seurat 'FindMarkers' function (min.pct = 0.25, logfc.threshold = 0.25), which detects DEGs for each cluster. Module scores (related to Figure S5D) were calculated using Seurat AddModuleScore function with cluster 3, 4, 7, 9, 13 and 15 DEG sets, and superimposed on the tSNE plot of single-cell RNA-seq cells from published studies (Nestorowa et al., 2016). HPC reprogramming trajectories were inferred by Palantir (Setty et al., 2019) according to the standard workflow. The enrichment of Reactome pathway genes in our Seurat clusters was analysed using ReactomeGSA (Griss et al., 2020) R package with default parameters.

References

Buenrostro, J.D., Wu, B., Chang, H.Y., and Greenleaf, W.J. (2015). ATAC-seq: A Method for Assaying Chromatin Accessibility Genome-Wide. Curr Protoc Mol Biol *109*, 21 29 21-21 29 29. 10.1002/0471142727.mb2129s109.

Gautam, P., Hamashima, K., Chen, Y., Zeng, Y., Makovoz, B., Parikh, B.H., Lee, H.Y., Lau, K.A., Su, X., Wong, R.C.B., et al. (2021). Multi-species single-cell transcriptomic analysis of ocular compartment regulons. Nat Commun *12*, 5675. 10.1038/s41467-021-25968-8.

Griss, J., Viteri, G., Sidiropoulos, K., Nguyen, V., Fabregat, A., and Hermjakob, H. (2020). ReactomeGSA - Efficient Multi-Omics Comparative Pathway Analysis. Mol Cell Proteomics *19*, 2115-2125. 10.1074/mcp.TIR120.002155.

Hao, Y., Hao, S., Andersen-Nissen, E., Mauck, W.M., 3rd, Zheng, S., Butler, A., Lee, M.J., Wilk, A.J., Darby, C., Zager, M., et al. (2021). Integrated analysis of multimodal single-cell data. Cell *184*, 3573-3587 e3529. 10.1016/j.cell.2021.04.048.

Heinz, S., Benner, C., Spann, N., Bertolino, E., Lin, Y.C., Laslo, P., Cheng, J.X., Murre, C., Singh, H., and Glass, C.K. (2010). Simple combinations of lineage-determining transcription factors prime cis-regulatory elements required for macrophage and B cell identities. Mol Cell *38*, 576-589. 10.1016/j.molcel.2010.05.004.

Kyba, M., Perlingeiro, R.C., and Daley, G.Q. (2002). HoxB4 confers definitive lymphoid-myeloid engraftment potential on embryonic stem cell and yolk sac hematopoietic progenitors. Cell *109*, 29-37. 10.1016/s0092-8674(02)00680-3.

Liao, Y., Smyth, G.K., and Shi, W. (2014). featureCounts: an efficient general purpose program for assigning sequence reads to genomic features. Bioinformatics *30*, 923-930. 10.1093/bioinformatics/btt656.

Love, M.I., Huber, W., and Anders, S. (2014). Moderated estimation of fold change and dispersion for RNA-seq data with DESeq2. Genome Biol *15*, 550. 10.1186/s13059-014-0550-8.

Nestorowa, S., Hamey, F.K., Pijuan Sala, B., Diamanti, E., Shepherd, M., Laurenti, E., Wilson, N.K., Kent, D.G., and Gottgens, B. (2016). A single-cell resolution map of mouse hematopoietic stem and progenitor cell differentiation. Blood *128*, e20-31. 10.1182/blood-2016-05-716480.

Scialdone, A., Tanaka, Y., Jawaid, W., Moignard, V., Wilson, N.K., Macaulay, I.C., Marioni, J.C., and Gottgens, B. (2016). Resolving early mesoderm diversification through single-cell expression profiling. Nature *535*, 289-293. 10.1038/nature18633.

Setty, M., Kiseliovas, V., Levine, J., Gayoso, A., Mazutis, L., and Pe'er, D. (2019). Characterization of cell fate probabilities in single-cell data with Palantir. Nat Biotechnol *37*, 451-460. 10.1038/s41587-019-0068-4.

Solaimani Kartalaei, P., Yamada-Inagawa, T., Vink, C.S., de Pater, E., van der Linden, R., Marks-Bluth, J., van der Sloot, A., van den Hout, M., Yokomizo, T., van Schaick-Solerno, M.L., et al. (2015). Whole-transcriptome analysis of endothelial to hematopoietic stem cell transition reveals a requirement for Gpr56 in HSC generation. J Exp Med *212*, 93-106. 10.1084/jem.20140767.

Sroczynska, P., Lancrin, C., Pearson, S., Kouskoff, V., and Lacaud, G. (2009). In vitro differentiation of mouse embryonic stem cells as a model of early hematopoietic development. Methods Mol Biol *538*, 317-334. 10.1007/978-1-59745-418-6_16.

Yu, G., Wang, L.G., and He, Q.Y. (2015). ChIPseeker: an R/Bioconductor package for ChIP peak annotation, comparison and visualization. Bioinformatics *31*, 2382-2383. 10.1093/bioinformatics/btv145.

Zhang, Y., Liu, T., Meyer, C.A., Eeckhoute, J., Johnson, D.S., Bernstein, B.E., Nusbaum, C., Myers, R.M., Brown, M., Li, W., and Liu, X.S. (2008). Model-based analysis of ChIP-Seq (MACS). Genome Biol *9*, R137. 10.1186/gb-2008-9-9-r137.

Zheng, G.X., Terry, J.M., Belgrader, P., Ryvkin, P., Bent, Z.W., Wilson, R., Ziraldo, S.B., Wheeler, T.D., McDermott, G.P., Zhu, J., et al. (2017). Massively parallel digital transcriptional profiling of single cells. Nat Commun *8*, 14049. 10.1038/ncomms14049.
