# Supplementary material for: Competing dynamic gene regulatory networks involved in fibroblast reprogramming to hematopoietic progenitor cells
Source: Stem Cell Reports. 2025 Apr 3;20(5):102473. doi: 10.1016/j.stemcr.2025.102473 (PMC12143154; doi:10.1016/j.stemcr.2025.102473)
Supplement: Document S2. Article plus supplemental information [file mmc7.pdf]

Stem Cell Reports
Article
OPEN ACCESS

Competing dynamic gene regulatory networks involved in fibroblast reprogramming to hematopoietic progenitor cells

Samiyah Shafiq,[1,2] Kiyofumi Hamashima,[2] Laura A. Guest,[1] Ali H. Al-anbaki,[3] Fabio M.R. Amaral,[4] Daniel H. Wiseman,[1] Valerie Kouskoff,[5] Georges Lacaud,[3,6] Yuin-Han Loh,[2,6] and Kiran Batta[1,6,7,*]
[1]Epigenetics of Haematopoiesis Laboratory, Division of Cancer Sciences, The University of Manchester, Manchester, UK
[2]Cell Fate Engineering and Therapeutics Lab, Cell Biology and Therapies Division, Institute of Molecular and Cell Biology (IMCB), Agency for Science, Technology and Research (A(*)STAR), Singapore, Republic of Singapore
[3]Stem Cell Biology Group, Cancer Research UK Manchester Institute, The University of Manchester, Manchester, UK
[4]Leukaemia Biology Laboratory, Cancer Research UK Manchester Institute, The University of Manchester, Manchester, UK
[5]Developmental Haematopoiesis Group, Division of Developmental Biology and Medicine, The University of Manchester, Manchester, UK
[6]Senior author
[7]Lead contact
*Correspondence: kiran.batta@manchester.ac.uk
https://doi.org/10.1016/j.stemcr.2025.102473

SUMMARY

Direct reprogramming of somatic cells offers a potentially safer therapeutic approach to generate patient-specific hematopoietic cells. However, this strategy is limited by stochasticity of reprogramming. Investigating the gene regulatory networks involved during reprogramming would help generate functional cells in adequate numbers. To address this, we developed an inducible system to reprogram fibroblasts to hematopoietic progenitor cells by ectopically expressing the two transcription factors SCL and LMO2. Transcriptome and epigenome analysis at different stages of reprogramming revealed uniform silencing of fibroblast genes and upregulation of the hemogenic endothelial program. Integrated analysis suggested that the transcription factors FLI1, GATA1/2, and KLF14 are direct targets of SCL/LMO2, which subsequently induce the hematopoietic program. Single-cell RNA sequencing revealed conflicting and competing fate decisions at intermediate stages of reprogramming. Inhibiting signaling pathways associated with competing neuronal fate enhanced reprogramming efficiency. In conclusion, this study identifies early/intermediate reprogramming events and associated pathways that could be targeted to improve reprogramming efficiency.

INTRODUCTION

Lineage-specifying transcription factors (TFs), when overexpressed in distant somatic cell types, can reconfigure chromatin organization and gene expression programs to induce cell fate change (Wang et al., 2021). The forced expression of TFs has enabled the generation of a variety of cell types with translational potential; however, further in-depth characterization of reprogramming events and reprogrammed cells is needed to support their use in the clinic. One major limitation of direct reprogramming methodologies is that only a minority of the starting cell populations successfully reprogram, suggesting mechanisms impeding cell fate transition. Several reports, aimed at direct reprogramming to different cell types, suggest that epigenetic and transcriptional heterogeneity, and cell cycle status, could contribute to the stochasticity of reprogramming (Zhou et al., 2016, 2019).

Transfusions of blood cells are used in the clinic to treat hematological cancers and other genetic disorders (Gratwohl et al., 2015). Lack of available donors and robust methods to expand hematopoietic stem cells (HSCs) prompted researchers to look for alternate methods such as directed differentiation of induced pluripotent stem cells (iPSCs). However, protocols that attempted directed differentiation to functional HSCs have been met with limited success (Zheng et al., 2023). An alternative safer approach to directed differentiation is direct reprogramming of somatic cells, which bypasses the need for an iPSC intermediate. Several groups have reprogrammed distinct somatic cell types to hematopoietic stem and progenitor cells (HSPCs) (Batta et al., 2014; Pereira et al., 2013; Riddell et al., 2014; Sandler et al., 2014). Of note, Lis et al. showed that endothelial cells could be reprogrammed to HSC-like cells (Lis et al., 2017). However, unlike fibroblasts, it is challenging to purify and expand endothelial cells in sufficient numbers to induce direct reprogramming.

Our group has shown that overexpression of five TFs (GATA2, ERG, LMO2, SCL, and RUNX1c) can reprogram mouse embryonic fibroblasts (MEFs) to induced HSPCs (iHSPCs) (Batta et al., 2014). Importantly, we have identified that, out of five TFs, SCL and LMO2 alone were sufficient to induce HSPC phenotype (Goode et al., 2016). Inclusion of the additional two TFs, RUNX1 and BMI1, remarkably enhanced the generation of iHSPCs with long-term multilineage repopulating capacity (Cheng et al., 2016). SCL drives the specification of hemogenic endothelium (HE), and LMO2 acts as a scaffold for SCL (El Omari et al., 2013; Porcher et al., 2017). Interestingly, SCL orchestrates the divergence of hematopoietic phenotype in the mesoderm by actively binding to cardiac enhancers and repressing cardiogenesis (Van Handel et al., 2012).

Published by Elsevier Inc. on behalf of International Society for Stem Cell Research.
This is an open access article under the CC BY license (http://creativecommons.org/licenses/by/4.0/).

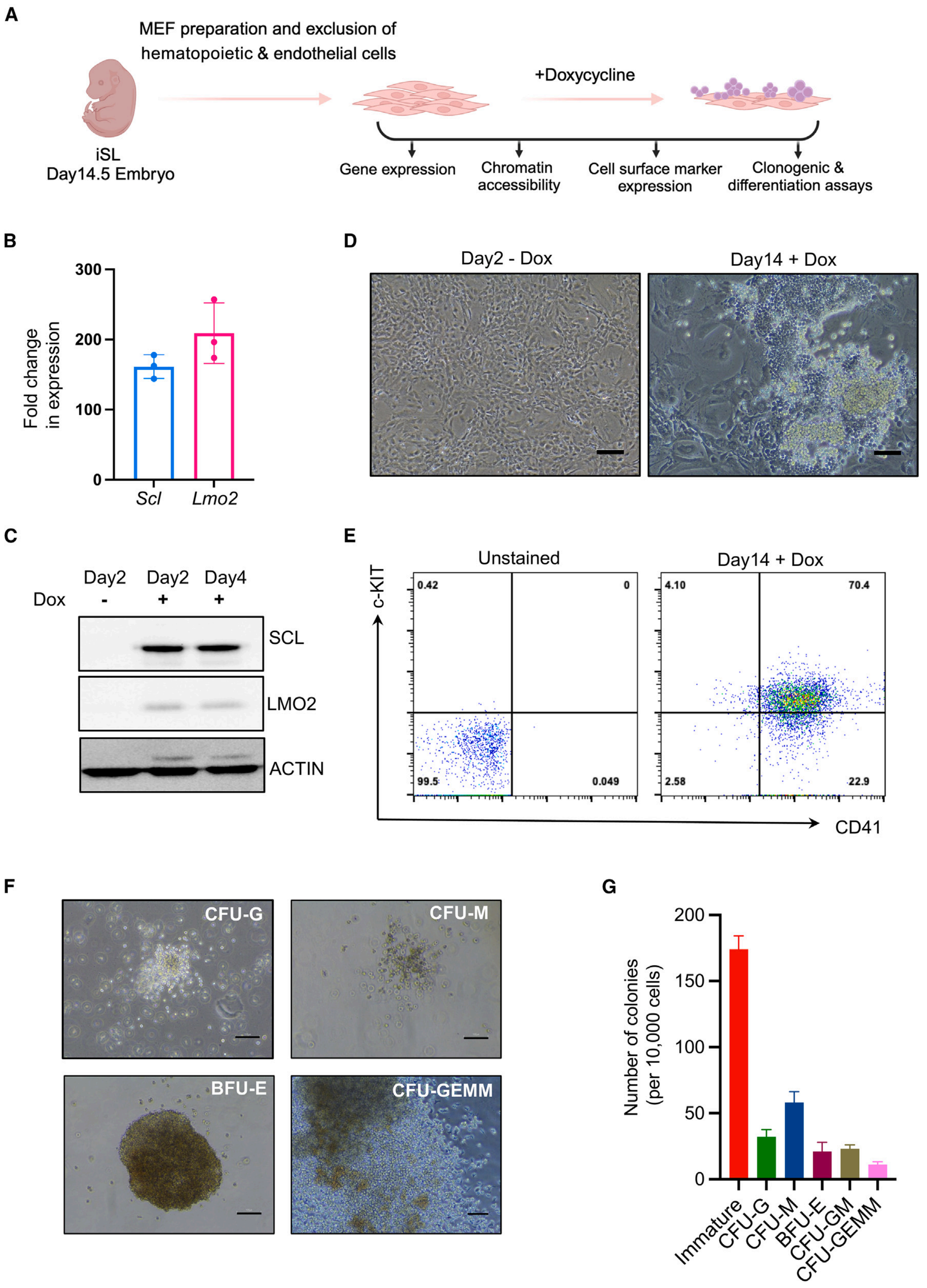

(legend on next page)

The mechanism behind SCL and LMO2-mediated induction of hematopoietic cell fate in fibroblasts and whether these factors have any role in suppressing fibroblast identity is unknown. Comprehending the exact mechanisms underlying specific cell fate transitions would enable the refinement of existing protocols and potentially uncover novel approaches for more efficient reprogramming systems. However, the low efficiencies of reprogramming often conceal the underlying molecular mechanisms involved in efficient cell fate change. The advent of single-cell technologies measuring multiple phenotypes have enabled researchers to identify intricate pathways involved in reprogramming (Biddy et al., 2018).

Here, we have developed an inducible mouse model to express SCL and LMO2 in cell types of choice. MEFs from these mice can be reprogrammed to hematopoietic progenitor cells (HPCs) with multilineage potential. Through single-cell gene expression studies, we found gene signatures reflecting hematopoietic and neuronal fate at intermediate stages of reprogramming, suggesting conflicting cell fates induced by the exogenous TFs. Inhibiting neuronal lineage pathway significantly improved reprogramming efficiency.

RESULTS

Direct reprogramming of MEFs to iHPCs by the ectopic expression of SCL and LMO2

To investigate how SCL and LMO2 together induce a hematopoietic phenotype in fibroblasts, we developed a stable inducible system where we can achieve consistent and homogeneous overexpression of genes of interest. To this end, we have generated an embryonic stem (ES) cell line carrying a *Scl-T2A-Lmo2*-IRES-GFP construct under an inducible promoter (Figure S1A) (Kyba et al., 2002). Addition of doxycycline to this cell line (ES-iSL) uniformly induced the expression of GFP and *Scl* & *Lmo2* (Figures S1B and S1C). Next, we tested the effect of SCL and LMO2 induction on hematopoietic specification from ES cells. FLK1+ve hemangioblasts, differentiated from ES-iSL line, were cultured either in the presence or absence of doxycycline, and cells were harvested at days 1–3 (Figure S1D). Induction of SCL and LMO2 promoted HE specification as seen by the increase in number of TIE2 and CD41 double-positive cells (Figure S1E). Indeed, clonogenic assays with day 3 doxycycline-treated hemangioblast cultures showed an increased in the number of all types of hematopoietic colonies indicating the positive effect of SCL and LMO2 induction on hematopoietic specification (Figure S1F). Together, these data confirm the functionality of the ES-iSL line, and therefore, these cells were injected into blastocysts to generate a stable mouse line with inducible expression of SCL and LMO2.

To investigate the dynamics of reprogramming to HPCs, we decided to use MEFs as a starting population as they are easy to collect and expand. E14.5 MEFs were harvested from the inducible mouse line (iSL) and were depleted of contaminant hematopoietic and endothelial cells (Figure 1A). Expression of SCL and LMO2 was induced by treatment with doxycycline, and the fibroblasts were cultured in hematopoietic media to facilitate direct reprogramming. 48 h post induction, the cells exhibited much higher levels of *Scl* and *Lmo2* verified at RNA and protein levels (Figures 1B and 1C). Changes in cellular morphology could be observed in doxycycline-treated conditions as early as day 4–6; however, by day 8–14, clear cobblestone and suspension colonies of blood-like cells could be seen (Figure 1D). Reprogrammed cells expressed HSPC markers CD41 and c-KIT (Figure 1E). Colony-forming unit (CFU) assays revealed that induced HPCs (iHPCs) could generate macrophage, granulocyte, erythroid, and mixed lineage colonies, demonstrating their multilineage potential

Figure 1. Ectopic expression of SCL and LMO2 induces reprogramming of fibroblasts to hematopoietic progenitor cells

(A) Schematic representation of experimental methodology. Day 14.5 MEFs from iSL (inducible SCL and LMO2) mouse line were cultured in presence of doxycycline to induce *Scl* and *Lmo2* expression. Reprogramming cells were taken at different time points for molecular and functional characterization.

(B) Fold change in the expression of *Scl* and *Lmo2* after 48 h of doxycycline treatment with respect to vehicle-treated MEFs ($N = 3$, MEFs isolated from three different embryos).

(C) Representative western blot images showing SCL and LMO2 protein expression after 2 and 4 days of doxycycline treatment. Vehicle-treated MEFs at day 2 were used as a control ($N = 3$, MEFs isolated from three different embryos).

(D) Representative bright-field images of vehicle-treated MEFs at day 2 and doxycycline (Dox)-treated MEFs at day 14.

(E) Flow cytometry analysis of day 14 reprogrammed cells.

(F) Representative bright-field images of the different types of colonies observed upon plating day 14 reprogrammed cells. CFU-G, granulocyte; CFU-M, macrophage; CFU-GM, granulocyte, monocyte; BFU-E, erythroid; CFU-GEMM, granulocyte, erythroid, monocyte, megakaryocyte.

(G) Number of different types of colonies observed from 10,000 plated day 14 reprogrammed MEFs for 2 independent embryos, each performed in triplicates. Error bars represent SEM. Scale bars represent 100 μm.

See also Figure S1.

(Figures 1F and 1G). Together, these results show that MEFs harvested from the iSL mouse line can be successfully reprogrammed to iHPCs with multilineage clonogenic capacity.

Characterization of SCL- and LMO2-induced HPCs

Next, we characterized day 14 reprogrammed cells by gene expression analysis and differentiation assays. As expected, we observed downregulation of the fibroblast-specific genes *Fbn1* and *Acta2* and upregulation of the HSPC-specific genes *Gata2* and *Gfi1* (Figure 2A). Erythroid, myeloid, and megakaryocytic lineage-specific genes such as *Hba* & *Hbb*, *ITGAM* & *Pu.1*, and *Pf4*, respectively, also showed increased expression (Figure 2B). To further validate the differentiation potential of iHPCs, we performed myeloid, erythroid, and lymphoid differentiation assays by culturing day 14 cells in permissive culture conditions. When cultured under conditions to promote erythroid specification, we observed cells positive for common erythroid cell surface marker, CD71, within 15 days (Figure 2C). Cytospin analysis of these cells further confirmed the presence of erythroblasts and enucleated erythrocytes (Figure 2D). In reprogrammed cultures, we observed CD11b/Ly6C-positive monocytes and CD11b/Gr1-positive granulocytes (Figure 2E). In addition, we also observed the morphology of various differentiated cell types such as monocytes, megakaryocytes, and neutrophils (Figure 2F). To promote lymphoid differentiation, the iHPCs were co-cultured with feeder cell lines OP9 and OP9-DLL1 to enable B and T cell differentiation, respectively. Co-culture of reprogrammed cells on OP9 stromal cells induced differentiation to B220-positive cells but CD19 was not detected above background levels (Figure S2A). When cultured on OP9-DLL1 stromal cells, reprogrammed cells differentiated to CD25 but never showed expression of CD3 (Figure S2B). Together, these data suggest differentiation capacity of iHPCs to multiple hematopoietic cell types.

To test the *in vivo* functionality of the reprogrammed cells, we first fluorescently labeled MEFs by transducing them with pSin-GFP. Reprogrammed c-KIT/GFP double-positive cells were sorted and injected into sub-lethally irradiated NSG mice (Figure S2C). Short-term engraftment was detected in the mice up to 7 weeks post injection (Figures 2G and S2D), as measured by the presence of GFP-positive cells within the peripheral blood. However, we did not see any engraftment in the bone marrow or blood when the mice were sacrificed at week 16. Together, our data showed that iHPCs can differentiate to multiple lineages *in vitro* and can engraft recipient mice *in vivo* short-term, suggesting that SCL and LMO2 together can induce an HPC phenotype in fibroblasts.

SCL- and LMO2-induced HPC phenotype occurs via HE state

To investigate global early and late molecular events involved in reprogramming, we performed transcriptome analysis on day 2 and day 4 doxycycline-treated cells, and c-KIT^{+}-sorted day 14 reprogrammed cells, along with control day 2 vehicle-treated fibroblasts. Principal component analysis (PCA) showed that control and day 14 reprogrammed cells are transcriptionally very distinct from each other whereas intermediate day 2 and day 4 reprogrammed cells clustered closely (Figure 3A). We observed an increase in the number of differentially expressed genes (DEGs) as the reprogramming progressed, indicating a gradual change in identity as opposed to direct rapid transdifferentiation (Figure 3B; Tables S1, S2, and S3). The top 20 most significantly upregulated genes in the day 2 reprogrammed cells include the hematopoietic and endothelial TFs *Hhex* and *Sox7*, and neuronal marker gene *Tubb3* (Latremoliere et al., 2018). The increase in number of DEGs observed in day 4 compared with day 2 reprogrammed cells suggests a role for indirect targets of SCL and LMO2 in reprogramming. Possible indirect targets of SCL and LMO2 include mostly endothelial genes, e.g., *Acer2* and *CDH5*, and a few hematopoietic-specific genes, e.g., *Gata1* and *Gfi1* (Figure S3A). Gene Ontology (GO) analysis of upregulated genes in day 4 reprogrammed cells revealed pathways related to cell adhesion and endothelium development (Figure 3C). Downregulated genes in day 4 reprogrammed cells are linked to extracellular matrix and cell-cell junction signatures indicative of loss of fibroblast identity (Figure S3B). Gene set enrichment analysis (GSEA) comparing reprogrammed cells against controls showed negative enrichment of E2F targets in day 2 and day 4 reprogrammed cells but not in day 14 sorted HPCs, suggesting that initial reprogramming is negatively linked to active cell cycle status (Figure 3D).

We next performed clustering on DEGs to detect clusters of genes with different expression patterns during reprogramming. Clustering of day 2 vs. control DEGs revealed four main clusters (Figure 3E). Cluster 1 genes are transiently expressed in intermediate reprogrammed cells, and these include endothelial-specific genes such as *Sox7*, *Sox18*, and *Lyve1* (Figure 3F). Transient expression of these genes suggests that reprogramming of fibroblasts to HPCs occurs via an intermediate endothelial state (Figures 3E and 3F). A dramatic downregulation of fibroblast gene expression (clusters 3 and 4) in the day 4 reprogrammed cells suggests that reprogramming is initiated in most of the starting fibroblast populations (Figure 3F). Cluster 2 genes are gradually upregulated during reprogramming, suggesting their involvement in the hematopoietic specification. Clustering of day 4 vs. control DEGs further reiterated the enrichment of an endothelial gene signature in

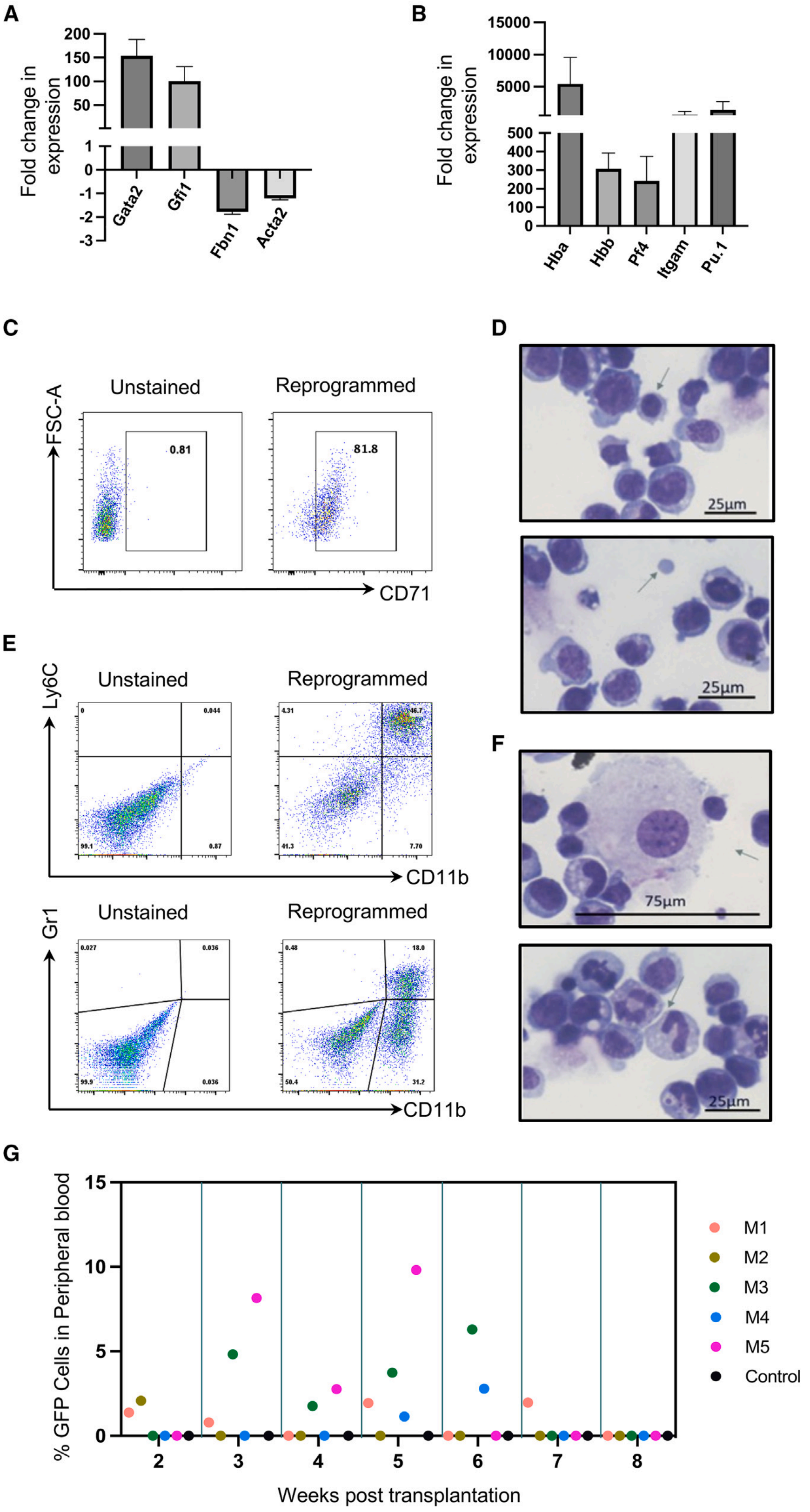

Figure 2. Reprogrammed hematopoietic progenitor cells have multilineage differentiation capacity

(A and B) Fold change in expression levels of hematopoietic or fibroblast-specific (A) or mature lineage-specific (B) genes in day 14 reprogrammed cells as compared with day 2 control cells ($N \geq 3$, MEFs from three or more different embryos).

(C and E) Flow cytometry analysis of reprogrammed cells measuring the expression of erythroid markers (C) or monocytic or granulocytic markers (E).

(D and F) May Grunwald Giemsa staining of cells differentiated to erythroid (D) or myeloid lineages (F).

(G) Percentage of GFP-positive cells within peripheral blood of recipient mice (5 mice labeled M1–M5) at indicated weeks post transplantation of reprogrammed cells. A mouse that did not receive any reprogrammed cells was used as a control. Scale bars represent 100 μm. Error bars represent SEM.

See also Figure S2.

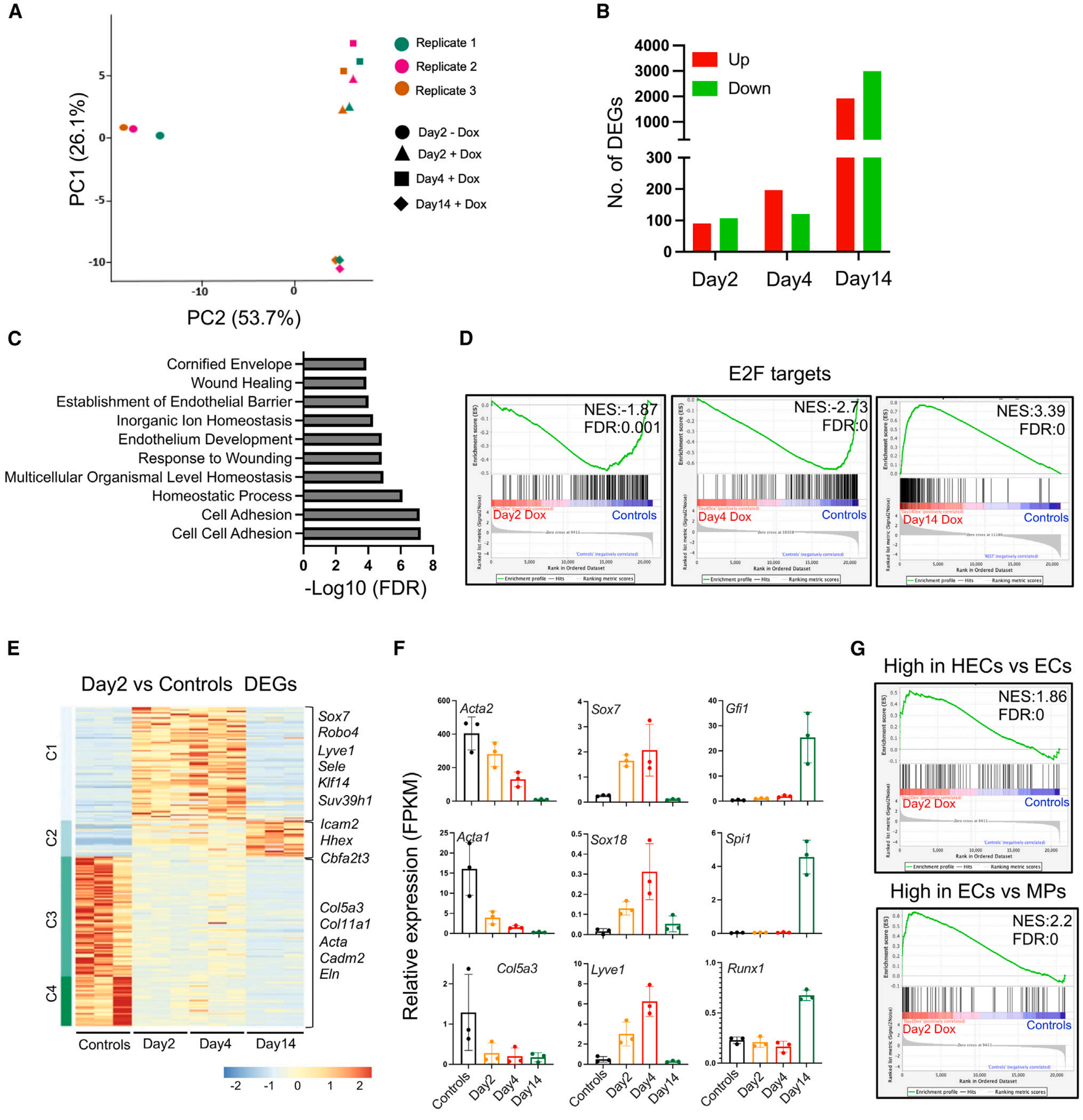

Figure 3. Reprogramming to hematopoietic progenitor cells occurs via hemogenic endothelial stage

(A) Principal component analysis of transcriptome data performed on controls and day 2, day 4, and day 14 (c-KIT positive) reprogramming cells ($N = 3$, MEFs from three different embryos).

(B) Bar chart showing the number of differentially expressed genes (DEGs) at day 2, 4, and 14 reprogramming cells as compared with control cells.

(C) GO analysis of upregulated genes in day 4 reprogramming cells as compared with controls.

(D) GSEA plots showing differential enrichment of E2F target genes in day 2, 4, and 14 reprogramming cells as compared with controls.

(E) Heatmap showing the expression levels of DEGs in day 2 reprogramming cells as compared with control cells.

(F) Relative expression levels of indicated DEGs in reprogramming and control cells.

(legend continued on next page)

reprogramming intermediates (Figure S3C). Together, these data suggest that reprograming to HPCs is mimicking embryonic development. To further confirm this, we performed GSEA analysis investigating genes uniquely expressed in endothelial and HE cells (Scialdone et al., 2016; Solaimani Kartalaei et al., 2015). Results show that day 2 and day 4 reprogramming cells are positively enriched for genes overexpressed in HE cells compared to endothelial cells, and endothelial cells compared to mesodermal progenitor cells (Figures 3G and S3D). Clustering of day 14 DEGs compared to the control revealed 4 main clusters, two of which were composed of mainly hematopoietic genes (Figure S3E). In conclusion, transcriptome analysis at distinct stages of reprogramming suggests a downregulation of fibroblast identity followed by induction of an HE phenotype.

Chromatin accessibility dynamics during early stages identifies downstream TF networks involved in reprogramming

To investigate early changes in the chromatin accessibility landscape during reprogramming, we performed assay for transposase-accessible chromatin using sequencing (ATAC-seq) analysis on reprogramming cells collected at day 2 and day 4. Consistent with transcriptional changes, PCA analysis of early reprogramming cells showed substantial changes in chromatin accessibility (Figure 4A). We observed an increase in the number of differentially accessible regions (DARs) from day 2 to day 4 reflecting again dynamic transcriptional changes (Figures S4A; Tables S4 and S5). Majority of DARs mapped to distal elements (Figure 4B). Interestingly, both *Scl* and *Lmo2* endogenous gene loci gained accessibility in day 2/4 reprogrammed cells suggesting a positive feedback loop (Figure 4C). Other TF genes that gained accessibility in the early stages of reprogramming include *Hhex*, *Sox7*, *Gfi1*, *Gata2*, and *Cebpb*, suggesting their role in downstream reprogramming events (Figures 4C and S4B). We did not observe major changes in the accessibility of regions mapped to fibroblast genes *Acta2*, *Fbn1*, and *Col5a3* (Figure S4C). This suggests that while SCL and LMO2 activate hematopoietic phenotype by opening hematopoietic regulatory elements, they do not directly repress fibroblast gene expression.

Regions that gained accessibility in day 4 reprogramming cells showed enrichment for TF ETS (SCL), FLI1, GATA2, and ETV2 motifs (Figure 4D). Regions that lost accessibility are enriched for TF motifs belonging to the AP-1 family including AP-1, BATF, and FOSL2 but not ETS family, suggesting that loss of chromatin accessibility during reprogramming is not mediated by SCL and LMO2 (Figure S4D). In contrast to SCL's known role in suppression of cardiac lineage (Van Handel et al., 2012), we did not observe any change in the accessibility or expression of cardiac TFs. However, SCL direct targets such as *Cbfa2t3*, *Hhex*, and *Scl* (endogenous) are upregulated in day 2 reprogramming cells with genomic regions linked to these genes opening upon induction with doxycycline (Figure S4E) (Wilson et al., 2009).

Although widespread chromatin changes were observed in day 2 and day 4 reprogramming cells, only a minority of the genes linked to DARs were differentially expressed (Figures S4E and S4F). However, we did observe a better overlap between genes linked to day 2 DARs with genes differentially expressed in day 4 reprogramming cells, suggesting that chromatin changes precede transcriptional alterations (Figure S4G). TFs whose motifs were commonly enriched in more accessible regions in day 2 and day 4 reprogrammed cells included ERG, GATA1/2, FLI1, and ETV1/2 (Figure 4E). Out of these TFs, GATA1, GATA2, FLI1, and KLF14 are overexpressed in the day 2 and day 4 reprogramming cells, suggesting their potential role in driving reprogramming (Figure 4F). Interestingly, we also observed enrichment of motifs linked to neuronal TFs such as ASCL1 and NEUROD1 in the day 4 reprogramming cells (Figure 4E). These data, together with dramatic upregulation of neuronal marker (*Tubb3*) gene expression, suggest a potential role for SCL and LMO2 in induction of neuronal program in fibroblasts. In conclusion, our data suggest that early reprogramming of fibroblasts to HPCs involves dynamic changes in chromatin configuration, predominantly mediated by SCL and LMO2 together, but later driven by the TFs GATA1/2 and FLI1.

Investigating stochasticity of reprogramming to HPCs using scRNA sequencing

Despite induction of SCL and LMO2 in most cells in our model system, only a minority of these MEFs undergo reprogramming (0.5%–5%), suggesting potential barriers (Figure S5A). While bulk cell RNA and ATAC-seq aided in identification of transcription networks and cellular landscapes at different stages of reprogramming, they did not explain why only a minority of cells undergo successful reprogramming. To decipher the heterogeneity of transcriptional responses that may contribute to the stochasticity

(G) GSEA plots showing enrichment of genes upregulated in hemogenic endothelial cells (HECs) compared to endothelial cells (ECs) (Solaimani Kartalaei et al., 2015) (top) and genes upregulated in ECs with respect to mesodermal progenitors (MPs) (Scialdone et al., 2016) in day 2 reprogramming cells as compared with controls. Error bars represent SEM.
See also Figure S3.

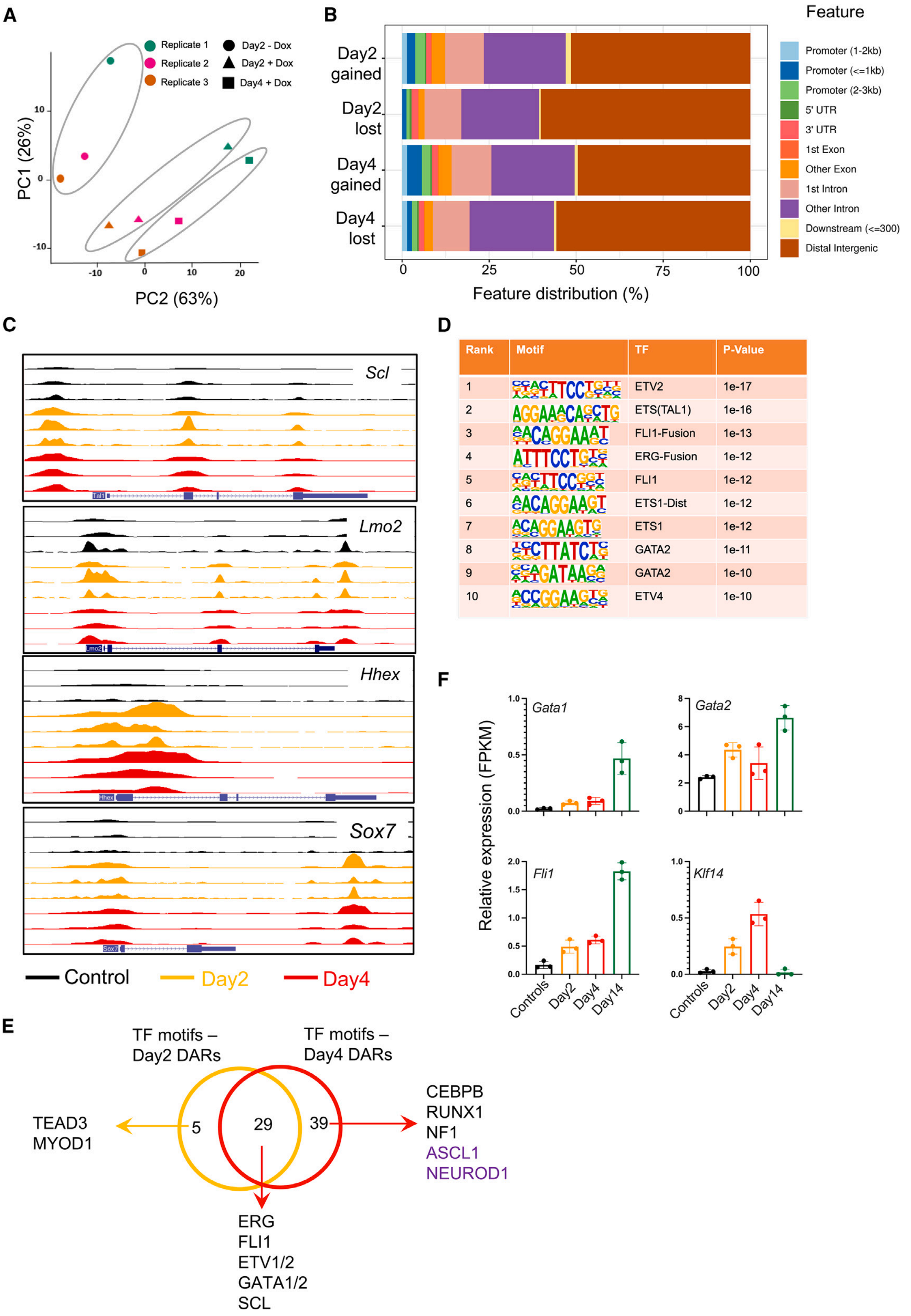

(legend on next page)

of reprogramming, we performed single-cell RNA sequencing (scRNA-seq) at days 2, 4, 8, and 14 of reprogramming (Figures 5A and S5B). Day 14 reprogrammed cells showed different levels of c-KIT expression; therefore, we sorted for c-KIT high and c-KIT low populations (Figure S5B).

t-distributed stochastic neighbor embedding (t-SNE) analysis showed a clear separation of cells based on the time point they were harvested (Figure 5B). Seurat clustering highlighted 22 clusters, 6 of which were unique to day 14 reprogrammed cells, with the remaining composed of control and early/intermediate cells (Figures 5C and S5C). Control fibroblasts were composed of mainly 4 clusters, 3 of which are mostly unique to control cells, and cluster 5 was shared between control cells and day 2 reprogramming cells. Day 2 reprogramming cells were mostly distributed across clusters 1, 5, 8, 10, and 19. However, these clusters also included cells from later time points suggesting diverse kinetics of reprogramming (Figure S5C). The expression of the fibroblast genes *Acta2* and *Prrx1* showed a gradual downregulation from day 2 to day 8 doxycycline-treated cells when compared to control cells, which appeared consistent across most of the treated cells (Figures 5D and S5D). In addition, a significant downregulation of fibroblast gene expression at bulk and single-cell level without any evidence of cell death in fibroblasts suggests that the stochasticity of reprogramming is not mediated by failure to silence fibroblast gene expression (Figures S5E and S5F). Endothelial to hematopoietic transition (EHT) genes, such as *Cdh5*, *Sox7*, *Icam1*, and *Ctla2a*, showed upregulation from little to no expression in day 2 control cells, to increased expression by day 8 of doxycycline treatment, after which expression dropped in reprogrammed HPCs (Figures 5D and S5D). Quantification of cells that expressed EHT genes *Fli1*, *Hhex*, and *Icam* revealed that a large proportion (~25%) of day 2, 4, and 8 reprogramming cells express these genes (Figure 5E). This suggests that inability to initiate a hemogenic program is not the main reason for poor reprogramming efficiency.

c-KIT high and c-KIT low day 14 cells did not cluster separately, although c-KIT low cells appeared to express mature blood cell markers *Itgam* and *Hba-a1* suggesting that c-KIT low cells are late progenitors (Figures 5B and 5D). We projected publicly available murine HSPC gene signatures onto our tSNE plots to characterize reprogramming/reprogrammed cells (Nestorowa et al., 2016). Most of the day 14 reprogrammed cells show similarity with megakaryocyte erythroid progenitors (MEPs), common myeloid progenitors (CMPs), and granulocyte-monocyte progenitors (GMPs) but not long-term (LT) or short-term (ST)-HSCs, consistent with their limited *in vivo* potential (Figure S5G). Interestingly, LT-HSC and ST-HSC gene signatures were enriched in early reprogramming cells, perhaps due to transcriptional similarity between HSCs and HE cells. Together, these analyses suggest that the stochasticity of reprogramming is not mainly caused by heterogeneity within the fibroblast population or inability to initiate reprogramming and that instead other factors during reprogramming affect successful transitions.

Trajectory analysis on early and intermediate reprogrammed cells inferred multiple routes to successful reprogramming

Trajectory analysis of single-cell transcriptome data enables inference of cellular hierarchies (Setty et al., 2019). Therefore, to decipher transcriptional landscapes involved in successful reprogramming or lack thereof, palantir trajectory analysis was performed on the reprogramming cell population (Figure 6A). Our analysis treated control fibroblasts as the starting populations and excluded day 14 reprogrammed cells as they appeared very distinct and lacked continuity from other time points. Analysis showed that fibroblast populations take 5 trajectories (lineages 1–5) during reprogramming (Figures 6A and S6A). Lineage 1 was taken by very few cells with no observable expression of EHT genes and therefore was treated as an unsuccessful route to reprogramming (Figure 6B). Lineage 2 was the main lineage taken by most cells, which express high levels of EHT markers, and was considered as successful route to reprogramming (Figures 6A and 6B). Lineage 3, like lineage 1, showed early divergence and mainly included actively cycling cluster 5 cells (Figures 6A, S6B, and S6C). Initially, most control fibroblasts (~75%) were in G1 phase of cell cycle (Figure S6D). Following induction of SCL and LMO2, a

Figure 4. Gene regulatory networks involved in reprogramming to hematopoietic progenitor cells

(A) Principal component analysis of ATAC-seq data performed on controls and day 2 and day 4 reprogramming cells ($N = 3$, MEFs from three different embryos).

(B) Genomic annotation of differentially accessible regions (gained/lost) in day 2 and day 4 reprogramming cells as compared with controls.

(C) UCSC browser plots depicting the chromatin accessibility at indicated gene loci in controls and day 2 and day 4 reprogramming cells.

(D) Motif enrichment analysis in chromatin regions that gained accessibility in day 4 reprogramming cells as compared with controls.

(E) Venn diagram showing an overlap of TF motifs that are enriched in promoter regions that are more accessible in day 2 and day 4 reprogramming cells. TFs related to neuronal fate are highlighted in purple.

(F) Relative expression levels of indicated differentially expressed TF genes in reprogramming and control samples.

Error bars represent SEM. See also Figure S4.

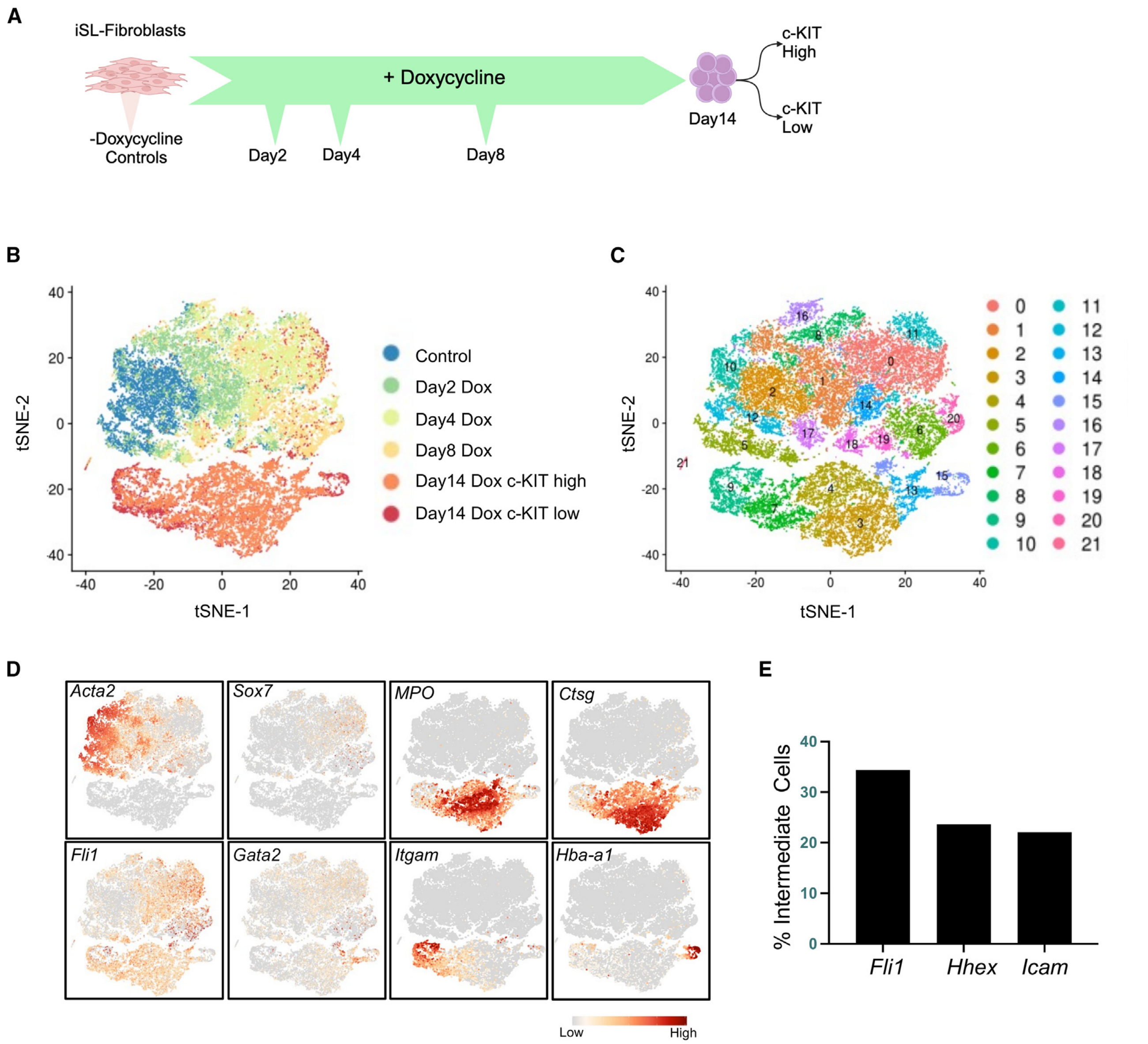

Figure 5. Single-cell transcriptome analysis of reprogramming intermediates
(A) Schematic overview of single-cell RNA sequencing workflow.
(B) t-distributed stochastic neighbor embedding (t-SNE) plot based on highly variable genes for all cells passing filtering thresholds, colored by sample type.
(C) t-SNE plot displaying 22 transcriptionally distinct subpopulations as determined by unsupervised clustering.
(D) Visualization of expression of indicated genes on t-SNE distribution.
(E) Bar chart showing percentage of intermediate cells (days 2, 4, and 8) expressing indicated HE-specific genes.
See also Figure S5.

significant downregulation of active cell cycle genes was observed in day 2/4 reprogrammed cells (Figure 3D). As reprogramming progressed from day 2 to day 8, the proportion of cells in G2/M and S phase decreased (Figure S6D). These observations suggest that G1 phase, as opposed to G2/M and S phase, primes the active reprogramming process. However, lineage 3 cells also express EHT markers, indicating that actively cycling cells can also reprogram into HPCs (Figure 6B). This suggests the possibility that these cells may transition back to G1 phase prior to reprogramming. However, this hypothesis is speculative and lacks experimental evidence. The lack of induction of EHT genes

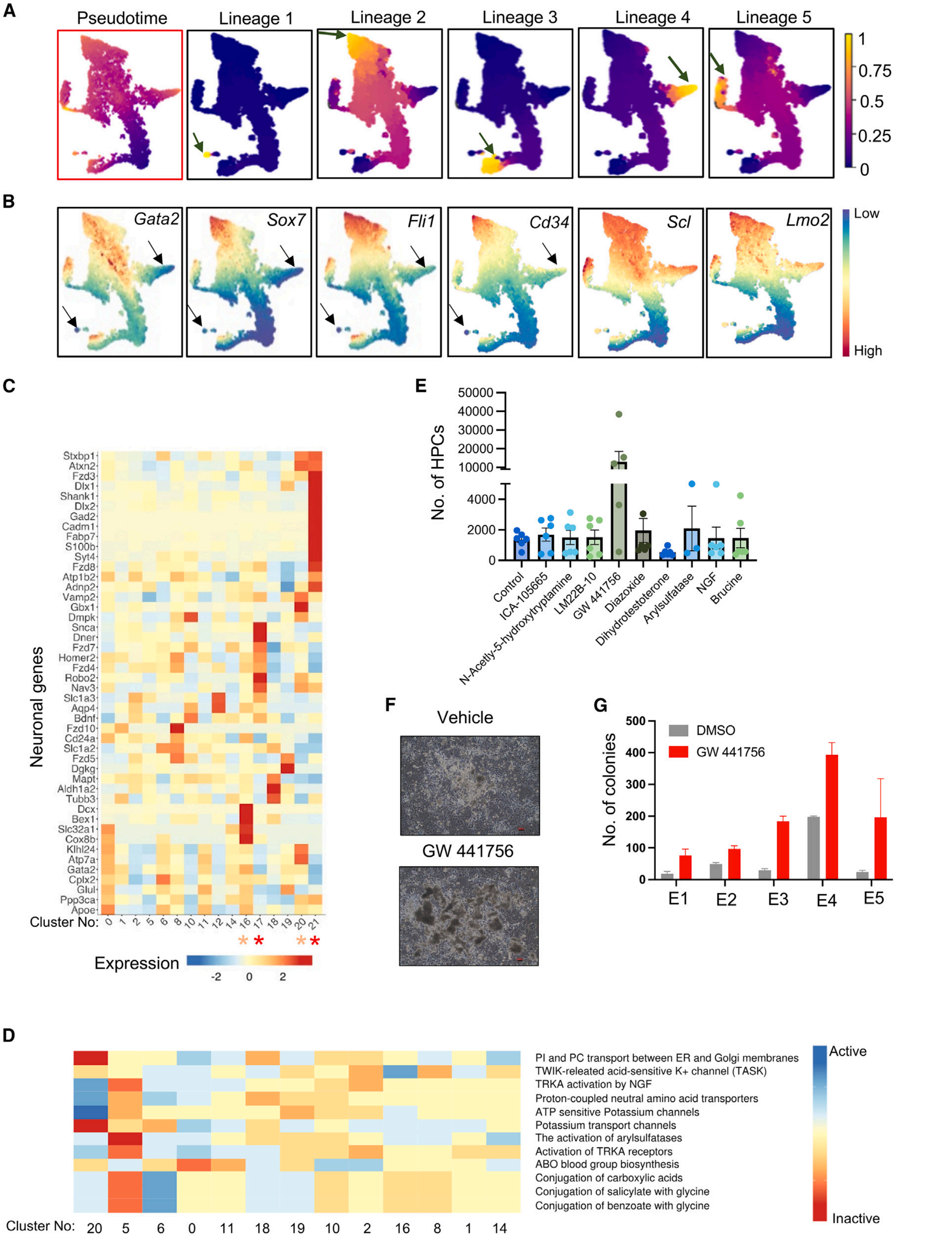

(legend on next page)

but not *Scl* and *Lmo2* within lineage 4 suggested that this trajectory was not the one taken by cells undergoing successful reprogramming. EHT marker expression is also observed at the end of lineage 5 suggesting another successful route to reprogramming (Figure 6B). In summary, we observed that lineages 2, 3, and 5 represent successful routes to reprogramming whereas lineages 1 and 4 are unsuccessful routes.

Bulk cell transcriptome and ATAC-seq data suggested activation of neuronal networks in intermediate reprogramed cells. In our single-cell data, we have observed induction of neuronal genes such as *Robo2 Tubb3*, *Snca*, *Dner*, *Fzd7*, and *Fzd8* in intermediate reprogramming cells (Figure 6C). These genes are predominantly expressed in unsuccessful reprogramming clusters (lineage 1 – cluster 21 and lineage 4 – cluster 17) and clusters that are in transition (clusters 16 and 20) but not the endpoint successful reprogramming clusters (lineage 2 – cluster 11 and lineage 5 – cluster 18) (Figures 6C, S6E, and S6F). Genes uniquely expressed in unsuccessful reprogrammed cluster 21 are indeed related to several neuronal processes (Figure S6G). Given SCL's role in neurogenesis (Bradley et al., 2006), we hypothesize that induction of neuronal gene expression in intermediate reprogramming cells may result in a conflicting state, which could impede reprogramming to HPCs.

To provide insights into signaling pathways active in cells undergoing reprogramming, pathway analysis on genes linked to early and intermediate reprogramming clusters was performed (Figure 6D). Interestingly, the cluster that showed the greatest difference in its pathway activity when compared to the rest included was cluster 20, with most cells in transition. We hypothesized that these pathways uniquely up- or downregulated in cluster 20 could play an important role in deciding cellular fate following SCL and LMO2 induction. The pathways that were differentially modulated in cluster 20 include tyrosine kinase receptor (Trka) activation by nerve growth factor (NGF), ATP-sensitive potassium channels, activation of aryl sulfatases, and glycine metabolism (Figure 6D) and therefore were selected for pathway modulation using small-molecule modulators (Figure S6H). To test the effects of targeting these pathways on reprogramming efficiency, MEFs were treated with chemicals from the initiation of reprogramming until day 14, after which the number of HPCs within each well was quantified.

Interestingly, of all the small molecules trialed for their effects on reprogramming, GW 441756, a potent and specific NGF/Trka inhibitor, showed a significant increase in the number of HPCs within the day 14 reprogrammed cultures (Figures 6E and 6F). NGF/Trka signaling plays an important role in neuronal development. Improved reprogramming efficiency by blocking the binding of NGF to Trka reinforces the likeliness of lineage divergence among the reprogramming cell population. To further confirm the positive effect of GW 441756 on reprogramming of fibroblasts rather than on proliferation of reprogrammed cells, we quantified the number of hematopoietic colonies at an early stage of reprogramming. We observed an increase in number of colonies at early stages of reprogramming suggesting improvement in reprogramming efficiency (Figure 6G). In conclusion, our analysis revealed that the conflicting cell fates induced by SCL and LMO2, in part, limit reprogramming to HPCs, and by inhibiting undesired cell fate induction, we could improve reprogramming efficiencies.

DISCUSSION

In this study, we developed a robust methodology to investigate the impact of ectopic expression of SCL and LMO2 in reprogramming somatic fibroblasts to iHPCs.

Figure 6. Reprogramming to hematopoietic progenitor cells occurs via multiple routes

(A) Palantir trajectory analysis of day 2 control and day 2, 4, and 8 doxycycline-treated cells. Cells colored by pseudotime, with different lineage trajectories (lineages 1–5) depicted as different plots and endpoints highlighted by green arrows. In the color bar, 0 represents starting and 1 represents the ending of each lineage trajectory.

(B) Visualization of expression of indicated genes on trajectory plots. Absence of some of their expression in lineages 1 and 4 are highlighted by black arrows.

(C) Heatmap showing the expression of known neuronal genes among early and intermediate clusters of reprogramming cells. Clusters that expressed neuronal genes at high levels are marked with red stars (unsuccessful reprogrammed clusters) or orange stars (transition clusters).

(D) Heatmap showing the activity of pathways in different intermediate and early reprogramming cell clusters.

(E) Bar chart showing the number of HPCs within the wells of reprogramming cells treated with indicated small-molecule modulators. Each experiment was performed with 3 replicates using MEFs from one embryo. The data shown combined 2 separate experiments from two different embryos.

(F) Representative bright-field images of hematopoietic colonies that were observed within wells treated with either vehicle or Trka inhibitor GW441756 after 14 days of reprogramming.

(G) Number of hematopoietic colonies generated following reprogramming of MEFs treated with either vehicle control DMSO or Trka inhibitor GW 441756 (N = 5, MEFs from 5 different embryos E1–E5). Scale bars represent 100 μm. Error bars represent SEM.

See also Figure S6.

We interrogated the transcriptional and *cis*-regulatory region landscape underlying the reprogramming and identified downstream drivers. Trajectory analysis revealed multiple routes to successful reprogramming. We found conflicting cellular fates induced by SCL and LMO2, which in part contributes to unsuccessful reprogramming.

We observed that fibroblast identity was uniformly and gradually erased across all cells, and many early reprogramming cells expressed EHT genes such as *Fli1*, *Hhex*, and *Icam*, suggesting that poor efficiency of reprogramming is not mediated by heterogeneity of the starting populations. This is consistent with observations made in reprogramming to cardiomyocytes where the identity of the starting fibroblast population did not impact the cell fate change induced by TFs (Liu et al., 2017). Several studies found that the proliferative state of the starting cell population negatively impacts reprogramming efficiency (Treutlein et al., 2016; Zhou et al., 2019). Our comprehensive analysis of both bulk and single-cell transcriptomes at intermediate stages of reprogramming demonstrated that majority of these cells are in G1 phase of the cell cycle. This observation suggests that either induction of SCL and LMO2 arrests cells in G1 phase or that the initiation of the reprogramming process requires cells to be in G1 phase. The gradual reduction in the frequency of cells in G2/M and S phase from day 2 to day 8 suggests support for the latter. This implies that the active phase of cell cycle is not permissive to transcriptional changes induced by transgene activation. However, once cells transition to an inactive phase of cell cycle, cell fate change can then be initiated. Our clustering analysis revealed multiple clusters of successfully reprogrammed cells with expression of EHT genes. These are clusters placed along multiple but not all palantir trajectories, suggesting that reprogramming to iHPCs can occur via multiple routes.

Several studies have revealed that reprogramming to hematopoietic fate occurs via an intermediate state called HE (Batta et al., 2014; Pereira et al., 2013). In this study, we also see transient upregulation of endothelial genes. The TFs specifying (e.g., SOX7, GFI1) HE fate are directly induced by SCL and LMO2 in the early stages of reprogramming. *In vivo* repopulation studies showed that reprogrammed cells only have short-term engraftment capacity, suggesting that HE cells generated through the reprogramming process are only capable of giving rise to multipotent progenitor (MPP) cells. Indeed, heterogeneity within HE cells exists with some cells capable of producing HSCs while others commit to MPPs (Dignum et al., 2021). Alternately, HE cells with HSC potential may have been generated; however, the culture conditions used in the study are not permissive to maintain HSCs. Besides HE fate, we have also observed expression of some neuronal genes in some clusters. This was also evident in bulk cell analysis of the day 2 reprogramming cells. The neuronal signatures observed in certain clusters could represent partially reprogrammed neuronal cells. Indeed, clusters with neuronal gene signature were represented in unsuccessful reprogramming trajectories. Upon blocking of the Trka activation by NGF, which is an important signaling pathway playing roles in the survival and differentiation of neurons (Levi-Montalcini, 1987; Thoenen and Edgar, 1985), the reprogramming efficiency to HPCs is increased, suggesting that conflicting cell fate decisions are induced by SCL and LMO2 together. Induction of neuronal fate in fibroblasts is not surprising given that *Scl* is expressed in the central nervous system and plays a role in neuronal differentiation (Achim et al., 2013; Elefanty et al., 1999).

TFs that initiate reprogramming while inducing a desired cell phenotype also actively downregulate gene expression of starting populations (Penalosa-Ruiz et al., 2020). SCL is also shown to maintain hematopoietic phenotype during development by actively repressing other mesodermal lineages (Chagraoui et al., 2018; Van Handel et al., 2012). We did not observe any changes in cardiac genes known to be repressed by SCL either at chromatin levels or at gene expression levels. We also did not observe any changes in the chromatin accessibility of fibroblast-specific genes during the early stages of reprogramming. This suggests that SCL is not directly repressing fibroblast identity; rather, it is directly inducing hematopoietic phenotype.

The TFs that induce direct reprogramming are thought to act as pioneer factors (Zaret and Carroll, 2011). Basic helix-loop-helix (bHLH) proteins can gain stable access to nucleosomes upon interaction with other adaptor proteins (Soufi et al., 2015). bHLH TF SCL interacts with non-DNA-binding factor LMO2, and this interaction is essential for SCL's DNA binding activity (Stanulovic et al., 2017). In our study, chromatin accessibility in early reprogramming cells showed that several regions that are completely inaccessible in control cells became accessible. These regions are predicted to be recognized by SCL, with the bHLH motif being the top scorer, confirming SCL's direct role in switching chromatin configuration. Indeed, SCL's known direct target genes, which were inaccessible in control cells, became open in early reprogramming cells. Together, these observations suggest a pioneering activity for SCL in reprogramming to iHPCs.

In conclusion, our findings indicate that SCL/LMO2-induced reprogramming of fibroblasts into HPCs occurs via an intermediate HE state. We have identified TF networks that play a crucial role in the reprogramming process. The relatively low efficiency of reprogramming is not a consequence of cell-intrinsic features of the starting fibroblast population or a failure of exogenous TFs to initiate the reprogramming process. Instead, a barrier to fibroblast reprogramming into HPCs is the conflicting cellular identities imposed by SCL and LMO2. Blocking

the undesired lineage commitment improved reprogramming efficiency to HPCs.

METHODS

Direct reprogramming of iSL MEFs to HPCs

Hematopoietic and endothelial cell-depleted E14.5 MEFs were seeded onto 0.1% gelatin-coated plates. The following day, the media was changed to hematopoietic media (1× Iscove's modified Dulbecco's medium, 10% FBS [Gibco], 10% protein-free hybridoma medium [Gibco], 1% L-glutamine, 1% penicillin-streptomycin, 25 μg/mL ascorbic acid, 4.5×10^{-4} M MTG, 180 μg/mL transferrin [R&D Systems], 50 ng/mL SCF [PeproTech], 25 ng/mL IL-3 [PeproTech], 25 ng IL-6 [PeproTech], 1% GM-CSF and 1% TPO conditioned media, 2,000 U/mL EPO, 10 ng/mL M-CSF, and 100 ng/mL Flt3 ligand [PeproTech]), and cells were treated with 1 μg/mL of doxycycline. Media was half changed every 4 days.

In vivo engraftment assay

MEFs were transduced with pSin-GFP lentivirus in the presence of 8 μg/mL polybrene (Sigma). 72 h post transduction, MEFs were directly reprogrammed to HPCs and sorted for c-Kit+GFP+ cells at day 14. The sorted cells were cultured in hematopoietic media for 3 days to facilitate expansion prior to injection (intrafemoral) into sublethally (2 Gy) irradiated NSG mice. Engraftment was monitored by GFP expression detected in peripheral blood. This *in vivo* experiment was performed according to protocols of the Institutional Animal Care and Use Committee at A*STAR.

RNA sequencing

MEFs were seeded onto 0.1% gelatin-coated T25 cm^2 flasks, 24 h prior to initiation of reprogramming with doxycycline. Cells were harvested on days 2, 4, and 14 of reprogramming, with day 14 cells sorted for c-KIT-positive cells. Library preparation was performed based on poly(A) selection using the SureSelect polyA kit (Agilent). The samples were sequenced on the NextSeq 500 (Illumina) generating 60 bp paired-end reads.

ATAC-seq assay

MEFs were seeded onto 0.1% gelatin-coated plates, and the following day, were treated with 1 μg/mL doxycycline for 48 and 96 h. 50,000 cells of each treated and control cells were centrifuged at 350 x g for 5 min at 4°C and washed in cold PBS. ATAC-seq experiment was performed as described previously (Buenrostro et al., 2015).

scRNA-seq

Reprogramming cells were harvested at days 2, 4, 8, and 14. On each day of sample harvesting, the cells were trypsinized, counted, and frozen in 10% DMSO containing FBS and stored in liquid nitrogen until library preparation steps. Libraries were prepared using the Chromium Next GEM Single cell 3′ Reagents Kit v.3.1 (10× Genomics) according to manufacturer's instructions. Libraries were sequenced using the NovaSeq 6000 platform on an S2 flow cell. sc-small interfering RNA-seq data were processed as preciously described (Gautam et al., 2021).

RESOURCE AVAILABILITY

Lead contact

Further information and requests for resources and reagents should be directed to and will be fulfilled by the lead contact, Kiran Batta (kiran.batta@manchester.ac.uk).

Material availability

ES line and mouse line generated in this study will be made available on request.

Data and code availability

Sequencing data were deposited into the Gene Expression Omnibus database under accession number GSE287568.

ACKNOWLEDGMENTS

The study was primarily funded by the University of Manchester and A*STAR Institute Singapore joint PhD program awarded to S.S. Epigenetics of Haematopoiesis group is funded by The Oglesby Charitable Trust. The Stem Cell Biology group is funded by CRUK Manchester Institute Core grants (no. C5759 and A27412). Y.-H.L. is supported by the NRF Investigatorship award NRFI2018-02; IAF-PP grant H1801a0021, NRF2019-THE002-0001, and NRF000407-00; NMRC grant OFIRG21nov-0088; A*STAR grants W22W3D 0007 and C211318012; and A*STAR BMRC Use-Inspired Basic Research award. The Developmental Haematopoiesis Group is supported by the Medical Research Council (MR/P000673/1; MR/T000384/1) and the Biotechnology and Biological Sciences Research Council (BB/R007209/1). F.M.R.A. is supported by CRUK grant numbers C5759/A20971 and C5759/A27412. K.H. is supported by the NMRC grant MOH-000937-00 and A*STAR grant C210812003. L.A.G. is supported by CRUK grant numbers C5759/A27445 and C147/A25254. We thank CRUK-MI core facilities including molecular biology core facilities and flow cytometry facilities. All illustrations in the manuscript were created with BioRender.com.

AUTHOR CONTRIBUTIONS

K.B., G.L., and Y.-H.L. conceived and designed the study. S.S., L.A.G., A.H.A., and K.B. performed all the experiments, analyzed the data, and wrote the manuscript. K.H. and F.M.R.A. performed computational analysis. V.K. and D.H.W. provided critical feedback on the manuscript.

DECLARATION OF INTERESTS

The authors declare no competing interests.

SUPPLEMENTAL INFORMATION

Supplemental information can be found online at https://doi.org/10.1016/j.stemcr.2025.102473.

Received: June 27, 2024
Revised: March 7, 2025
Accepted: March 8, 2025
Published: April 3, 2025

REFERENCES

Achim, K., Peltopuro, P., Lahti, L., Tsai, H.H., Zachariah, A., Astrand, M., Salminen, M., Rowitch, D., and Partanen, J. (2013). The role of Tal2 and Tal1 in the differentiation of midbrain GABAergic neuron precursors. Biol. Open *2*, 990–997. https://doi.org/10.1242/bio.20135041.

Batta, K., Florkowska, M., Kouskoff, V., and Lacaud, G. (2014). Direct reprogramming of murine fibroblasts to hematopoietic progenitor cells. Cell Rep. *9*, 1871–1884. https://doi.org/10.1016/j.celrep.2014.11.002.

Biddy, B.A., Kong, W., Kamimoto, K., Guo, C., Waye, S.E., Sun, T., and Morris, S.A. (2018). Single-cell mapping of lineage and identity in direct reprogramming. Nature *564*, 219–224. https://doi.org/10.1038/s41586-018-0744-4.

Bradley, C.K., Takano, E.A., Hall, M.A., Göthert, J.R., Harvey, A.R., Begley, C.G., and van Eekelen, J.A.M. (2006). The essential haematopoietic transcription factor Scl is also critical for neuronal development. Eur. J. Neurosci. *23*, 1677–1689. https://doi.org/10.1111/j.1460-9568.2006.04712.x.

Buenrostro, J.D., Wu, B., Chang, H.Y., and Greenleaf, W.J. (2015). ATAC-seq: A method for assaying chromatin accessibility genome-wideATAC-seq: A method for assaying chromatin accessibility genome-wideATAC-seq: A method for assaying chromatin accessibility genome-wide. Curr. Protoc. Mol. Biol. *109*, 21.29.1–21.29.9.

Chagraoui, H., Kristiansen, M.S., Ruiz, J.P., Serra-Barros, A., Richter, J., Hall-Ponselé, E., Gray, N., Waithe, D., Clark, K., Hublitz, P., et al. (2018). SCL/TAL1 cooperates with Polycomb RYBP-PRC1 to suppress alternative lineages in blood-fated cells. Nat. Commun. *9*, 5375. https://doi.org/10.1038/s41467-018-07787-6.

Cheng, H., Ang, H.Y.K., A El Farran, C., Li, P., Fang, H.T., Liu, T.M., Kong, S.L., Chin, M.L., Ling, W.Y., Lim, E.K.H., et al. (2016). Reprogramming mouse fibroblasts into engraftable myeloerythroid and lymphoid progenitors. Nat. Commun. *7*, 13396. https://doi.org/10.1038/ncomms13396.

Dignum, T., Varnum-Finney, B., Srivatsan, S.R., Dozono, S., Waltner, O., Heck, A.M., Ishida, T., Nourigat-McKay, C., Jackson, D.L., Rafii, S., et al. (2021). Multipotent progenitors and hematopoietic stem cells arise independently from hemogenic endothelium in the mouse embryo. Cell Rep. *36*, 109675. https://doi.org/10.1016/j.celrep.2021.109675.

El Omari, K., Hoosdally, S.J., Tuladhar, K., Karia, D., Hall-Ponselé, E., Platonova, O., Vyas, P., Patient, R., Porcher, C., and Mancini, E.J. (2013). Structural basis for LMO2-driven recruitment of the SCL:E47bHLH heterodimer to hematopoietic-specific transcriptional targets. Cell Rep. *4*, 135–147. https://doi.org/10.1016/j.celrep.2013.06.008.

Elefanty, A.G., Begley, C.G., Hartley, L., Papaevangeliou, B., and Robb, L. (1999). SCL expression in the mouse embryo detected with a targeted lacZ reporter gene demonstrates its localization to hematopoietic, vascular, and neural tissues. Blood *94*, 3754–3763.

Gautam, P., Hamashima, K., Chen, Y., Zeng, Y., Makovoz, B., Parikh, B.H., Lee, H.Y., Lau, K.A., Su, X., Wong, R.C.B., et al. (2021). Multi-species single-cell transcriptomic analysis of ocular compartment regulons. Nat. Commun. *12*, 5675. https://doi.org/10.1038/s41467-021-25968-8.

Goode, D.K., Obier, N., Vijayabaskar, M.S., Lie-A-Ling, M., Lilly, A.J., Hannah, R., Lichtinger, M., Batta, K., Florkowska, M., Patel, R., et al. (2016). Dynamic Gene Regulatory Networks Drive Hematopoietic Specification and Differentiation. Dev. Cell *36*, 572–587. https://doi.org/10.1016/j.devcel.2016.01.024.

Gratwohl, A., Pasquini, M.C., Aljurf, M., Atsuta, Y., Baldomero, H., Foeken, L., Gratwohl, M., Bouzas, L.F., Confer, D., Frauendorfer, K., et al. (2015). One million haemopoietic stem-cell transplants: a retrospective observational study. Lancet. Haematol. *2*, e91–e100. https://doi.org/10.1016/S2352-3026(15)00028-9.

Kyba, M., Perlingeiro, R.C.R., and Daley, G.Q. (2002). HoxB4 confers definitive lymphoid-myeloid engraftment potential on embryonic stem cell and yolk sac hematopoietic progenitors. Cell *109*, 29–37. https://doi.org/10.1016/s0092-8674(02)00680-3.

Latremoliere, A., Cheng, L., DeLisle, M., Wu, C., Chew, S., Hutchinson, E.B., Sheridan, A., Alexandre, C., Latremoliere, F., Sheu, S.H., et al. (2018). Neuronal-Specific TUBB3 Is Not Required for Normal Neuronal Function but Is Essential for Timely Axon Regeneration. Cell Rep. *24*, 1865–1879.e9. https://doi.org/10.1016/j.celrep.2018.07.029.

Levi-Montalcini, R. (1987). The nerve growth factor thirty-five years later. Vitro Cell Dev. Biol. *23*, 227–238. https://doi.org/10.1007/BF02623703.

Lis, R., Karrasch, C.C., Poulos, M.G., Kunar, B., Redmond, D., Duran, J.G.B., Badwe, C.R., Schachterle, W., Ginsberg, M., Xiang, J., et al. (2017). Conversion of adult endothelium to immunocompetent haematopoietic stem cells. Nature *545*, 439–445. https://doi.org/10.1038/nature22326.

Liu, Z., Wang, L., Welch, J.D., Ma, H., Zhou, Y., Vaseghi, H.R., Yu, S., Wall, J.B., Alimohamadi, S., Zheng, M., et al. (2017). Single-cell transcriptomics reconstructs fate conversion from fibroblast to cardiomyocyte. Nature *551*, 100–104. https://doi.org/10.1038/nature24454.

Nestorowa, S., Hamey, F.K., Pijuan Sala, B., Diamanti, E., Shepherd, M., Laurenti, E., Wilson, N.K., Kent, D.G., and Göttgens, B. (2016). A single-cell resolution map of mouse hematopoietic stem and progenitor cell differentiation. Blood *128*, e20–e31. https://doi.org/10.1182/blood-2016-05-716480.

Penalosa-Ruiz, G., Mulder, K.W., and Veenstra, G.J.C. (2020). The corepressor NCOR1 and OCT4 facilitate early reprogramming by suppressing fibroblast gene expression. PeerJ *8*, e8952. https://doi.org/10.7717/peerj.8952.

Pereira, C.F., Chang, B., Qiu, J., Niu, X., Papatsenko, D., Hendry, C.E., Clark, N.R., Nomura-Kitabayashi, A., Kovacic, J.C., Ma'ayan,

A., et al. (2013). Induction of a hemogenic program in mouse fibroblasts. Cell Stem Cell *13*, 205–218. https://doi.org/10.1016/j.stem.2013.05.024.

Porcher, C., Chagraoui, H., and Kristiansen, M.S. (2017). SCL/TAL1: a multifaceted regulator from blood development to disease. Blood *129*, 2051–2060. https://doi.org/10.1182/blood-2016-12-754051.

Riddell, J., Gazit, R., Garrison, B.S., Guo, G., Saadatpour, A., Mandal, P.K., Ebina, W., Volchkov, P., Yuan, G.C., Orkin, S.H., and Rossi, D.J. (2014). Reprogramming committed murine blood cells to induced hematopoietic stem cells with defined factors. Cell *157*, 549–564. https://doi.org/10.1016/j.cell.2014.04.006.

Sandler, V.M., Lis, R., Liu, Y., Kedem, A., James, D., Elemento, O., Butler, J.M., Scandura, J.M., and Rafii, S. (2014). Reprogramming human endothelial cells to haematopoietic cells requires vascular induction. Nature *511*, 312–318. https://doi.org/10.1038/nature13547.

Scialdone, A., Tanaka, Y., Jawaid, W., Moignard, V., Wilson, N.K., Macaulay, I.C., Marioni, J.C., and Göttgens, B. (2016). Resolving early mesoderm diversification through single-cell expression profiling. Nature *535*, 289–293. https://doi.org/10.1038/nature18633.

Setty, M., Kiseliovas, V., Levine, J., Gayoso, A., Mazutis, L., and Pe'er, D. (2019). Characterization of cell fate probabilities in single-cell data with Palantir. Nat. Biotechnol. *37*, 451–460. https://doi.org/10.1038/s41587-019-0068-4.

Solaimani Kartalaei, P., Yamada-Inagawa, T., Vink, C.S., de Pater, E., van der Linden, R., Marks-Bluth, J., van der Sloot, A., van den Hout, M., Yokomizo, T., van Schaick-Solernó, M.L., et al. (2015). Whole-transcriptome analysis of endothelial to hematopoietic stem cell transition reveals a requirement for Gpr56 in HSC generation. J. Exp. Med. *212*, 93–106. https://doi.org/10.1084/jem.20140767.

Soufi, A., Garcia, M.F., Jaroszewicz, A., Osman, N., Pellegrini, M., and Zaret, K.S. (2015). Pioneer transcription factors target partial DNA motifs on nucleosomes to initiate reprogramming. Cell *161*, 555–568. https://doi.org/10.1016/j.cell.2015.03.017.

Stanulovic, V.S., Cauchy, P., Assi, S.A., and Hoogenkamp, M. (2017). LMO2 is required for TAL1 DNA binding activity and initiation of definitive haematopoiesis at the haemangioblast stage. Nucleic Acids Res. *45*, 9874–9888. https://doi.org/10.1093/nar/gkx573.

Thoenen, H., and Edgar, D. (1985). Neurotrophic factors. Science *229*, 238–242. https://doi.org/10.1126/science.2409599.

Treutlein, B., Lee, Q.Y., Camp, J.G., Mall, M., Koh, W., Shariati, S.A., Sim, S., Neff, N.F., Skotheim, J.M., Wernig, M., and Quake, S.R. (2016). Dissecting direct reprogramming from fibroblast to neuron using single-cell RNA-seq. Nature *534*, 391–395. https://doi.org/10.1038/nature18323.

Van Handel, B., Montel-Hagen, A., Sasidharan, R., Nakano, H., Ferrari, R., Boogerd, C.J., Schredelseker, J., Wang, Y., Hunter, S., Org, T., et al. (2012). Scl represses cardiomyogenesis in prospective hemogenic endothelium and endocardium. Cell *150*, 590–605. https://doi.org/10.1016/j.cell.2012.06.026.

Wang, H., Yang, Y., Liu, J., and Qian, L. (2021). Direct cell reprogramming: approaches, mechanisms and progress. Nat. Rev. Mol. Cell Biol. *22*, 410–424. https://doi.org/10.1038/s41580-021-00335-z.

Wilson, N.K., Miranda-Saavedra, D., Kinston, S., Bonadies, N., Foster, S.D., Calero-Nieto, F., Dawson, M.A., Donaldson, I.J., Dumon, S., Frampton, J., et al. (2009). The transcriptional program controlled by the stem cell leukemia gene Scl/Tal1 during early embryonic hematopoietic development. Blood *113*, 5456–5465. https://doi.org/10.1182/blood-2009-01-200048.

Zaret, K.S., and Carroll, J.S. (2011). Pioneer transcription factors: establishing competence for gene expression. Genes Dev. *25*, 2227–2241. https://doi.org/10.1101/gad.176826.111.

Zheng, H., Chen, Y., Luo, Q., Zhang, J., Huang, M., Xu, Y., Huo, D., Shan, W., Tie, R., Zhang, M., et al. (2023). Generating hematopoietic cells from human pluripotent stem cells: approaches, progress and challenges. Cell Regen. *12*, 31. https://doi.org/10.1186/s13619-023-00175-6.

Zhou, Y., Liu, Z., Welch, J.D., Gao, X., Wang, L., Garbutt, T., Keepers, B., Ma, H., Prins, J.F., Shen, W., et al. (2019). Single-Cell Transcriptomic Analyses of Cell Fate Transitions during Human Cardiac Reprogramming. Cell Stem Cell *25*, 149–164.e9. https://doi.org/10.1016/j.stem.2019.05.020.

Zhou, Y., Wang, L., Vaseghi, H.R., Liu, Z., Lu, R., Alimohamadi, S., Yin, C., Fu, J.D., Wang, G.G., Liu, J., and Qian, L. (2016). Bmi1 Is a Key Epigenetic Barrier to Direct Cardiac Reprogramming. Cell Stem Cell *18*, 382–395. https://doi.org/10.1016/j.stem.2016.02.003.

Stem Cell Reports, Volume *20*

Supplemental Information

Competing dynamic gene regulatory networks involved in fibroblast re-programming to hematopoietic progenitor cells

Samiyah Shafiq, Kiyofumi Hamashima, Laura A. Guest, Ali H. Al-anbaki, Fabio M.R. Amaral, Daniel H. Wiseman, Valerie Kouskoff, Georges Lacaud, Yuin-Han Loh, and Kiran Batta

Document S1

Competing dynamic gene regulatory networks involved in fibroblast reprogramming to haematopoietic progenitor cells

Samiyah Shafiq, Kiyofumi Hamashima, Laura A Guest, Ali H Al-anbaki, Fabio M R Amaral, Daniel H Wiseman, Valerie Kouskoff, Georges Lacaud, Yuin-Han Loh, and Kiran Batta

Supplementary Figures:

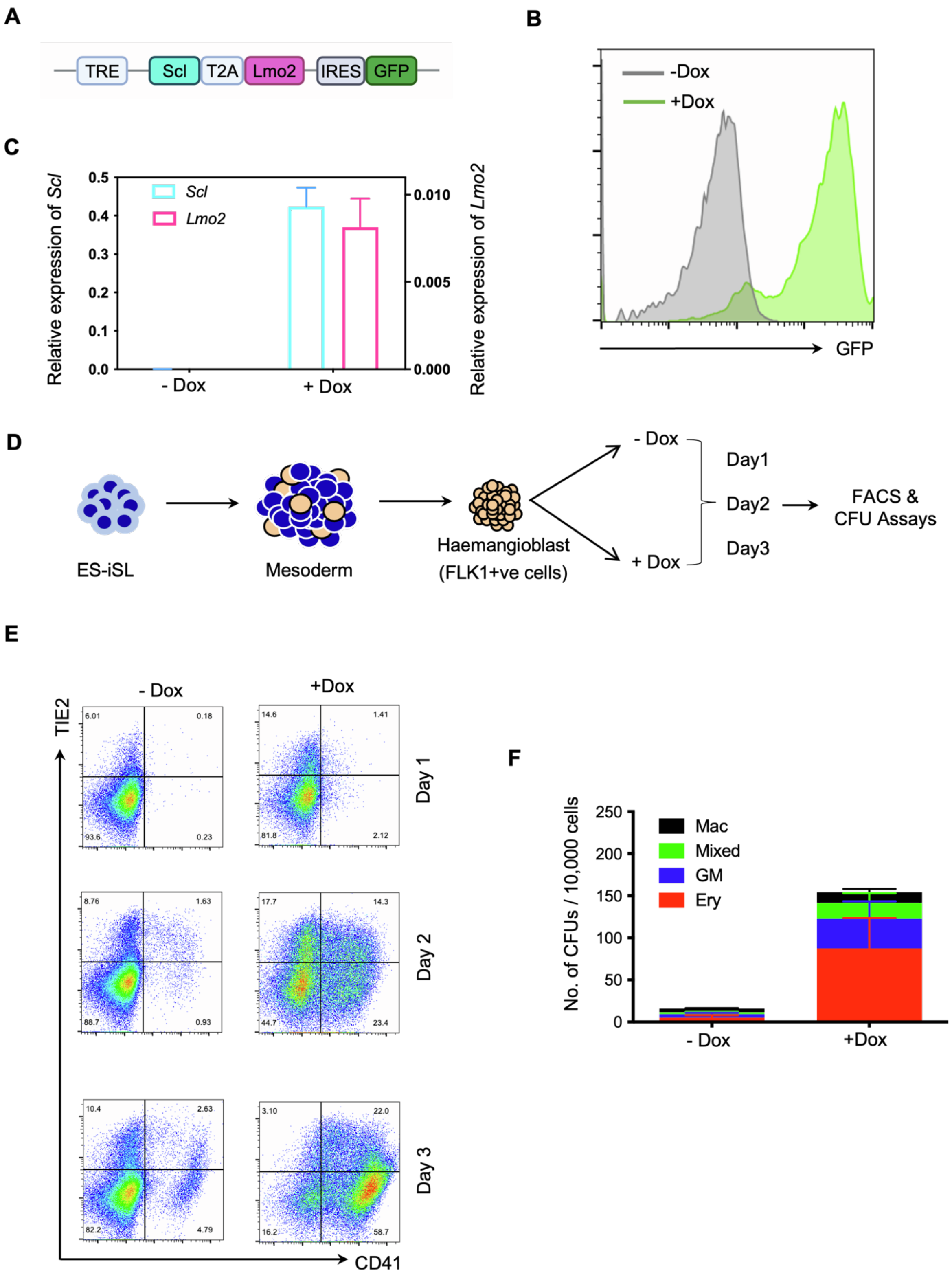

Figure S1

Figure S1: A. Schematic of the cassette incorporated into murine ES cells to generate inducible *Scl* and *Lmo2* ES line (iSL). TRE: Tetracycline response element **B.** Flow cytometry analysis measuring GFP expression of iSL ES line treated with or without doxycycline (Dox) for 24 hours. **C.** Relative expression of *Scl* and *Lmo2* in ES-iSL line treated with or without doxycycline for 24 hours with respect to housekeeping gene β-actin. Data is shown for a representative iSL-ES line performed in three technical replicates. **D**. Schematic representation of experimental methodology used to investigate the impact of *Scl* and *Lmo2* induction on differentiation of haemangioblasts to haematopoietic stem and progenitor cells. CFU: Colony forming unit **E**. Flow cytometry analysis of FLK1+ve haemangioblasts treated with or without doxycycline for 24, 48 and 72 hours**. F**. Number of different types of colonies observed from 10,000 plated cells taken at 24, 48 and 72 hours following with or without doxycycline induction in haemangioblast cultures from ES-iSL line. Data is shown from one experiment performed with three technical replicates. Mac: Macrophage, GM: Granulo-monocytic, Ery: erythroid.

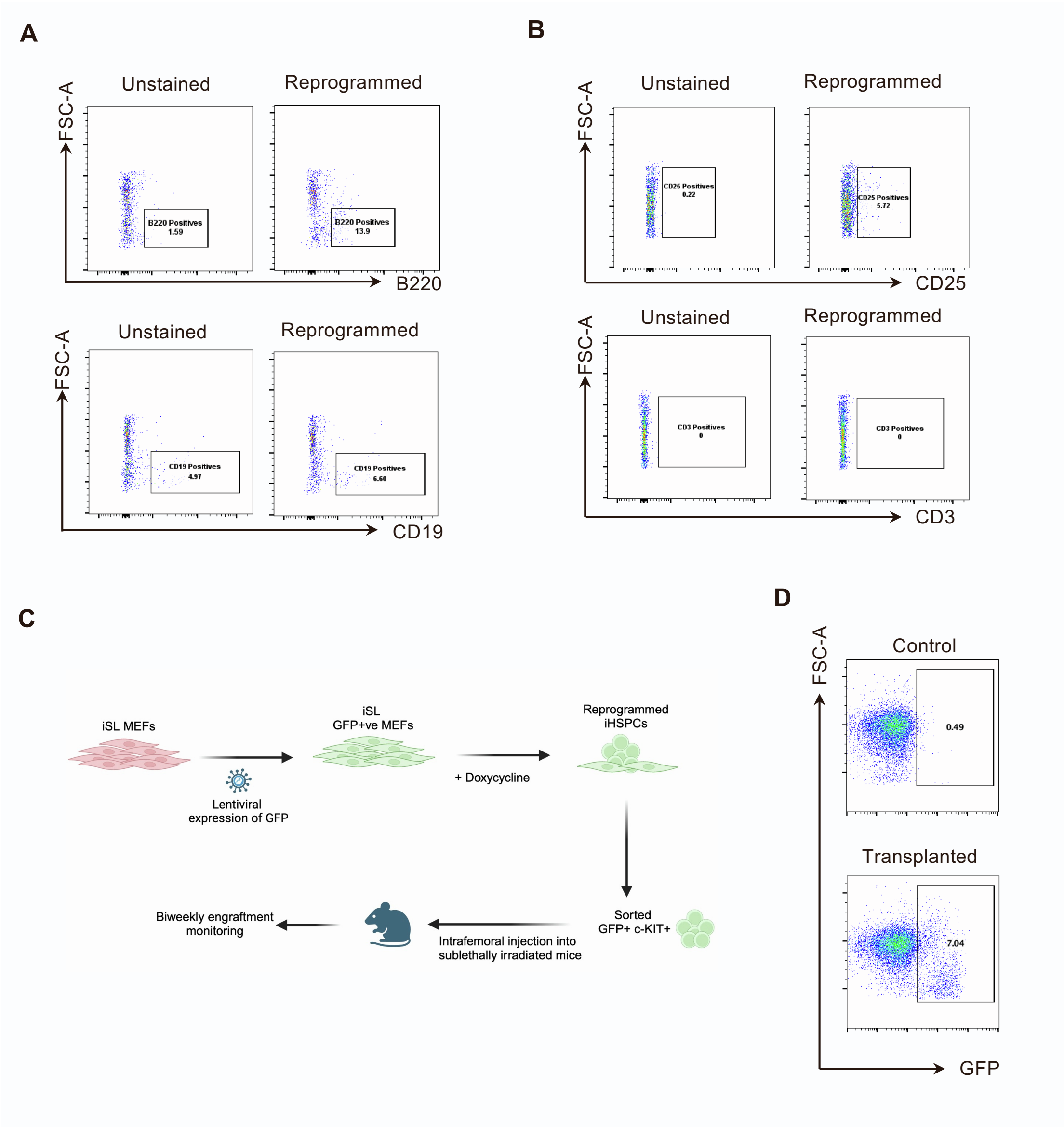

Figure S2

Figure S2: A. Flow cytometry analysis of day 14 reprogrammed cells when differentiated under culture conditions to promote B cell differentiation (day 15). **B.** Flow cytometry analysis of day 14 reprogrammed cells when differentiated under culture condition to promote T cell differentiation (day 15). **C.** Schematic for the in vivo engraftment study. Day 14.5 iSL-MEFs were transduced with GFP expressing lentivirus and the transduced MEFs were cultured in presence of doxycycline to initiate reprogramming. Reprogrammed cells were sorted for c-KIT and GFP expression and the double positive cells were expanded and injected intrafemorally into sub lethally irradiated mice (N=5). Blood samples were taken every two weeks to measure the levels of engraftment based on GFP positivity. **D.** Flow cytometry analysis of peripheral blood taken at week 5 from a representative mouse transplanted with reprogrammed cells and a control mouse injected with PBS.

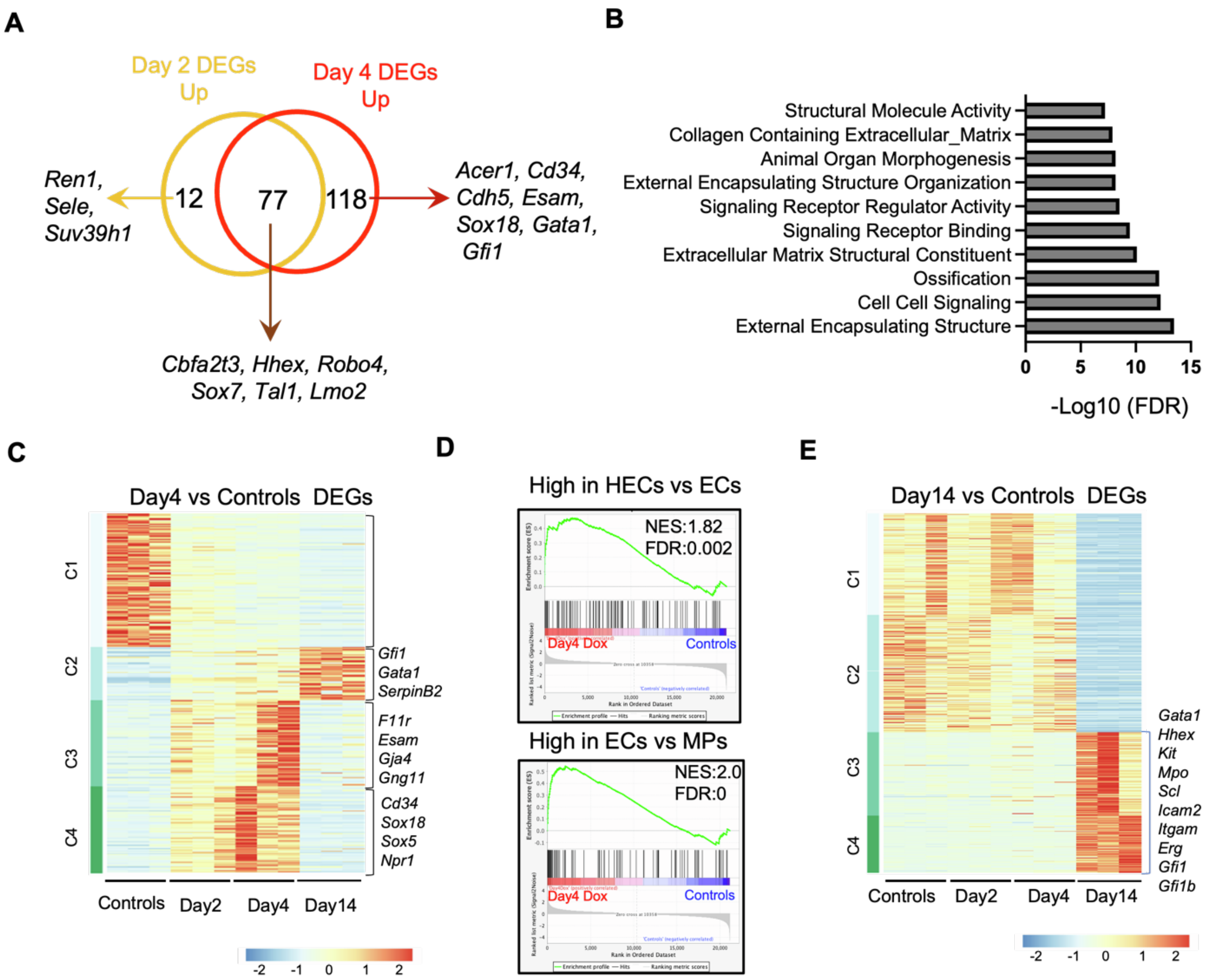

Figure S3

Figure S3: A. Venn diagram showing an overlap of genes that are upregulated in day 2 reprogramming cells with upregulated genes in day 4 reprogramming cells as compared with controls. **B.** GO analysis of downregulated genes in day 4 reprogramming cells as compared with controls. **C.** Heatmap showing the expression levels of DEGs in day 4 reprogramming cells as compared with control cells. Unsupervised K-means clustering identified 4 clusters (C1-C4). **D.** GSEA plots showing enrichment of genes upregulated in haemogenic endothelial cells (HECs) compared to endothelial cells (ECs) (Solaimani Kartalaei et al., 2015) (top) and genes upregulated in ECs with respect to mesodermal progenitor cells (MPs) (Scialdone et al., 2016) (bottom) in day4 reprogramming cells as compared with controls. **E.** Heatmap showing the expression levels of DEGs in day 14 c-KIT positive reprogramming cells as compared with control cells. Unsupervised K-means clustering identified 4 clusters (C1-C4).

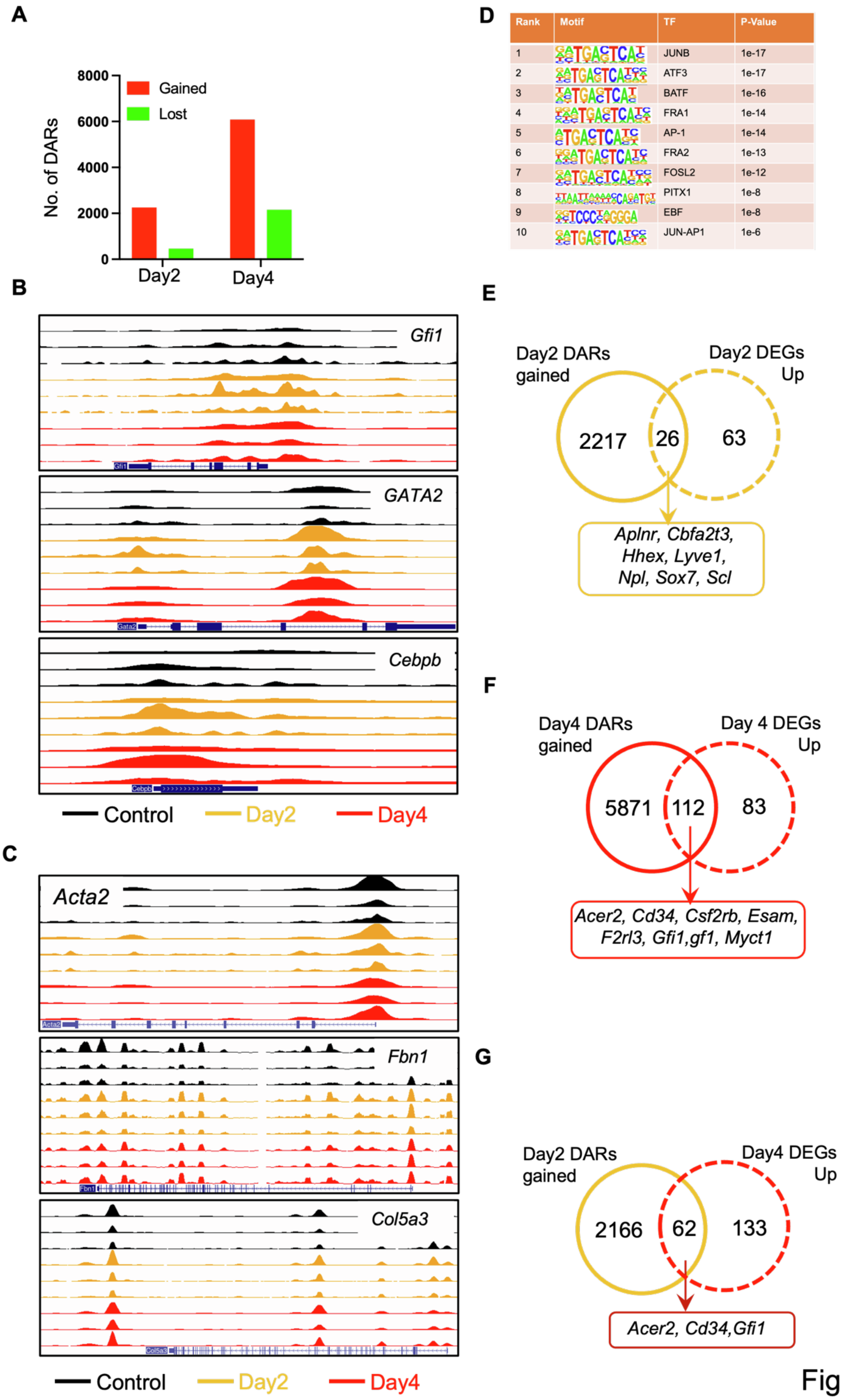
A
No. of DARs
8000
6000
4000
2000
0
Gained
Lost
Day2
Day4
B
Gfi1
GATA2
Cebpb
Control
Day2
Day4
C
Acta2
Fbn1
Col5a3
Control
Day2
Day4
D
Rank | Motif | TF | P-Value
1 JUNB 1e-17
2 ATF3 1e-17
3 BATF 1e-16
4 FRA1 1e-14
5 AP-1 1e-14
6 FRA2 1e-13
7 FOSL2 1e-12
8 PITX1 1e-8
9 EBF 1e-8
10 JUN-AP1 1e-6
E
Day2 DARs gained
Day2 DEGs Up
2217
26
63
Aplnr, Cbfa2t3, Hhex, Lyve1, Npl, Sox7, Scl
F
Day4 DARs gained
Day 4 DEGs Up
5871
112
83
Acer2, Cd34, Csf2rb, Esam, F2rl3, Gfi1,gf1, Myct1
G
Day2 DARs gained
Day4 DEGs Up
2166
62
133
Acer2, Cd34,Gfi1

Figure S4

Figure S4: A. Bar chart showing the number of differentially accessible regions (DARs) at day 2, and day 4 reprogramming cells as compared with control cells. **B & C.** UCSC browser plots depicting the chromatin accessibility at indicated haematopoietic (B) and fibroblast (C) genes in control, day 2 and day 4 reprogramming cells. **D.** Motif enrichment analysis in chromatin regions that lost accessibility in day 4 reprogramming cells as compared with controls. **E & F.** Venn diagram showing an overlap of genes that are mapped to regions gained accessibility in day 2 (E) and day 4 (F) reprogramming cells with upregulated genes in day 2 and day 4 reprogramming cells respectively**. G.** Venn diagram showing an overlap of genes that are mapped to regions that gained accessibility in day 2 reprogramming cells with upregulated genes in day 4 reprogramming cells.

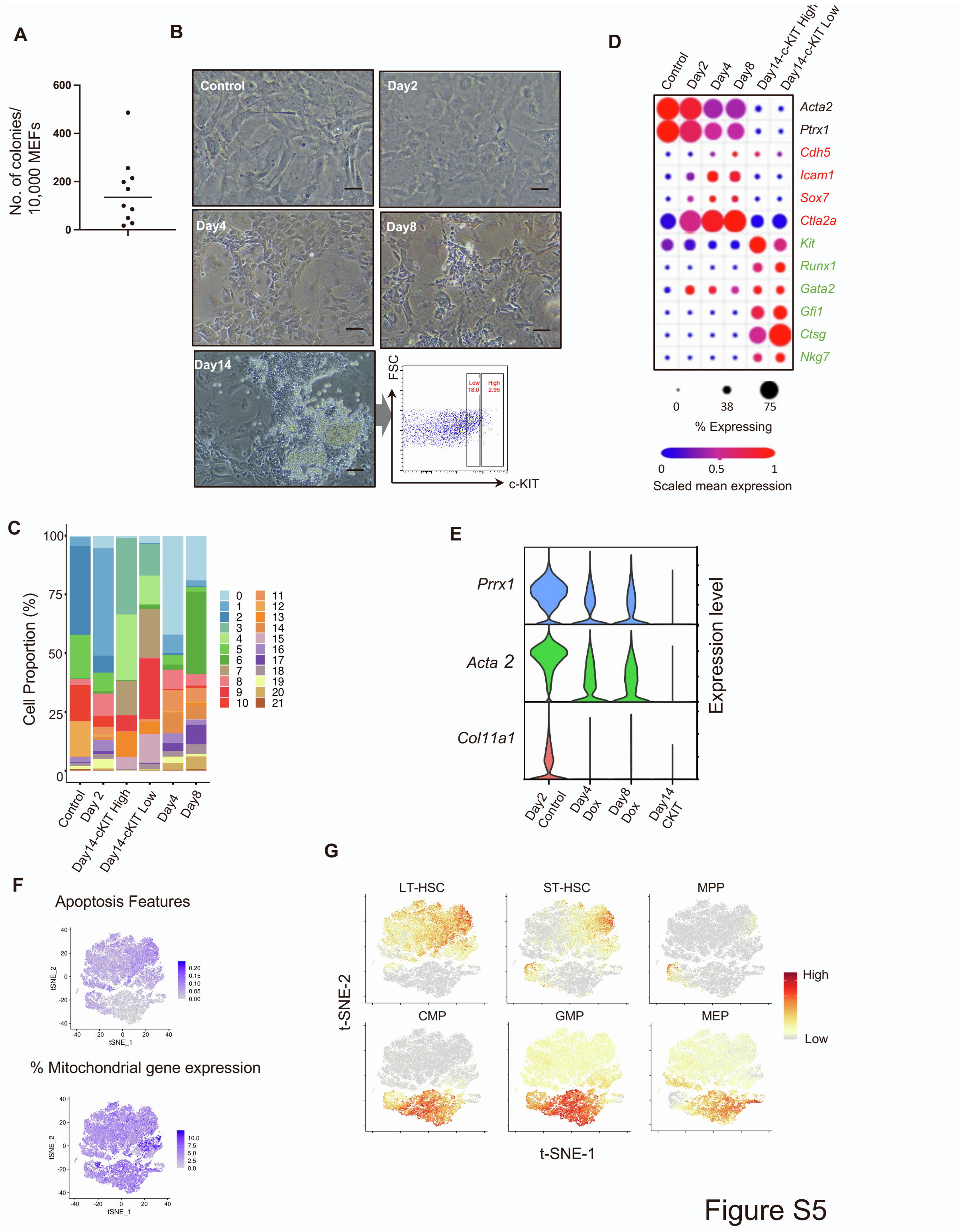
A
B
D
No. of colonies/ 10,000 MEFs
600
400
200
0
Control
Day2
Day4
Day8
Day14
FSC
c-KIT
Low
High
Control
Day2
Day4
Day8
Day14-c-KIT High
Day14-c-KIT Low
Acta2
Ptrx1
Cdh5
Icam1
Sox7
Ctla2a
Kit
Runx1
Gata2
Gfi1
Ctsg
Nkg7
0
38
75
% Expressing
0
0.5
1
Scaled mean expression
C
Cell Proportion (%)
100
75
50
25
0
Control
Day 2
Day14-cKIT High
Day14-cKIT Low
Day4
Day8
0 1 2 3 4 5 6 7 8 9 10 11 12 13 14 15 16 17 18 19 20 21
E
Prrx1
Acta 2
Col11a1
Expression level
Day2 Control
Day4 Dox
Day8 Dox
Day14 CKIT
F
Apoptosis Features
% Mitochondrial gene expression
tSNE_1
tSNE_2
G
LT-HSC
ST-HSC
MPP
CMP
GMP
MEP
High
Low
t-SNE-2
t-SNE-1

Figure S5

Figure S5. A. Number of haematopoietic colonies observed, following induction of SCL and LMO2, by 10,000 MEFs (N=10, MEFs from 10 different embryos). **B.** Brightfield images showing morphology of cells at day 2 control, day 2, 4, 8 and 14 doxycycline treated timepoints. Day 14 cells were sorted for the c-KIT positive (low and high) populations prior to harvesting for single cell library preparation. Scalebar 100 μm. **C.** Distribution of different clusters (identified by Seurat) in samples collected at different stages of reprogramming. **D.** Bubble plot showing the expression levels of indicated genes across time points during reprogramming. **E.** Violin plot showing the expression levels of indicated genes across time points during reprogramming **F.** Visualization of expression of apoptosis gene expression signature (top panel) or % mitochondrial gene expression (bottom panel) on t-SNE distribution. **G.** Visualization of expression of indicated HSPC specific gene expression signature (Nestorowa et al., 2016) on t-SNE distribution.

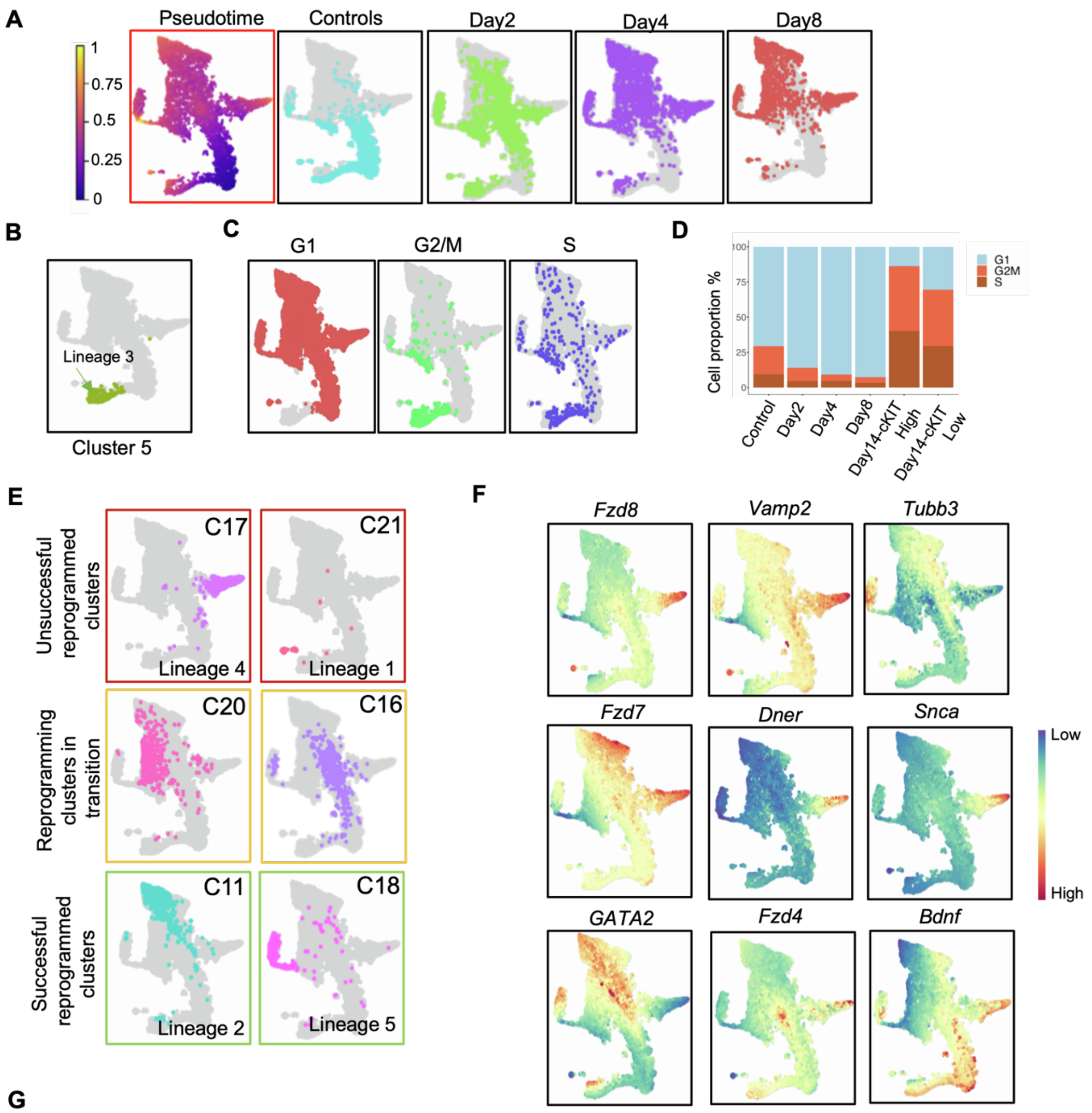

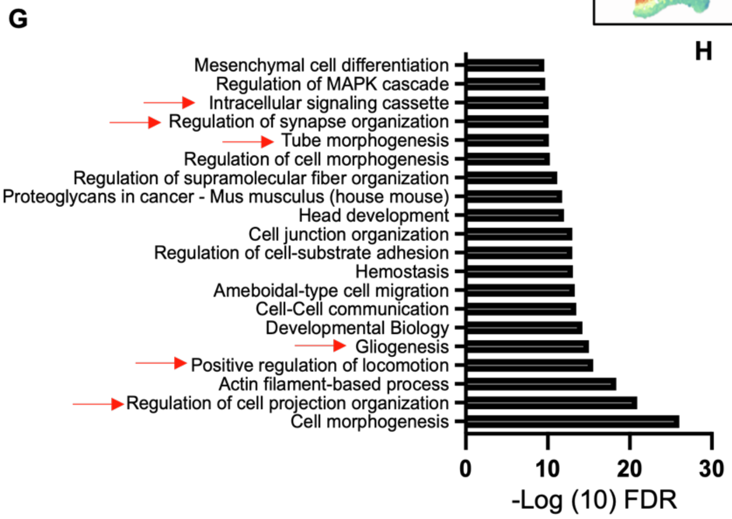

H

Chemical	Action
ICA-105665	K channel Opener
N-Acetly-5-hydroxytryptamine	Trkb Activator
LM22B-10	Trkb/c Activator
Diazoxide	ATP sensitive K channel activator
Arylsulfatase	Arylsulfatase activator
NGF	Trka activator
Brucine	Glycine blocker
GW 441756	Trka Inhibitor
Dihydrotestoterone	Arylsulfase Blocker

Figure S6

Figure S6: A. Cells within the Palantir trajectory plot coloured by sample identity. **B.** Cells in cluster 5 are highlighted in green within the trajectory. Lineage 3 is identified by a green arrow. **C.** Visualization of different cell cycle stage gene signatures overlaid within the trajectory. **D.** Breakdown of sample types by proportion of cells within each phase of cell cycle. **E.** Cells in unsuccessfully reprogrammed clusters (C17 and C21), transition clusters (C20 and C16) and successfully reprogrammed clusters (C11 and C18) are highlighted within the trajectory. **F.** Visualization of expression levels of different neuronal genes overlaid on the trajectory. **G.** Pathway analysis of genes uniquely expressed in Cluster 21 vs rest of the clusters. **H.** Small molecules modulators, together with their mode of action, screened to measure their impact on reprogramming efficiency are listed in the table.

Supplementary Tables:

Table S1: Differentially expressed genes in day 2 reprogramming cells vs controls (FDR <0.05). Related to Figure 3.

Table S2: Differentially expressed genes in day 4 reprogramming cells vs controls (FDR <0.05). Related to Figure 3.

Table S3: Differentially expressed genes in day 14 c-KIT positive reprogrammed cells vs controls (FDR <0.05). Related to Figure 3.

Table S4: Differentially accessible regions in day 2 reprogramming cells vs controls (FDR <0.05). Related to Figure 4.

Table S5: Differentially accessible regions in day 4 reprogramming cells vs controls (FDR <0.05). Related to Figure 4.

Supplementary Experimental Procedures:

iSL ES line generation: cDNA encoding SCL-T2A-LMO2 was cloned into modified KYBA vector p2LOX-IRES-GFP. This vector was used to generate iSL-ES line as described previously (Kyba et al., 2002).

ES cell culture and differentiation: Murine iSL-ES cell line was maintained and differentiated as described previously (Sroczynska et al., 2009). FLK1 positive haemangioblasts were sorted and cultured in presence or absence of doxycycline in medium containing 1X IMDM, 10% FBS, 0.5 mM Ascorbic Acid, 4.5×10^{-4}M MTG, 2 mM L-glutamine, 80 μg/ml transferrin and 50 μg/ml penicillin-streptomycin. Haemangioblast cultures were harvested and analysed for cell surface marker expression and clonogenic potential.

iSL mouse line generation: iSL-ES line was injected into blastocyst of pseudo pregnant mice and pups with highest percentage of chimerism were backcrossed with C57BL/6 to generate iSL mouse line. Genotyping was done to verify the presence of transgenic loci. Animal work was carried out as per Home Office Legislation under the Animal Scientific procedures Act (ASPA) 1986 and was approved by the Animal Welfare and Ethics Review Body (AWERB) of the Cancer Research UK Manchester Institute.

MEF isolation from iSL mouse line: MEFs were isolated by dissecting E14.5 embryos under a stereomicroscope. The head and internal organs were discarded, and the remaining tissues of each individual embryo were washed in sterile phosphate-buffered saline (PBS). Subsequently, the tissues were placed in a well of a 6-well plate and finely minced into a slurry using a scalpel. To facilitate homogenisation, 2 ml of trypsin-EDTA (Gibco) solution was added, and the mixture was incubated for 5 minutes at 37°C. Repeated pipetting was performed to aid in the homogenisation process. The resulting cell suspension was transferred to a T175 cm2 flask containing 30 ml of Iscove's Modified Dulbecco's Medium (IMDM) (Gibco) supplemented with 20% fetal bovine serum (FBS, Gibco), 1% penicillin/streptomycin (P/S, Invitrogen), 1% L-Glutamine (L-Glut, Invitrogen) and 20 µm of alpha-monothioglycerol (MTG, Sigma), hereafter referred to as MEF media. The cells were cultured at 37°C at 5% O2 overnight, and the next day, the media was changed to remove any unattached material. The remaining adherent cells were cultured until they reached confluency and then frozen in 10% dimethyl sulfoxide (DMSO, Sigma) in FBS.

Depletion of haematopoietic and endothelial cells from MEF cultures: Before conducting direct reprogramming experiments, the MEFs underwent a depletion process to remove haematopoietic and endothelial contaminant cells. This was achieved through magnetically activated cell sorting (MACS). The cells were subjected to a 20-minute staining process on ice, using a master mix of biotin-conjugated antibodies specific to CD45, c-KIT, CD41, and CD34 (Biolegend) in PBS supplemented with 5% FBS and 0.5 mM Ethylenediaminetetraacetic acid (EDTA, Sigma) (referred to as FACS buffer from here onwards). Two washes were performed by adding 2 ml of MACS buffer (0.5% BSA and 2mM EDTA in PBS) and centrifuging at 350 g for 5 mins at 4°C each time. Subsequently, the cells were resuspended in 80 µl of MACS buffer, 20 µl of anti-biotin microbeads (Miltenyi Biotec) were added, and cells were incubated for an additional 20 mins on ice. The cells were washed again twice as before and resuspended in 500 µl of MACS buffer after the second spin. In parallel, the MACS® MS separation column (Miltenyi Biotec) was pre-prepared by fitting it onto the OctoMACS™ Separator system and rinsing the empty column with 500 µl of MACS buffer. The cell suspension was then applied to the column after which the column was washed 3 times with 500 µl of MACS buffer to further remove any unlabelled cells. The flow through containing the fibroblasts was collected and cells bound to the column (haematopoietic and endothelial contaminants) were

flushed by adding 1 ml of MACS buffer to the column and inserting the plunger to collect the labelled cells. The cells were washed in PBS and a minimum of 30,000 of unsorted, flow through and eluate cells were collected for flow cytometry analysis to confirm the purity of the fibroblast population before being utilized in downstream experiments.

Flow cytometry and cell sorting: Surface marker expression was analysed using the BD LSR II (BD biosciences) or the NovoCyte Penteon Flow Cytometer (Agilent). To prepare the cells for analysis a minimum of 40,000 cells were washed in FACS buffer. Pelleted cells were resuspended in antibodies that were diluted in FACS buffer according to the manufacturer's instructions for each antibody. Cells were stained with different combinations of the following antibodies: CD45-PerCp Cy5.5 (1:100, Miltenyi), CD41-Pecy7 (1:20, Miltenyi), c-KIT-APC (1:100, Miltenyi), CD11b (1:100, Miltenyi), CD71 (1:100, Miltenyi), for 25 minutes on ice. After staining the cells were washed twice in FACS buffer and resuspended in 350 μl of FACS buffer. To aid the flow cytometry analysis and compensation, single stained controls were also prepared using cells for GFP and DAPI single stained controls and UltraComp beads™ for all the remaining markers. For GFP controls, day 2 dox treated cells were harvested. The beads were stained on ice for 20 minutes after which they were washed in 1 ml FACS buffer and resuspended in 350 μl of FACS buffer. Flow cytometry was then performed on the BD LSR II or NovoCyte Penteon Flow Cytometer and surface marker expression was analysed on the FlowJo software.

Cell sorting was performed primarily on the Aria Fusion. To sort the cells, they were stained as described and analysed. The cells were mostly sorted for the c-KIT$^+$ population after successful reprogramming; live, single, c-KIT positive cells were gated and sorted into new FACS tubes for downstream use.

Western blot: MEFs were cultured for 48 hours in the presence of 1 μg/ml doxycycline, washed twice with cold PBS and incubated for 30 minutes at 4°C with continuous mixing in RIPA Buffer (150 mM NaCl, 1% Nonidet P-40, 0.1% SDS, 25 mM Tris (pH 7.4) and 1% sodium deoxycholate) to lyse the cells. The cell lysate mixture was centrifuged for 20 minutes at 12000 rpm at 4°C, and the supernatant containing the lysate was collected. The protein extract was quantified using the Pierce™ BCA Protein Assay Kit (Thermo Scientific) and 10-100ug of protein used NuPAGE™ LDS sample buffer (Invitrogen) was added to the lysates of each sample and incubated at 70°C for 10 minutes to denature the proteins. The whole protein extracts were loaded onto a NuPAGE™ 4 to 12%, Bis-Tris, 1.0-1.5 mm, protein gel (Invitrogen) and separated using the NuPAGE™ SDS-PAGE Gel System (Invitrogen) in pre-cooled MOPS buffer (1M MOPS, 1M Tris-Base, 2% SDS, 20.5mM EDTA). The gel was mounted onto a nitrocellulose membrane and sandwiched into a transfer cassette which was run at 100 V for 60 minutes at 4°C. After the transfer was complete the

membrane was stained with Ponceau stain (Sigma) to confirm the successful transfer. Blocking of the membrane was performed in 5% milk for 3 hours at room temperature (RT) after which primary antibody was added at a 1:1000 dilution and the membranes were incubated overnight at 4°C. The following morning the membranes were washed in PBS with 0.1% tween (PBS-T) 3 times for 10 minutes each. Subsequently, secondary antibody incubation was performed for 1 hour at RT. The membranes were washed again 3 times in PBS-T for 10 minutes each and tapped dry with a paper towel. Pierce™ ECL Western Blotting Substrate (Thermo Scientific) at a 1:1 ratio was poured onto the membranes and allowed to incubate for 1-2 minutes after which the ChemiDoc imagine system (Bio-Rad) was used to visualise the bands.

Quantitative RT-PCR: RNA was extracted from cells using the Monarch Total RNA Miniprep Kit (NEB) according to kit instructions. Up to 500 ng of RNA was then reverse transcribed using the iScript™ cDNA Synthesis Kit (BioRad). For the qRT-PCR reaction, 2 ng of cDNA was added to a master mix containing 5 µl BlitzAmp Hotstart qPCR Master Mix (MiRXES), 0.2 µl of 5 µM qPCR primers and nuclease free water (Invitrogen). Each reaction was performed in a total volume of 10 µl with three technical replicates per sample. For normalisation purposes β -Actin or GAPDH were always included as house-keeping genes and internal no template control. qRT-PCR results were analysed using the Delta-delta Ct method ($2^{-\Delta\Delta Ct}$).

CFU assay: Colony forming assay was performed on directly reprogrammed HPCs by resuspending 10,000 cells in 100 µl IMDM media supplemented with 2% FBS. The cells were then added to 900 µl of MethoCult ™ (STEMCELL Technologies) and supplemented with 25 ng IL-3, 25 ng IL-6 and 50 ng SCF. A 16G needle and 1 ml syringe were then used to dispense 300 µl of the cell suspension into each well of a 24-well tissue culture treated plate in triplicate. PBS was added to the surrounding wells to prevent the methylcellulose from drying out. Colonies were counted and scored on day 14 based on standard morphological criteria.

Morphological Staining: Approximately 10,000 cells were spun onto a microscope slide using the Thermo Cytospin 4 Cyto centrifuge (Thermo). The slides were left to airdry which was followed by fixation in methanol for 5-10 mins at room temperature. Slides were submerged in May Grunwald stain (Sigma) freshly diluted with equal volumes of Sorenson's buffer (1x KH2PO4, 1x Na2HPO4 in ddH2O) for 15-20 minutes. Immediately after, Giemsa stain (Sigma) was diluted in 1:10 in Sorenson's buffer and used to stain the slides for 30 minutes. Following washes in running tap water and Sorenson's buffer, the slides were left to air dry and mounted using DPX neutral mounting medium (Sigma).

Intrafemoral injections: To perform intrafemoral injection of reprogrammed cells, the mice were kept anaesthetized using isoflurane whilst cells were injected into the femoral cavity. Before injection the area of injection was disinfected with 70% ethanol and betadine surgical scrub. To stabilise the joint for injection the knee was flexed to a 90-degree angle and a small incision was made using a scalpel to increase visibility of the bone. Next, a 26-gauge insulin needle loaded with a maximum of 200,000 cells was inserted into the joint surface of the femur through the patellar tendon and introduced into the bone marrow cavity where the cells were then injected. The skin was then sutured with 4-0 Sofsilk^{TM} sutures (Medtronic) and the mice were observed closely for the first week post-surgery during which time painkillers were administered for 3 days and antibiotics for 5 days to aid recovery and prevent infection.

Assay for Transposase-accessible chromatin with sequencing (ATAC-Seq): MEFs were seeded onto 0.1% gelatin coated plates and the following day were treated with 1 ng/ml doxycycline for 48 and 96 hours. 50,000 cells of each treated and control cells were centrifuged at 350 x g for 5 minutes at 4°C and washed in cold PBS. The cell pellet was resuspended in 50 µl transposase mixture containing 25 µl 2x TD Buffer, 2.5 µl transposase enzyme, 0.01 % digitonin and 22 µl water. The resuspended cell pellet was incubated at 37°C for 60 minutes at 300 rpm. Subsequently, samples were purified using the MinElute PCR purification kit (Qiagen), according to manufacturer instructions. The elution buffer was pre-warmed to 37 °C and during the final elution step, the buffer was left to incubate on the columns for an additional 5 minutes before the final elution spin step. The purified DNA samples were stored at -20°C until library preparation was performed as described (Buenrostro et al., 2015). Final libraries were sequenced (2 x 60) on the Illumina NovaSeq 6000 platform.

Data analysis: The sequencing reads were mapped to the mouse mm9 genome using STAR with the following parameters: --alignIntronMax 1 --alignEndsType EndToEnd. Peak calling was performed by MACS2 (Zhang et al., 2008). ATAC-seq read counts for each sample were then calculated using featureCounts (Liao et al., 2014). DESeq2 (Love et al., 2014) was used to visualize the PCA plot and identify the differential peaks using cutoff p-value <0.05. ChIPseeker R package (Yu et al., 2015) was used to annotate genomic features of determined peaks with the parameter ‘tssRegion=c (-5000, 5000)’. The peaks located in the promoter and enhancer (http://enhanceratlas.org/data/download/enhancer/mm/MEF_E13.5.bed) regions were selected for the motif analysis. Homer (Heinz et al., 2010) findMotifsGenome.pl was used to identify TF motifs enriched with the parameter ‘-size 2000’. Homer ‘makeTagDirectory’ followed by and ‘makeUCSCfile’ were used to create bedGraph files for the visualization of ATAC peaks on the UCSC genome browser.

Single Cell RNA Sequencing: Single-cell RNA-seq data was processed as preciously described (Gautam et al., 2021). Briefly, we used counts modes of cellragner (Zheng et al., 2017) to generate read count matrices from the fastq files. The reference genome index of mm10 was downloaded from 10x Genomics. Count and fragment data were imported into Seurat (Hao et al., 2021) objects, and the QC was performed to remove outlier based on the total UMI, detected gene number and % mitochondrial genes. Major cell classes were manually annotated based on the cluster-specific markers. For the identification of cluster biomarkers, we used the Seurat 'FindMarkers' function (min.pct = 0.25, logfc.threshold = 0.25), which detects DEGs for each cluster. Module scores (related to Figure S5D) were calculated using Seurat AddModuleScore function with cluster 3, 4, 7, 9, 13 and 15 DEG sets, and superimposed on the tSNE plot of single-cell RNA-seq cells from published studies (Nestorowa et al., 2016). HPC reprogramming trajectories were inferred by Palantir (Setty et al., 2019) according to the standard workflow. The enrichment of Reactome pathway genes in our Seurat clusters was analysed using ReactomeGSA (Griss et al., 2020) R package with default parameters.

References

Buenrostro, J.D., Wu, B., Chang, H.Y., and Greenleaf, W.J. (2015). ATAC-seq: A Method for Assaying Chromatin Accessibility Genome-Wide. Curr Protoc Mol Biol *109*, 21 29 21-21 29 29. 10.1002/0471142727.mb2129s109.

Gautam, P., Hamashima, K., Chen, Y., Zeng, Y., Makovoz, B., Parikh, B.H., Lee, H.Y., Lau, K.A., Su, X., Wong, R.C.B., et al. (2021). Multi-species single-cell transcriptomic analysis of ocular compartment regulons. Nat Commun *12*, 5675. 10.1038/s41467-021-25968-8.

Griss, J., Viteri, G., Sidiropoulos, K., Nguyen, V., Fabregat, A., and Hermjakob, H. (2020). ReactomeGSA - Efficient Multi-Omics Comparative Pathway Analysis. Mol Cell Proteomics *19*, 2115-2125. 10.1074/mcp.TIR120.002155.

Hao, Y., Hao, S., Andersen-Nissen, E., Mauck, W.M., 3rd, Zheng, S., Butler, A., Lee, M.J., Wilk, A.J., Darby, C., Zager, M., et al. (2021). Integrated analysis of multimodal single-cell data. Cell *184*, 3573-3587 e3529. 10.1016/j.cell.2021.04.048.

Heinz, S., Benner, C., Spann, N., Bertolino, E., Lin, Y.C., Laslo, P., Cheng, J.X., Murre, C., Singh, H., and Glass, C.K. (2010). Simple combinations of lineage-determining transcription factors prime cis-regulatory elements required for macrophage and B cell identities. Mol Cell *38*, 576-589. 10.1016/j.molcel.2010.05.004.

Kyba, M., Perlingeiro, R.C., and Daley, G.Q. (2002). HoxB4 confers definitive lymphoid-myeloid engraftment potential on embryonic stem cell and yolk sac hematopoietic progenitors. Cell *109*, 29-37. 10.1016/s0092-8674(02)00680-3.

Liao, Y., Smyth, G.K., and Shi, W. (2014). featureCounts: an efficient general purpose program for assigning sequence reads to genomic features. Bioinformatics *30*, 923-930. 10.1093/bioinformatics/btt656.

Love, M.I., Huber, W., and Anders, S. (2014). Moderated estimation of fold change and dispersion for RNA-seq data with DESeq2. Genome Biol *15*, 550. 10.1186/s13059-014-0550-8.

Nestorowa, S., Hamey, F.K., Pijuan Sala, B., Diamanti, E., Shepherd, M., Laurenti, E., Wilson, N.K., Kent, D.G., and Gottgens, B. (2016). A single-cell resolution map of mouse hematopoietic stem and progenitor cell differentiation. Blood *128*, e20-31. 10.1182/blood-2016-05-716480.

Scialdone, A., Tanaka, Y., Jawaid, W., Moignard, V., Wilson, N.K., Macaulay, I.C., Marioni, J.C., and Gottgens, B. (2016). Resolving early mesoderm diversification through single-cell expression profiling. Nature *535*, 289-293. 10.1038/nature18633.

Setty, M., Kiseliovas, V., Levine, J., Gayoso, A., Mazutis, L., and Pe'er, D. (2019). Characterization of cell fate probabilities in single-cell data with Palantir. Nat Biotechnol *37*, 451-460. 10.1038/s41587-019-0068-4.

Solaimani Kartalaei, P., Yamada-Inagawa, T., Vink, C.S., de Pater, E., van der Linden, R., Marks-Bluth, J., van der Sloot, A., van den Hout, M., Yokomizo, T., van Schaick-Solerno, M.L., et al. (2015). Whole-transcriptome analysis of endothelial to hematopoietic stem cell transition reveals a requirement for Gpr56 in HSC generation. J Exp Med *212*, 93-106. 10.1084/jem.20140767.

Sroczynska, P., Lancrin, C., Pearson, S., Kouskoff, V., and Lacaud, G. (2009). In vitro differentiation of mouse embryonic stem cells as a model of early hematopoietic development. Methods Mol Biol *538*, 317-334. 10.1007/978-1-59745-418-6_16.

Yu, G., Wang, L.G., and He, Q.Y. (2015). ChIPseeker: an R/Bioconductor package for ChIP peak annotation, comparison and visualization. Bioinformatics *31*, 2382-2383. 10.1093/bioinformatics/btv145.

Zhang, Y., Liu, T., Meyer, C.A., Eeckhoute, J., Johnson, D.S., Bernstein, B.E., Nusbaum, C., Myers, R.M., Brown, M., Li, W., and Liu, X.S. (2008). Model-based analysis of ChIP-Seq (MACS). Genome Biol *9*, R137. 10.1186/gb-2008-9-9-r137.

Zheng, G.X., Terry, J.M., Belgrader, P., Ryvkin, P., Bent, Z.W., Wilson, R., Ziraldo, S.B., Wheeler, T.D., McDermott, G.P., Zhu, J., et al. (2017). Massively parallel digital transcriptional profiling of single cells. Nat Commun *8*, 14049. 10.1038/ncomms14049.
